# Simultaneous Evaluation of Diagnostic Assays for Pharyngeal and Rectal *Neisseria gonorrhoeae* and *Chlamydia trachomatis* Using a Master Protocol

**DOI:** 10.1093/cid/ciz1105

**Published:** 2019-11-17

**Authors:** Sarah B Doernberg, Lauren Komarow, Thuy Tien T Tran, Zoe Sund, Mark W Pandori, David Jensen, Ephraim L Tsalik, Carolyn D Deal, Henry F Chambers, Vance G Fowler, Scott R Evans, Robin Patel, Jeffrey D Klausner

**Affiliations:** 1 University of California, San Francisco, California, USA; 2 The George Washington University Biostatistics Center, Rockville, Maryland, USA; 3 Duke University, Durham, North Carolina, USA; 4 Alameda County Department of Public Health, Oakland, California, USA; 5 Durham Veterans Affairs Health Care System, Durham, North Carolina, USA; 6 National Institute of Allergy and Infectious Diseases, Bethesda, Maryland, USA; 7 Mayo Clinic, Rochester, Minnesota, USA; 8 University of California, Los Angeles, California, USA

**Keywords:** nucleic acid amplification techniques, *Neisseria gonorrhoeae*, *Chlamydia trachomatis*, sexually transmitted infections, diagnostic techniques and procedures

## Abstract

**Background:**

Pharyngeal and rectal *Neisseria gonorrhoeae* and *Chlamydia trachomatis* play important roles in infection and antibacterial resistance transmission, but no US Food and Drug Administration (FDA)–cleared assays for detection at these sites existed prior to this study. The objective was to estimate performance of assays to detect those infections in pharyngeal and rectal specimens to support regulatory submission.

**Methods:**

We performed a cross-sectional, single-visit study of adults seeking sexually transmitted infection testing at 9 clinics in 7 states. We collected pharyngeal and rectal swabs from participants. The primary outcome was positive and negative percent agreement for detection of *N. gonorrhoeae* and *C. trachomatis* for 3 investigational assays compared to a composite reference. Secondary outcomes included positivity as well as positive and negative predictive values and likelihood ratios. Subgroup analyses included outcomes by symptom status and sex.

**Results:**

A total of 2598 participants (79% male) underwent testing. We observed *N. gonorrhoeae* positivity of 8.1% in the pharynx and 7.9% in the rectum and *C. trachomatis* positivity of 2.0% in the pharynx and 8.7% in the rectum. Positive percent agreement ranged from 84.8% to 96.5% for different anatomic site infection combinations, whereas negative percent agreement was 98.8% to 99.6%.

**Conclusions:**

This study utilized a Master Protocol to generate diagnostic performance data for multiple assays from different manufacturers in a single study population, which ultimately supported first-in-class FDA clearance for extragenital assays. We observed very good positive percent agreement when compared to a composite reference method for the detection of both pharyngeal and rectal *N. gonorrhoeae* and *C. trachomatis*.

**Clinical Trials Registration:**

NCT02870101.

## Teaser

This cross-sectional study of 2598 participants evaluated diagnostic performance of 3 assays for pharyngeal and rectal gonorrhea and chlamydia detection, leading to Food and Drug Administration clearance. Positive percentage agreement ranged from 84.8% to 96.5%, and negative percentage agreement from 98.8% to 99.6%.

**(See the Editorial Commentary by Berry and Ghanem on pages 2323–5.)**

Molecular diagnostic assays have transformed the field of infectious diseases, allowing for swift and highly sensitive detection of organisms previously challenging to diagnose, including infections due to *Neisseria gonorrhoeae* (NG) and *Chlamydia trachomatis* (CT) [1]. Extragenital sites such as the pharynx and rectum are important reservoirs for disease transmission. Pharyngeal NG infection may serve as an important source of NG antimicrobial resistance [2–6]. The prompt diagnosis and treatment of infection interrupts transmission pathways, thus decreasing the risk for further spread to sex partners, acquisition of human immunodeficiency virus (HIV) infection, and the spread of NG resistance [7–9]. Nucleic acid amplification tests (NAATs) have become the gold standard for detecting NG and CT infections in the urogenital tract but, prior to this study, none were cleared for marketing by the US Food and Drug Administration (FDA) for use in pharyngeal or rectal sites, despite widespread use and recommendation by the US Centers for Disease Control and Prevention (CDC) [2, 10]. Therefore, such testing was only available in select clinical laboratories and reference laboratories that have completed verification studies according to the Clinical Laboratory Improvement Amendments [11]. FDA clearance will extend availability of those assays to laboratories serving public health and clinical settings and thus fill an important gap in the prevention and control of those infections.

Demonstrating the performance of new NAATs that have greater analytic sensitivity than the traditional culture-based reference method poses unique challenges, and comparison to an imperfect standard might bias the performance estimates for an assay under consideration [12–14]. Instead, a composite reference method made up of multiple, independent assays offers a better approach [15]. However, creating a composite reference method poses additional challenges, including obtaining cooperation among multiple device manufacturers. If successful, the outcome could be the validation of multiple assays, each of which is both independently evaluated and used as part of the composite reference method for the other assays [16]. We and others have previously described an innovative study design known as a “Master Protocol,” which simultaneously assesses the performance of multiple interventions or, as reported herein, multiple diagnostics, in a single study population [17, 18]. Here, we collaborated with multiple manufacturers—with FDA consultation—to establish an agreed-upon composite reference method to assess diagnostic performance of 3 different assays for the detection of pharyngeal and rectal NG and CT infections. The use of the Master Protocol allowed a single subject’s samples to be used in the evaluation of 3 different diagnostic assays with the ultimate purpose of providing data to the assay manufacturers to support a regulatory submission.

## METHODS

### Study Design, Setting, and Population

This was a cross-sectional, single-visit study of consecutive adults seeking sexually transmitted infection (STI) testing at 9 clinics offering STI testing (2 community clinics, 4 public health clinics, 2 reproductive health clinics, and 1 clinic serving sexual and gender minority populations) located in 7 states (California, Colorado, Florida, Louisiana, Michigan, Pennsylvania, and Texas) from 10 April 2017 until 12 March 2018. We enrolled both symptomatic and asymptomatic participants. Participant inclusion criteria were (1) attending a study clinic for evaluation of STI(s); (2) ≥18 years of age at date of testing; (3) able and willing to provide informed consent; and (4) willing to comply with study procedures. We excluded participants for receipt of any systemic antibacterial drug in the past 14 days and/or receipt of myelosuppressive chemotherapy in the past 30 days. Participants were not required to have specific behaviors or symptoms to join the study. We obtained human subjects research approval from all necessary institutional review boards (IRBs). Participants provided oral informed consent. Due to the minimal risk of study participation, the IRBs waived the requirement for written documentation of consent.

### Study Procedures

Using each manufacturer’s urogenital specimen collection kit containing a swab and transport media, trained clinicians collected 4 swabs from the participant’s pharynx and 4 swabs from the rectum, in addition to any swabs taken as part of routine clinical care. Clinicians collected swabs for routine care first. We randomized the order of collection of the research swabs to account for the possibility that the performance of each assay may be affected by order of specimen collection. Swabs were stored and transported per manufacturer guidelines for urogenital collection and testing, and specimen collection training was standardized for all study sites [19–23]. While participants consented to both pharyngeal and rectal swab collection, if swabs from only 1 anatomic site were submitted, the swabs for that anatomic site were included in the analysis. If clinicians submitted <4 swabs at a site, we excluded results from that participant’s anatomic site from the analysis.

### Assays Under Consideration

The study evaluated 3 distinct NAATs, each of which utilizes different molecular methods, microbial genetic targets specific to NG and CT only, and instruments (Table 1). Henceforth, the 3 assays under interest will be referred to as assays 1, 2, and 3. A fourth NAAT with unique molecular genetic targets was used in case of disagreement between the 2 comparator assays and is referred to as the tiebreaker assay. The tiebreaker assay tested for CT or NG separately.

**Table 1.** Assay Names, Molecular Methods and Targets, and Laboratory Testing Platforms

Assay	Manufacturer	Target(s)	Laboratory Test Platform
Assay 1	Xpert CT/NG Assay (Cepheid, Sunnyvale, California)	Real-time PCR to detect 2 noncontiguous chromosomal DNA regions from *Neisseria gonorrhoeae* (NG2 and NG4)—both of which must be positive to yield a positive result—and 1 chromosomal DNA target from *Chlamydia trachomatis* (CT1) [20]	GeneXpert System
Assay 2	Aptima Combo 2 Assay (Hologic, Inc, Marlborough, Massachusetts)	Utilizes target capture, TMA, and a dual kinetic assay to detect regions from the 16S rRNA of NG and the 23S rRNA from CT using labeled DNA probes [23]	Panther System
Assay 3	Abbott RealTime CT/GC assay (Abbott Laboratories, Abbott Park, Illinois)	A combination assay that uses real-time PCR to detect a highly conserved region within the *Opa* gene of NG and 2 distinct regions within the CT cryptic plasmid DNA [19]	Abbott m2000 RealTime System
NG tiebreaker	Aptima NG assay (Hologic, Inc)	Utilizes target capture, TMA, and hybridization protection assays to identify the presence of NG 16S rRNA^a^ [20]	Tigris DTS System
CT tiebreaker	Aptima CT assay (Hologic, Inc)	Utilize target capture, TMA, and hybridization protection assays to identify the presence of CT 16S rRNA^a^ [21]	Tigris DTS System

Abbreviations: CT, *Chlamydia trachomatis*; DTS, direct tube sampling; GC/NG, *Neisseria gonorrhoeae*; PCR, polymerase chain reaction; rRNA, ribosomal RNA; TMA, transcription-mediated amplification.

^a^Both tiebreaker assays utilize distinct molecular genetic targets from the assays under evaluation.

### Central Laboratory Procedures

Two reference laboratories shared the processing and testing of the assays for the study. Laboratories conducted quality control and quality assurance procedures according to the manufacturers’ recommendations and in compliance with the College of American Pathology. Each clinical study site sent specimens to 1 of the 2 laboratories. Trained laboratory staff processed swabs, tested the specimens, and interpreted the results according to each manufacturer’s instructions on respective FDA-cleared urogenital assays [19–23]. Staff repeated initial equivocal, invalid, or otherwise undetermined results once per manufacturer guidelines before classifying as positive, negative, equivocal, or no result.

One reference testing laboratory was responsible for running the tiebreaker assay, regardless of which laboratory ran the initial specimen. Laboratory testing staff were blind to clinical information and the composite reference results.

### Clinical Data

Site personnel included assessed participants for clinical signs and symptoms of infection in the pharynx and/or rectum as well as age, race, ethnicity, sex, and gender. Staff collected pharyngeal symptoms including sore throat, pain with swallowing, and swollen or tender lymph nodes. Staff collected rectal symptoms including rectal discharge, rectal bleeding, rectal itching, or painful bowel movement. Any other symptoms at either anatomic site were recorded as free text. Study staff recorded reason for withdrawal from the study for subjects who withdrew consent.

### Determination of the Anatomic Site Infected Status Composite Reference Method

**Figure 1.**
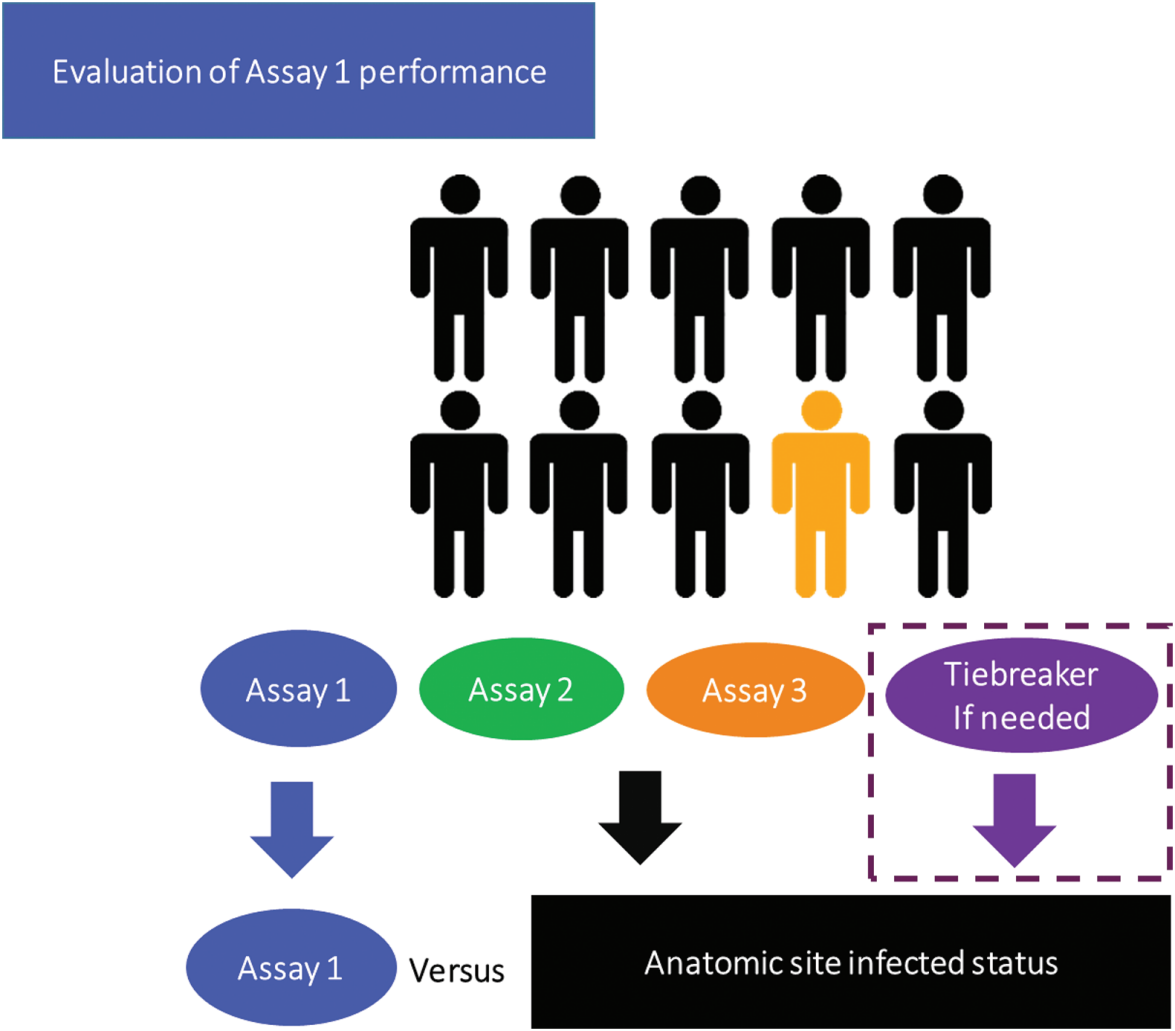
Schematic of the Master Protocol concept from the prototype perspective of evaluation of diagnostic performance of assay 1. The results from assays 2 and 3 determine the anatomic site infected status for assay 1. Tiebreaker tests are performed only if indicated. Evaluation of assay 2 and 3 would proceed utilizing a similar approach. Individuals depicted in black and yellow represent participants without and with infection, respectively.

Based on the prior methods for creating a composite reference in situations where either no accepted gold standard exists or when a gold standard such as bacterial culture is less sensitive than the assay under evaluation (ie, patient infected status), the anatomic site infected status (ASIS) was determined for each anatomic site and each organism (Figure 1; Supplementary Table 1) [15, 24]. Possible outcomes of the ASIS included infected, not infected, indeterminate, and invalid/excluded from analysis. For each assay under consideration, the ASIS was derived from the 2 other assay results. We defined ASIS outcomes a priori in the protocol. We considered the anatomic site infected if both of the other assay results were positive. We considered the anatomic site not infected when both other assay results were negative. If there was discordance between the comparator assays, we performed the tiebreaker assay. In that case, agreement of 2 of 3 of the comparator assays determined the ASIS. As the tiebreaker assay was a single CT or NG assay, the tiebreaker assay was only run for the organism with disagreement (eg, if the comparator NG results were discordant and CT concordant, the tiebreaker assay was only run for NG). In the rare case when 2 of the 3 assays were equivocal or 1 was equivocal and 1 not performed, 1 assay result alone was used to determine the ASIS. If 2 assays were not performed, then no ASIS could be determined (ie, invalid) and results from that subject’s anatomic site were excluded from the analysis.

### Determination of Positivity

After reviewing the results, we used the ASIS determined in the evaluation of assay 3, which used the results from assays 1 and 2 (and if necessary the tiebreaker assay), to estimate positivity. That ASIS was chosen to define positivity as assays 1 and 2 performed better than assay 3. We calculated percent positivity as the number of ASIS results classified as infected divided by the sum of all ASIS results for each anatomic site.

### Statistical Methods

For each combination of assay, anatomic site, and organism, positive percent agreement (PPA) and negative percent agreement were estimated using standard epidemiological methods, with 95% score confidence intervals (CIs) [25–27]. The composite reference standard was the ASIS, described above. The study was designed with a sample size of 2500 evaluable participants under the assumption that prevalence of NG infection in the rectum, NG infection in the pharynx, and CT in the rectum would each be greater than 7.5% in the population under evaluation [28–35]. That value provided at least an 80% probability of obtaining at least 174 participants with infection at each of those 3 anatomic sites. In turn, that sample size would offer at least 85% power to evaluate whether 90% PPA could be ruled out by the lower bound of the 95% CI if the true PPA of the assay was 96%. An independent reviewer reviewed the observed positivity by anatomic site every 500 participants during the course of the study. Based on the third review, the planned sample size was increased to 2600 evaluable subjects.

Positive and negative predictive values (PPVs and NPVs, respectively) and positive and negative likelihood ratios (LRs) were also calculated. We calculated 95% CIs for the PPVs and NPVs using adjusted logit transformation, whereas the log method was used for the positive and negative LRs. We plotted PPVs and NPVs as a function of prevalence [26, 36]. Categorical tests for association were conducted using a χ ^2^ test. Sensitivity analyses classifying indeterminate ASIS results on the basis of reported symptom status or categorizing all indeterminate ASIS results as infected or as not infected were also examined. Authors L. K., T. T. T. T., and S. R. E. performed the statistical analyses.

## RESULTS

**Figure 2.**
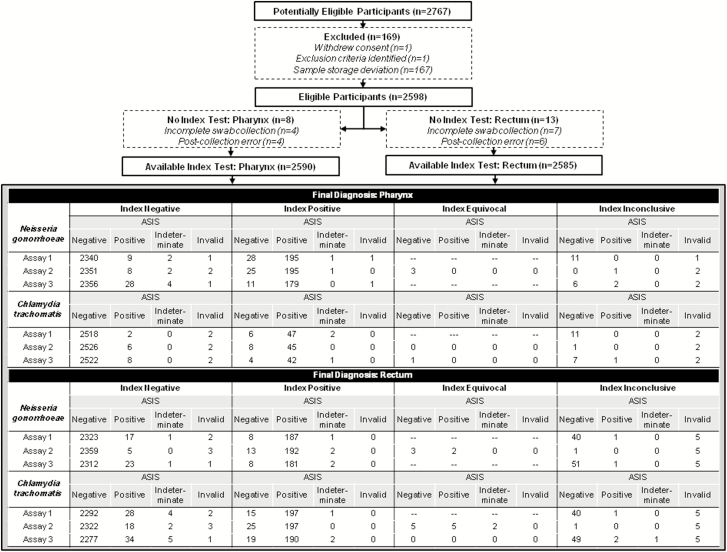
Standards for Reporting of Diagnostic Accuracy Studies diagram of participant flow. Abbreviation: ASIS, anatomic site infected status.

The final study population included 2598 participants out of 2767 enrolled (Figure 2). Reasons for exclusion of enrolled participants included a protocol deviation resulting in samples stored outside of the appropriate temperature range (n = 167) at 1 study site, withdrawal of consent (n = 1), and postenrollment exclusion (n = 1). Of the 2598 enrolled eligible participants, there were 2590 (99.7%) with evaluable pharyngeal specimens and 2585 (99.5%) with evaluable rectal specimens. Reasons for unevaluable participant specimens included <4 swabs submitted, error in collection method, or shipment error. Swabs were not collected in randomized order in 22 participants (<1%), all of whom were included in the study analysis.

Table 2 shows the demographic characteristics and presenting symptom status by anatomic site among participants. The majority of participants (79%) were male and most were asymptomatic (88% at the pharyngeal site, 92% at the rectal site). *Neisseria gonorrhoeae* positivity was 8.1% in the pharynx and 7.9% in the rectum, whereas positivity for CT was 2.0% in the pharynx and 8.7% in the rectum. Positivity varied by study clinic and organism, with ranges at the 9 clinics of 0.9%–16.9% for NG and 0–4.6% for CT in the pharynx, and 0.9%–17.1% for NG and 1.8%–14.3% for CT in the rectum.

**Table 2.** Participant Demographics and Disease Prevalence (N = 2598)

Demographics	No. (%)
Sex at birth	
Male	2059 (79)
Female	539 (21)
Gender	
Man	2010 (77)
Woman	532 (20)
Transgender man	3 (0.1)
Transgender woman	42 (2)
Genderqueer	8 (0.3)
Declined to answer	3 (0.1)
Age, y, median (IQR)	30 (25–41)
Race	
White	1285 (49)
Black	935 (36)
Asian	84 (3)
Other race	145 (6)
>1 race	71 (3)
Unknown/declined to answer	78 (3)
Ethnicity	
Hispanic or Latino	772 (30)
Not Hispanic or Latino	1814 (70)
Unknown/declined to answer	12 (0.5)
Site of enrollment	
A	399 (15)
B	367 (14)
C	367 (14)
D	356 (14)
E	337 (13)
F	290 (11)
G	227 (9)
H	143 (6)
I	112 (4)
Any pharyngeal symptoms	307 (12)
Any rectal symptoms	198 (8)

Data are presented as no. (%) unless otherwise indicated.

Abbreviation: IQR, interquartile range.

**Figure 3.**
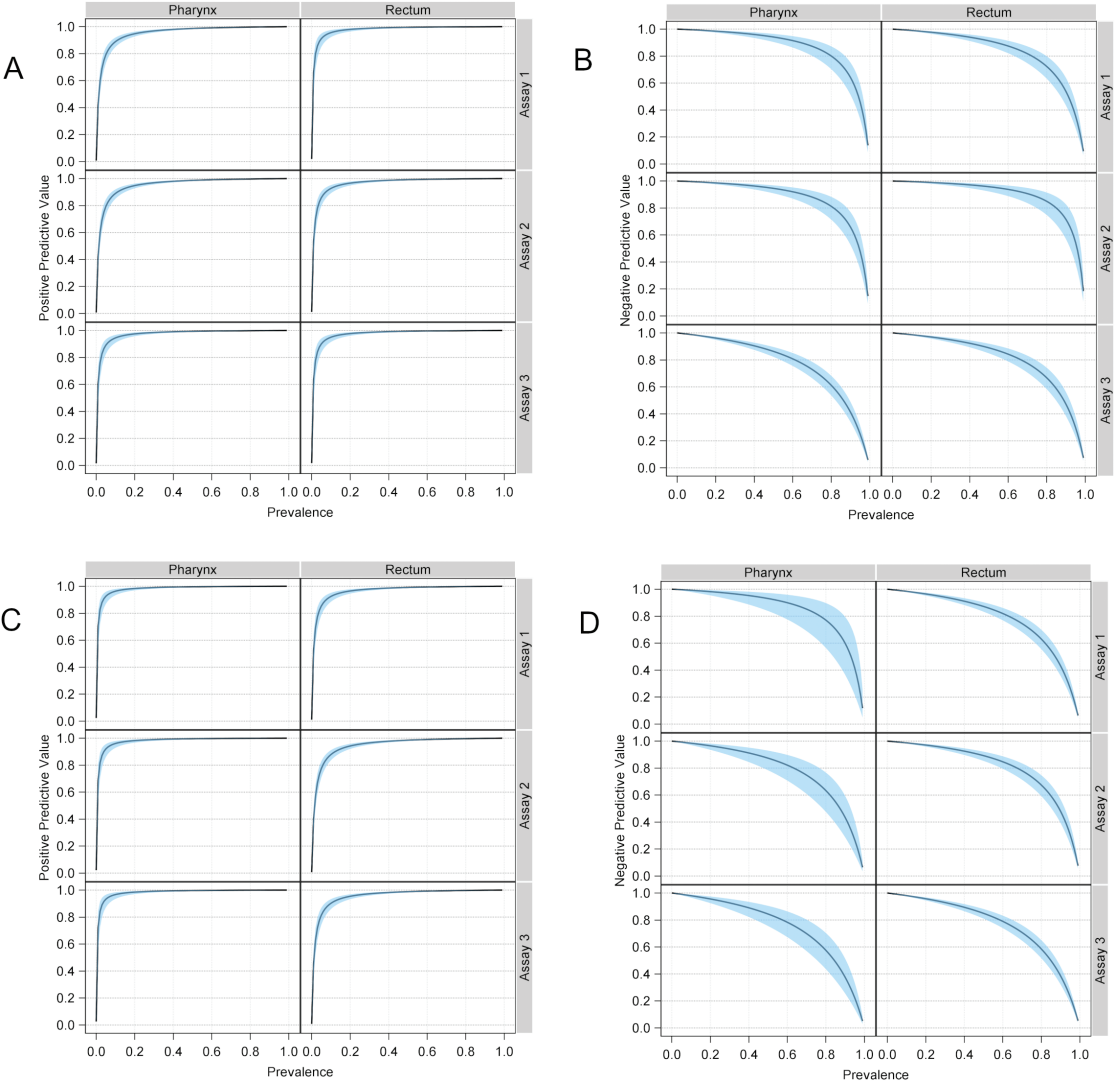
*A*, Positive predictive value (PPV) as a function of *Neisseria gonorrhoeae* prevalence. *B*, Negative predictive value (NPV) as a function of *N. gonorrhoeae* prevalence. *C*, PPV as a function of *Chlamydia trachomatis* prevalence. *D*, NPV as a function of *C. trachomatis* prevalence. All blue bands reflect 95% confidence intervals.

Table 3 shows the diagnostic performance measures for the 3 assays by anatomic site. Sensitivity analyses, classifying indeterminate results based on symptom status or as either all infected or all uninfected, yielded similar results (data not shown). Tiebreaker testing was performed for ASIS determination in 96 (4%) participants for pharyngeal NG, 146 (6%) for rectal NG, 44 (2%) for pharyngeal CT, and 186 (7%) for rectal CT. There were no significant associations between swab order and assay performance result for either organism or anatomic site (NG pharynx, *P* = .65; NG rectum, *P* = .95; CT pharynx, *P* = .34; CT rectum, *P* = .80). Figure 3 shows the PPVs and NPVs as a function of infection prevalence by assay.

**Table 3.** Performance of Assays Under Consideration for Detection of Pharyngeal and Rectal *Neisseria gonorrhoeae* and *Chlamydia trachomatis*

Site and Infection	PPA (95% CI)	NPA (95% CI)	PPV^a^ (95% CI)	NPV^a^ (95% CI)	Positive LR (95% CI)	Negative LR (95% CI)
Pharynx, NG						
Assay 1	94.7 (90.7–97.0)	98.8 (98.2–99.1)	87.1 (82.0–90.8)	99.5 (99.2–99.7)	77 (54–111)	0.05 (.03–.10)
Assay 2	95.1 (91.3–97.3)	98.8 (98.3–99.2)	88.2 (83.3–91.8)	99.6 (99.2–99.8)	78 (54–112)	0.05 (.03–.09)
Assay 3	84.8 (79.4–89.0)	99.5 (99.2–99.7)	94.2 (89.9–96.7)	98.7 (98.1–99.0)	183 (101–330)	0.15 (.11–.21)
Rectum, NG						
Assay 1	91.2 (86.5–94.4)	99.6 (99.3–99.8)	95.4 (91.5–97.6)	99.3 (98.8–99.5)	238 (124–457)	0.08 (.05–.15)
Assay 2	96.5 (92.9–98.3)	99.2 (98.8–99.5)	92.8 (88.4–95.6)	99.8 (99.5–99.9)	127 (80–202)	0.04 (.02–.07)
Assay 3	88.3 (83.2–92.0)	99.6 (99.2–99.8)	94.8 (90.6–97.1)	99.0 (98.5–99.3)	205 (110–381)	0.12 (.08–.17)
Pharynx, CT						
Assay 1	95.9 (86.3–98.9)	99.7 (99.4–99.8)	85.5 (73.8–92.4)	99.9 (99.7–100.0)	303 (151–606)	0.04 (.01–.16)
Assay 2	88.2 (76.6–94.5)	99.7 (99.4–99.8)	84.9 (72.9–92.1)	99.8 (99.5–99.9)	279 (139–562)	0.12 (.06–.25)
Assay 3	84.0 (71.5–91.7)	99.8 (99.5–99.9)	89.4 (77.4–95.4)	99.7 (99.4–99.8)	354 (158–794)	0.16 (.08–.30)
Rectum, CT						
Assay 1	86.0 (80.9–89.9)	99.3 (98.9–99.6)	92.5 (88.1–95.3)	98.6 (98.1–99.0)	124 (76–203)	0.14 (.10–.19)
Assay 2	88.7 (83.9–92.3)	98.7 (98.2–99.1)	88.7 (83.9–92.3)	99.1 (98.7–99.4)	70 (49–100)	0.11 (.08–.17)
Assay 3	83.0 (77.5–87.2)	99.1 (98.6–99.4)	90.0 (85.3–93.4)	98.3 (97.7–98.8)	91 (59–140)	0.17 (.13–.23)

Abbreviations: CI, confidence interval; CT, *Chlamydia trachomatis*; LR, likelihood ratio; NG, *Neisseria gonorrhoeae*; NPA, negative percent agreement; NPV, negative predictive value; PPA, positive percent agreement; PPV, positive predictive value.

^a^PPVs and NPVs were calculated based on the positivity observed in this study.

### Subgroup Analysis

Positivity was higher among symptomatic participants (NG: pharynx, 13%, rectum, 20%; CT: pharynx, 2.9%, rectum, 13%) compared to asymptomatic participants (NG: pharynx, 7.4%, rectum, 6.9%; CT: pharynx, 1.8%, rectum, 8.4%). Positivity for NG in the pharynx and rectum for males was 9.9% and 9.8%, respectively. For females, positivity for NG in the pharynx was 0.9% and in the rectum was 0.7%. Male CT positivity was 2.2% in the pharynx and 9.4% in the rectum, while female CT positivity was 0.9% and 6.4% in the pharynx and rectum, respectively.

Supplementary Tables 2 and 3 show the performance of the assays by subgroup. Sensitivity analyses for each subgroup, classifying indeterminate results based on symptom status or as either all infected or all uninfected, yielded similar results by both sex and symptom status (data not shown).

### Swab Collection Complications

There were a total of 21 (<1%) participants with swab collection complications. The majority of those were related to a problem with the specimen handling or testing materials, such as placing a swab in the wrong tube, dropping the swab, or inability to snap the swab at the scored handle. One participant reported excessive discomfort during rectal swab collection and withdrew consent for study involvement.

## DISCUSSION

Using a Master Protocol to evaluate multiple assays in a single study population, we generated diagnostic performance data for 3 assays at 2 anatomic sites, which supported regulatory submission for 2 of the manufacturers. The FDA cleared the Aptima Combo 2 Assay and the Xpert CT/NG assay for marketing on 23 May 2019, an important step for public health that is expected to expand testing reach, thereby improving control of these infections and their attendant complications [37]. A unique strength of this study included collaboration between device manufacturers, the National Institute of Allergy and Infectious Diseases of the National Institutes of Health, the Antibacterial Resistance Leadership Group, and the FDA to develop a composite reference method to use as a comparator. That composite reference method allowed for the efficient evaluation of multiple assays with higher analytic sensitivity than the traditional culture-based standard [28–30, 35, 38]. This feasible and efficient Master Protocol study design can and should be replicated for the evaluation of other infectious diseases diagnostics [18]. Given the rapidly developing and widespread problems with antimicrobial resistance in NG, future studies may include testing of diagnostics that can rapidly identify antimicrobial-resistant strains, allowing for more appropriate and targeted treatment [3, 39, 40].

We observed very-good to excellent PPA when compared to the composite reference for the detection of both pharyngeal and rectal NG and CT. It should be noted that assay 3—which is the 1 assay for which FDA clearance was not sought—had consistently lower PPA compared to the other 2 assays, which may be relevant in light of the fact that many testing laboratories may use this test under the Clinical Laboratory Improvement Amendments. The 2 FDA-cleared assays had similar performance characteristics for detection of the 3 anatomic site-organism combinations for which this study was powered. Negative percent agreement was excellent and similar across assays. Our results align with prior published estimates of the performance for the 3 assays evaluated in this study. For the detection of NG, prior reported sensitivities were 84%–100% (pharyngeal) and 76%–100% (rectal), and specificities were 96%–100% (pharyngeal) and 95%–100% (rectal) [29–31, 34, 35, 41]. For the detection of CT, prior reported sensitivities were 100% for the pharyngeal site and 71%–100% for the rectal site, and specificities were >99% for the pharynx and 89%–100% for the rectum [28–33, 42]. Differences are likely due to variation in the definition of the reference method used in each study. In the study population of participants presenting for STI testing at a wide variety of clinics, the positivity of infection was similar to other estimates from a variety of settings. Prior investigators have reported median prevalence values of 8% for NG in the pharynx, 10% for NG in the rectum, 3% for CT in the pharynx, and 14% for CT in the rectum in persons seeking extragenital testing [43–45].

The frequency of NG and CT infection in asymptomatic patients was high, underscoring the importance of further research to understand the implications of these infections and how they impact population-level transmission, as well as to determine the benefit of routine screening and treatment of asymptomatic infection at these sites. Notably, the rates of CT in the rectum were high in females, which may reflect concomitant urogenital (ie, cervical) infection but has also been described in a substantial proportion of those without urogenital infection. We did not have access to urogenital testing results that might have been done as part of routine clinical care. Additional cost-effectiveness studies of routine rectal CT screening in females are warranted [46–48].

Several limitations of our study should be noted. First, in the absence of a universally accepted gold-standard comparator, a new comparator (the ASIS) was developed [15, 49]. Since all of the assays under consideration were NAATs, testing errors could be correlated resulting in biased estimates. However, each assay utilized different molecular methods and genetic targets of each organism, which should mitigate that issue [12–14]. In addition, because the ASIS composite reference method incorporated results from the other 2 comparator assays, limitations in performance of one of the assays had the potential to affect estimates from multiple assays. Finally, this study did not evaluate self-collection methods, which are preferred in many settings and should be evaluated in future studies in real-world settings, such as clinics and laboratory specimen collection sites.

In summary, this trial allowed evaluation of multiple new diagnostic assays in a single study to develop clinical performance data, which ultimately led to FDA clearances of 2 assays.

## Supplementary Data

Supplementary materials are available at *Clinical Infectious Diseases* online. Consisting of data provided by the authors to benefit the reader, the posted materials are not copyedited and are the sole responsibility of the authors, so questions or comments should be addressed to the corresponding author.

ciz1105_suppl_Supplementary_Material

ciz1105_suppl_Supplementary_data

## Notes

***Acknowledgments.*** The authors are grateful to Sixto Pacheco of BioCollections Worldwide, Inc, for his leadership of 1 of the 2 central laboratories; and to Dr Susan Rosenkranz for her independent statistical review.

***Disclaimer.*** The content is solely the responsibility of the authors and does not necessarily represent the official views of the Antibacterial Resistance Leadership Group or the National Institutes of Health (NIH). Cepheid and Hologic, Inc, provided testing supplies for the research described herein but neither company was involved with clinical site monitoring or execution of the data analyses. C. D. D. of the National Institute of Allergy and Infectious Diseases (NIAID) at the NIH was involved in study conceptualization and design, and members of the NIAID team participated in team meetings and communication.

***Financial support.*** This work was supported in part by the NIAID/NIH (award number UM1AI104681). V. G. F. was supported by a Mid-Career Mentoring Award (number 2K24-AI093969) from the NIH.

***Potential conflicts of interest.*** M. P. reports payment from Duke University to act as a central laboratory during the conduct of the study. L. K. reports grants from the Division of Microbiology and Infectious Diseases. R. P. has received grants from CD Diagnostics, BioFire, Curetis, Merck, Hutchison Biofilm Medical Solutions, Accelerate Diagnostics, Allergan, and The Medicines Co; is or has been a consultant to Curetis, Qvella, St Jude, Beckman Coulter, Morgan Stanley, Heraeus Medical GmbH, CORMATRIX, Specific Technologies, Diaxonit, Selux Dx, GenMark Diagnostics, LBT Innovations Ltd, PathoQuest, and Genentech (monies are paid to Mayo Clinic); has a patent on *Bordetella pertussis/parapertussis* polymerase chain reaction assay issued, a patent on a device/method for sonication with royalties paid by Samsung to Mayo Clinic issued, and a patent on an antibiofilm substance issued; and has served on an Actelion data monitoring board. R. P. has received travel reimbursement from the American Society for Microbiology (ASM) and the Infectious Diseases Society of America (IDSA), an editor’s stipend from ASM and IDSA, and honoraria from the National Board of Medical Examiners, UpToDate, and the Infectious Diseases Board Review Course. S. D. has received consulting fees from Genentech and Basilea, outside the submitted work. S. R. E. has received grants from NIAID/NIH, during the conduct of the study; and has received personal fees from Takeda/Millennium, Pfizer, Roche, Novartis, Achaogen, the Huntington Study Group, ACTTION, Genentech, Amgen, GSK, American Statistical Association, US Food and Drug Administration, Osaka University, National Cerebral and Cardiovascular Center of Japan, NIH, Society for Clinical Trials, Statistical Communications in Infectious Diseases (DeGruyter), AstraZeneca, Teva, Austrian Breast and Colorectal Cancer Study Group/Breast International Group, Alliance Foundation Trials, Zeiss, Dexcom, ASM, Taylor and Francis, Claret Medical, Vir, Arrevus, Five Prime, Shire, Alexion, Gilead, Spark, Clinical Trials Transformation Initiative, Nuvelution, Tracon, Deming Conference, Antimicrobial Resistance and Stewardship Conference, World Antimicrobial Congress, WAVE, Advantagene, Braeburn, Cardinal Health, Lipocine, Microbiotix, and Stryker, outside the submitted work. V. G. F. has served on the advisory board for Merck; has received consulting fees from Pfizer, Novartis, Galderma, Novadigm, Durata, Debiopharm, Genentech, Achaogen, Affinium, The Medicines Co, Cerexa, Tetraphase, Trius, MedImmune, Bayer, Theravance, Cubist, Basilea, Affinergy, Janssen, xBiotech, Contrafect, Regeneron, Basilea, and Destiny; has received grants from NIH, MedImmune, Cerexa/Forest/Actavis/Allergan, Pfizer, Advanced Liquid Logics, Theravance, Novartis, Cubist/Merck, Medical Biosurfaces, Locus, Affinergy, Contrafect, Karius, Genentech, Regeneron, and Basilea; has received education fees from Green Cross, Cubist, Cerexa, Durata, Theravance, and Debiopharm; has received royalties from UpToDate; and holds a pending sepsis diagnosis patent, all outside the submitted work. All other authors report no potential conflicts of interest. All authors have submitted the ICMJE Form for Disclosure of Potential Conflicts of Interest. Conflicts that the editors consider relevant to the content of the manuscript have been disclosed.
