# Supplementary material for: Simultaneous Evaluation of Diagnostic Assays for Pharyngeal and Rectal Neisseria gonorrhoeae and Chlamydia trachomatis Using a Master Protocol
Source: Clin Infect Dis. 2019 Nov 17;71(9):2314–22. doi: 10.1093/cid/ciz1105 (PMC7713680; doi:10.1093/cid/ciz1105)
Supplement: ciz1105_suppl_Supplementary_Material [file ciz1105_suppl_supplementary_material.docx]

Table of Contents

[**Supplementary Table 1.** Determination of the anatomic site infected status (ASIS). 2](#_Toc12355016)

[**Supplementary Table 2a.** Performance of assays under consideration for detection of pharyngeal and rectal *Neisseria gonorrhoeae* and *Chlamydia trachomatis* for participants with symptoms at the site of testing. 4](#_Toc12355017)

[**Supplementary Table 2b.** Performance of assays under consideration for detection of pharyngeal and rectal *Neisseria gonorrhoeae* and *Chlamydia trachomatis* for participants without symptoms at the site of testing. 5](#_Toc12355018)

[**Supplementary Table 3a.** Performance of the assays under consideration for detection of pharyngeal and rectal *Neisseria gonorrheae* and *Chlamydia trachomatis* for male participants. 6](#_Toc12355019)

[**Supplementary Table 3b.** Performance of the assays under consideration for detection of pharyngeal and rectal *Neisseria gonorrheae* and *Chlamydia trachomatis* for female participants. 7](#_Toc12355020)

**Supplementary Table 1.** Determination of the anatomic site infected status (ASIS).

| **Reference assay 1 result** | **Reference assay 2 result** | **Tiebreaker assay result** | **Anatomic Site Infection Status** |
| --- | --- | --- | --- |
| + | + | Not indicated | Infected |
| + | - | + | Infected |
| + | Equivocal | + | Infected |
| + | No result* | + | Infected |
| + | - | - | Not infected |
| + | - | Equivocal | Indeterminate |
| + | - | No result | Indeterminate |
| + | Equivocal | - | Indeterminate |
| + | Equivocal | Equivocal | Infected |
| + | Equivocal | No result | Infected |
| + | No result | - | Indeterminate |
| + | No result | Equivocal | Infected |
| + | No result | No result | Invalid, remove from analysis |
| - | - | Not indicated | Not infected |
| - | + | - | Not infected |
| - | Equivocal | - | Not infected |
| - | No result | - | Not infected |
| - | + | + | Infected |
| - | + | Equivocal | Indeterminate |
| - | + | No result | Indeterminate |
| - | Equivocal | + | Indeterminate |
| - | Equivocal | Equivocal | Not infected |
| - | Equivocal | No result | Not infected |
| - | No result | + | Indeterminate |
| - | No result | Equivocal | Not infected |
| - | No result | No result | Invalid, remove from analysis |
| Equivocal | + | + | Infected |
| Equivocal | - | - | Not infected |
| Equivocal | + | - | Indeterminate |
| Equivocal | + | Equivocal | Infected |
| Equivocal | + | No result | Infected |
| Equivocal | - | + | Indeterminate |
| Equivocal | - | Equivocal | Not infected |
| Equivocal | - | No result | Not infected |
| Equivocal | No result | + | Infected |
| Equivocal | No result | - | Not infected |
| Equivocal | No result | Equivocal | Indeterminate |
| Equivocal | No result | No result | Invalid, remove from analysis |
| No result | + | + | Infected |
| No result | - | - | Not infected |
| No result | No result | Not indicated | Invalid, remove from analysis |
| No result | No result | Not indicated | Invalid, remove from analysis |
| No result | + | - | Indeterminate |
| No result | + | Equivocal | Infected |
| No result | + | No result | Invalid, remove from analysis |
| No result | - | + | Indeterminate |
| No result | - | Equivocal | Not infected |
| No result | - | No result | Invalid, remove from analysis |
| No result | Equivocal | + | Infected |
| No result | Equivocal | - | Not infected |
| No result | Equivocal | Equivocal | Indeterminate |
| No result | Equivocal | No result | Invalid, remove from analysis |

*This can occur either because the assay result was invalid or because the assay could not be run (e.g., too little sample, improperly shipped, no sample received).

# **Supplementary Table 2a.** Performance of assays under consideration for detection of pharyngeal and rectal *Neisseria gonorrhoeae* and *Chlamydia trachomatis* for participants with symptoms at the site of testing.

|  | **Positive percent agreement (95% CI)** | **Negative percent agreement (95% CI)** | **Positive predictive value* (95% CI)** | **Negative predictive value (95% CI)** | **Positive likelihood ratio (95% CI)** | **Negative likelihood ratio (95% CI)** |  |
| --- | --- | --- | --- | --- | --- | --- | --- |
| **Neisseria gonorrhoeae** | | | | | | | |
| **Pharynx** (N = 306) |  |  |  |  |  |  |  |
| Assay 1 | 92.9 (81.0, 97.5) | 98.9 (96.7, 99.6) | 92.9 (81.0, 97.5) | 98.9 (96.7, 99.6) | 82 (26, 253) | 0.07 (0.02, 0.21) |  |
| Assay 2 | 97.5 (87.1, 99.6) | 98.5 (96.2, 99.4) | 95.1 (83.9, 98.7) | 99.6 (97.9, 99.9) | 65 (24, 172) | 0.03 (0.00, 0.18) |  |
| Assay 3 | 86.0 (72.7, 93.4) | 99.6 (97.9, 99.9) | 97.4 (86.5, 99.5) | 97.8 (95.2, 99.0) | 226 (32, 1606) | 0.14 (0.07, 0.29) |  |
| **Rectum** (N = 195) |  |  |  |  |  |  |  |
| Assay 1 | 97.4 (86.8, 99.5) | 100.0 (97.5, 100.0) | 100.0 (90.8, 100.0) | 99.3 (96.3, 99.9) | Not evaluable | Not evaluable |  |
| Assay 2 | 97.4 (86.8, 99.5) | 100.0 (97.6, 100.0) | 100.0 (90.8, 100.0) | 100.0 (97.6, 100.0) | Not evaluable | Not evaluable |  |
| Assay 3 | 94.9 (83.1, 98.6) | 100.0 (97.6, 100.0) | 100.0 (90.6, 100.0) | 98.7 (95.4, 99.6) | Not evaluable | Not evaluable |  |
| **Chlamydia trachomatis** | | | | | | | |
| **Pharynx** (N = 306) |  |  |  |  |  |  |  |
| Assay 1 | 100.0 (70.1, 100.0) | 100.0 (98.7, 100.0) | 100.0 (70.1, 100.0) | 100.0 (98.7, 100.0) | Not evaluable | Not evaluable |  |
| Assay 2 | 100.0 (70.1, 100.0) | 99.7 (98.1, 99.9) | 90.0 (59.6, 98.2) | 100.0 (98.7, 100.0) | Not evaluable | Not evaluable |  |
| Assay 3 | 88.9 (56.5, 98.0) | 100.0 (98.7, 100.0) | 100.0 (67.6, 100.0) | 99.7 (98.1, 99.9) | Not evaluable | Not evaluable |  |
| **Rectum** (N = 195) |  |  |  |  |  |  |  |
| Assay 1 | 81.5 (63.3, 91.8) | 98.8 (95.6, 99.7) | 91.7 (74.2, 97.7) | 97.0 (93.1, 98.7) | 66 (16, 263) | 0.19 (0.08, 0.41) |  |
| Assay 2 | 92.0 (75.0, 97.8) | 98.2 (94.9, 99.4) | 92.0 (75.0, 97.8) | 98.8 (95.7, 99.7) | 51 (17, 158) | 0.08 (0.02, 0.31) |  |
| Assay 3 | 84.6 (66.5, 93.8) | 98.8 (95.7, 99.7) | 91.7 (74.2, 97.7) | 97.6 (94.0, 99.1) | 70 (18, 281) | 0.16 (0.06, 0.38) |  |

*Subgroup-specific positive and negative predictive values were calculated based on the positivity observed for the subgroup.

# **Supplementary Table 2b.** Performance of assays under consideration for detection of pharyngeal and rectal *Neisseria gonorrhoeae* and *Chlamydia trachomatis* for participants without symptoms at the site of testing.

|  | **Positive percent agreement (95% CI)** | **Negative percent agreement (95% CI)** | **Positive predictive value* (95% CI)** | **Negative predictive value (95% CI)** | **Positive likelihood ratio (95% CI)** | **Negative likelihood ratio (95% CI)** |  |
| --- | --- | --- | --- | --- | --- | --- | --- |
| **Neisseria gonorrhoeae** | | | | | | | |
| **Pharynx** (N = 2284) |  |  |  |  |  |  |  |
| Assay 1 | 95.1 (90.7, 97.5) | 98.8 (98.2, 99.2) | 85.7 (79.9, 90.1) | 99.6 (99.2, 99.8) | 77 (52, 113) | 0.05 (0.03, 0.10) |  |
| Assay 2 | 94.5 (90.0, 97.1) | 98.8 (98.3, 99.2) | 86.7 (80.9, 90.9) | 99.6 (99.2, 99.8) | 80 (54, 118) | 0.06 (0.03, 0.10) |  |
| Assay 3 | 84.5 (78.3, 89.2) | 99.5 (99.1, 99.7) | 93.4 (88.3, 96.4) | 98.8 (98.2, 99.2) | 178 (96, 331) | 0.16 (0.11, 0.22) |  |
| **Rectum** (N = 2390) |  |  |  |  |  |  |  |
| Assay 1 | 89.8 (84.2, 93.5) | 99.6 (99.2, 99.8) | 94.3 (89.5, 97.0) | 99.2 (98.8, 99.5) | 218 (113, 419) | 0.10 (0.07, 0.16) |  |
| Assay 2 | 96.3 (92.1, 98.3) | 99.2 (98.7, 99.5) | 91.1 (85.9, 94.5) | 99.8 (99.5, 99.9) | 119 (75, 189) | 0.04 (0.02, 0.08) |  |
| Assay 3 | 86.7 (80.7, 91.1) | 99.5 (99.2, 99.7) | 93.5 (88.5, 96.4) | 99.0 (98.5, 99.3) | 188 (101, 350) | 0.13 (0.09, 0.20) |  |
| **Chlamydia trachomatis** | | | | | | | |
| **Pharynx** (N = 2284) |  |  |  |  |  |  |  |
| Assay 1 | 95.0 (83.5, 98.6) | 99.6 (99.3, 99.8) | 82.6 (69.3, 90.9) | 99.9 (99.7, 100.0) | 265 (132, 531) | 0.05 (0.01, 0.19) |  |
| Assay 2 | 85.7 (72.2, 93.3) | 99.7 (99.4, 99.8) | 83.7 (70.0, 91.9) | 99.7 (99.4, 99.9) | 274 (129, 580) | 0.14 (0.07, 0.30) |  |
| Assay 3 | 82.9 (68.7, 91.5) | 99.7 (99.4, 99.9) | 87.2 (73.3, 94.4) | 99.7 (99.4, 99.8) | 308 (137, 694) | 0.17 (0.09, 0.34) |  |
| **Rectum** (N = 2390) |  |  |  |  |  |  |  |
| Assay 1 | 86.6 (81.3, 90.6) | 99.3 (98.9, 99.6) | 92.6 (88.0, 95.5) | 98.8 (98.2, 99.1) | 133 (79, 225) | 0.13 (0.09, 0.19) |  |
| Assay 2 | 88.3 (83.1, 92.1) | 98.8 (98.2, 99.1) | 88.3 (83.1, 92.1) | 99.2 (98.7, 99.5) | 71 (49, 104) | 0.12 (0.08, 0.17) |  |
| Assay 3 | 82.8 (77.0, 87.3) | 99.1 (98.6, 99.4) | 89.8 (84.7, 93.4) | 98.4 (97.7, 98.8) | 93 (59, 146) | 0.17 (0.13, 0.24) |  |

*Subgroup-specific positive and negative predictive values were calculated based on the positivity observed for the subgroup.

# **Supplementary Table 3a.** Performance of the assays under consideration for detection of pharyngeal and rectal *Neisseria gonorrheae* and *Chlamydia trachomatis* for male participants.

|  | **Positive percent agreement (95% CI)** | **Negative percent agreement (95% CI)** | **Positive predictive value* (95% CI)** | **Negative predictive value (95% CI)** | **Positive likelihood ratio (95% CI)** | **Negative likelihood ratio (95% CI)** |  |
| --- | --- | --- | --- | --- | --- | --- | --- |
| **Neisseria gonorrhoeae** | | | | | | | |
| **Pharynx** (N = 2053) |  | | | | | |  |
| Assay 1 | 95.0 (91.0, 97.3) | 98.5 (97.8, 98.9) | 87.2 (82.1, 91.0) | 99.5 (99.0, 99.7) | 62 (43, 90) | 0.05 (0.03, 0.09 |  |
| Assay 2 | 95.0 (91.0, 97.3) | 98.5 (97.8, 98.9) | 88.4 (83.4, 92.0) | 99.5 (99.0, 99.7) | 63 (43, 91) | 0.05 (0.03, 0.09) |  |
| Assay 3 | 84.5 (78.9, 88.8) | 99.5 (99.0, 99.7) | 94.6 (90.3, 97.0) | 98.3 (97.6, 98.8) | 155 (83, 288) | 0.16 (0.11, 0.21) |  |
| **Rectum** (N = 2050) |  |  |  |  |  |  |  |
| Assay 1 | 91.5 (86.8, 94.6) | 99.6 (99.1, 99.8) | 95.8 (92.0, 97.9) | 99.1 (98.5, 99.4) | 207 (103, 413) | 0.09 (0.05, 0.13) |  |
| Assay 2 | 96.4 (92.8, 98.3) | 99.1 (98.5, 99.4) | 92.6 (88.2, 95.5) | 99.7 (99.4, 99.9) | 105 (65, 168) | 0.04 (0.02, 0.07) |  |
| Assay 3 | 89.5 (84.5, 93.0) | 99.4 (99.0, 99.7) | 94.7 (90.5, 97.1) | 98.8 (98.2, 99.2) | 162 (87, 300) | 0.11 (0.07, 0.16) |  |
| **Chlamydia trachomatis** | | | | | | | |
| **Pharynx** (N = 2053) |  | | | | | |  |
| Assay 1 | 95.6 (85.2, 98.8) | 99.6 (99.3, 99.8) | 86.0 (73.8, 93.0) | 99.9 (99.6, 100.0) | 272 (130, 572) | 0.04 (0.01, 0.17) |  |
| Assay 2 | 89.1 (77.0, 95.3) | 99.7 (99.3, 99.8) | 85.4 (72.8, 92.8) | 99.8 (99.4, 99.9) | 255 (121, 538) | 0.11 (0.05, 0.25) |  |
| Assay 3 | 86.7 (73.8, 93.7) | 99.7 (99.3, 99.9) | 88.6 (76.0, 95.0) | 99.7 (99.3, 99.9) | 288 (129, 646) | 0.13 (0.06, 0.28) |  |
| **Rectum** (N = 2050) |  |  |  |  |  |  |  |
| Assay 1 | 85.1 (79.5, 89.4) | 99.3 (98.8, 99.6) | 92.7 (88.0, 95.7) | 98.4 (97.7, 98.9) | 119 (69, 204) | 0.15 (0.11, 0.21) |  |
| Assay 2 | 87.9 (82.5, 91.8) | 98.8 (98.1, 99.2) | 90.3 (85.1, 93.8) | 99.0 (98.5, 99.4) | 71 (47, 107) | 0.12 (0.08, 0.18) |  |
| Assay 3 | 82.1 (76.2, 86.9) | 98.9 (98.3, 99.3) | 89.0 (83.5, 92.7) | 98.1 (97.3, 98.6) | 74 (48, 116) | 0.18 (0.13, 0.24) |  |

*Subgroup-specific positive and negative predictive values were calculated based on the positivity observed for the subgroup.

# **Supplementary Table 3b.** Performance of the assays under consideration for detection of pharyngeal and rectal *Neisseria gonorrheae* and *Chlamydia trachomatis* for female participants.

|  | **Positive percent agreement (95% CI)** | **Negative percent agreement (95% CI)** | **Positive predictive value* (95% CI)** | **Negative predictive value (95% CI)** | **Positive likelihood ratio (95% CI)** | **Negative likelihood ratio (95% CI)** |  |
| --- | --- | --- | --- | --- | --- | --- | --- |
| **Neisseria gonorrhoeae** | | | | | | | |
| **Pharynx** (N = 537) |  | | | | | |  |
| Assay 1 | 83.3 (43.6, 97.0) | 99.8 (98.9, 100.0) | 83.3 (43.6, 97.0) | 99.8 (98.9, 100.0) | 442 (60, 3233) | 0.17 (0.03, 1.00) |  |
| Assay 2 | 100.0 (56.6, 100.0) | 99.8 (98.9, 100.0) | 83.3 (43.6, 97.0) | 100.0 (99.3, 100.0) | Not evaluable | Not evaluable |  |
| Assay 3 | 100.0 (56.6, 100.0) | 99.8 (98.9, 100.0) | 83.3 (43.6, 97.0) | 100.0 (99.3, 100.0) | Not evaluable | Not evaluable |  |
| **Rectum** (N = 535) |  |  |  |  |  |  |  |
| Assay 1 | 80.0 (37.6, 96.4) | 99.8 (98.9, 100.0) | 80.0 (37.6, 96.4) | 99.8 (98.9, 100.0) | 421 (57, 3130) | 0.20 (0.03, 1.16) |  |
| Assay 2 | 100.0 (51.0, 100.0) | 99.8 (98.9, 100.0) | 100.0 (51.0, 100.0) | 100.0 (99.3, 100.0) | Not evaluable | Not evaluable |  |
| Assay 3 | 40.0 (11.8, 76.9) | 100.0 (99.3, 100.0) | 100.0 (34.2, 100.0) | 99.4 (98.3, 99.8) | 55e12 (55e12, 55e12) | Not evaluable |  |
| **Chlamydia trachomatis** | | | | | | | |
| **Pharynx** (N = 537) |  | | | | | |  |
| Assay 1 | 100.0 (51.0, 100.0) | 99.8 (98.9, 100.0) | 80.0 (37.6, 96.4 | 100.0 (99.3, 100.0) | Not evaluable | Not evaluable |  |
| Assay 2 | 80.0 (37.6, 96.4) | 99.8 (98.9, 100.0) | 80.0 (37.6, 96.4 | 99.8 (98.9, 100.0) | 426 (57, 3166) | 0.20 (0.03, 1.16) |  |
| Assay 3 | 60.0 (23.1, 88.2) | 100.0 (99.3, 100.0) | 100.0 (43.9, 100.0) | 99.6 (98.6, 99.9) | Not evaluable | Not evaluable |  |
| **Rectum** (N = 535) |  |  |  |  |  |  |  |
| Assay 1 | 91.2 (77.0, 97.0) | 99.4 (98.2, 99.8) | 91.2 (77.0, 97.0) | 99.4 (98.2, 99.8) | 151 (49, 469) | 0.09 (0.03, 0.26) |  |
| Assay 2 | 93.8 (79.9, 98.3) | 98.6 (97.1, 99.3) | 81.1 (65.8, 90.5) | 99.6 (98.5, 99.9) | 67 (32, 140) | 0.06 (0.02, 0.24) |  |
| Assay 3 | 87.9 (72.7, 95.2) | 99.8 (98.8, 100.0) | 96.7 (83.3, 99.4) | 99.2 (97.9, 99.7) | 429 (60, 3051) | 0.12 (0.05, 0.30) |  |

*Subgroup-specific positive and negative predictive values were calculated based on the positivity observed for the subgroup.
