# Supplementary material for: Simultaneous Evaluation of Diagnostic Assays for Pharyngeal and Rectal Neisseria gonorrhoeae and Chlamydia trachomatis Using a Master Protocol
Source: Clin Infect Dis. 2019 Nov 17;71(9):2314–22. doi: 10.1093/cid/ciz1105 (PMC7713680; doi:10.1093/cid/ciz1105)
Supplement: ciz1105_suppl_Supplementary_data [file ciz1105_suppl_supplementary_data.pdf]

This supplement contains the following items:

1. Original protocol (Version 1), final protocol (Version 4), and summaries of changes from Version 1 to Version 2, Version 2 to Version 3, and Version 3 to Version 4.
2. Original statistical analysis plan (SAP V1.0) and final statistical analysis plan (SAP V2.0) Page 2 of SAP V2.0 contains a summary of changes on page 2.

Antibacterial Resistance Leadership Group (ARLG)

Performance of Nucleic Acid Amplification Tests for the Detection of *Neisseria gonorrhoeae* and *Chlamydia trachomatis* in Extragenital Sites (pNAAT)

Protocol Number:

ARLG_pNAAT-Yr3

Funding Sponsor:

National Institute of Allergy and Infectious Diseases (NIAID)

Funding Mechanism: 5UM1AI104681

**Protocol Date:**	April 25, 2016
**Protocol Version:**	1.0
**Principal Investigator**	Dr. Jeffrey D. Klausner, MD, MPH

Signature Page

The signature below documents the review and approval of this protocol and provides the necessary assurances that this study will be conducted according to the protocol, including all statements regarding confidentiality, and according to national, regional, and local legal and regulatory requirements.

Site Principal Investigator Name (Print)

Signature Date

ARLG GC Protocol Team Roster

All questions concerning this protocol should be sent via email to arlg.gc@mc.duke.edu. The appropriate team member will respond to questions via email, generally within 24 hours (Monday-Friday).

Protocol Chair

Jeffrey D. Klausner, MD, MPH
Professor of Medicine and Public Health
University of California, Los Angeles
10833 Le Conte Avenue
Los Angeles, CA 90095
Phone: 310-267-0409
Email: JDKlausner@mednet.ucla.edu

Project Leader

Elizabeth Petzold, PhD
Duke Clinical Research Institute
2400 Pratt Street
Durham NC 27705
Phone: 919-668-8876
Cell: 919-717-2061
Email: Elizabeth.petzold@duke.edu

Statisticians

Scott Evans, PhD
Harvard School of Public Health FXB 513
651 Huntington Avenue
Boston, MA 02115-6017
Phone: 617-432-2998
Email: evans@sdac.harvard.edu

Lauren Komarow, MSc
Harvard School of Public Health FXB 549A
651 Huntington Avenue
Boston, MA 02115-6017
Phone: 617-432-3233
Email: lkomarow@sdac.harvard.edu

Thuy Tran, MSc
Harvard School of Public Health FXB 608
651 Huntington Avenue
Boston, MA 02115-6017
Phone: 617-432-7523
Email: ttran@sdac.harvard.edu

Protocol Clinicians

Sarah Doernberg, MD, MAS
Division of Infectious Diseases
University of California, San Francisco
513 Parnassus Ave, Box 0654
San Francisco, CA 94143
Phone: 415-502-5548
Email: sarah.doernberg@ucsf.edu

Ephraim L. Tsalik, MD, MHS, PhD
Department of Medicine
Duke University Medical Center
Hanes House, Room 157,
Trent Drive,
Durham NC 27710
Phone: 919-684-3114
Email: e.t@dm.duke.edu

Table of Contents

LIST OF ABBREVIATIONS AND DEFINITIONS 6

1.0 BACKGROUND AND SCIENTIFIC RATIONALE 9

1.1 Background Information 9
1.2 Scientific Rationale 9

2.0 OBJECTIVES 11

2.1 Primary Objective 11
2.2 Secondary Objectives 11
2.3 Outcome Measures 12

3.0 STUDY DESIGN 14

3.1 Study Design 14
3.2 Inclusion Criteria 15
3.3 Exclusion Criteria 15

4.0 STUDY PROCEDURES 16

4.1 Recruitment Plan 16
4.2 Screening 16
4.3 Enrollment 16
4.6 Follow-up Visit 17

5.0 DEVICE MONITORING 17

5.1 Specimen Collection Device Monitoring 17
5.2 Laboratory Device Monitoring 17

6.0 STATISTICS 18

6.1 Study Design 18
6.2 Endpoints 18
6.3 Sample Size 18
6.4 Randomization of Swabs 19
6.5 Monitoring 19
6.6 Analyses 20

7.0 QUALITY CONTROL 23

7.1 Quality Controls and Study Monitoring 23
7.2 Source Documents and Access to Source Data........ 23

8.0 ETHICS / PROTECTION OF HUMAN SUBJECTS 23

8.1 Ethical Standards........ 23
8.2 Institutional Review Board and Informed Consent 23
8.3 Subject Confidentiality 23
8.4 Specimen Handling........ 24

9.0 DATA HANDLING AND RECORD KEEPING 24

9.1 Data Collection........ 24
9.2 Inspection of Records 24
9.3 Retention of Records........ 25
9.4 Confidentiality 25

10.0 PUBLICATION POLICY 25

APPENDIX A - SCHEDULE OF EVENTS........ 30

List of Abbreviations and Definitions

ARLG	Antibacterial Resistance Leadership Group
ASIS	Anatomic Site Infection Status
CDC	Center for Disease Control and Prevention
CFR	Code of Federal Regulations
CT	*Chlamydia trachomatis*
DCRI	Duke Clinical Research Institute
DNA	Deoxyribonucleic acid
Equivocal	Final equivocal result from nucleic acid amplification test, as determined by manufacturer’s guidance. This means the test was run and the result was neither positive nor negative
FDA	Food and Drug Administration
HIV	Human immunodeficiency virus
ICMJE	International Committee of Medical Journal Editors
ID	Identification
IFU	Instructions for Use
Indeterminate	Anatomic site infected standard cannot be determined as positive or negative
IRB	Institutional Review Board
ISRC	Independent Study Review Committee
LGBT	Lesbian, gay, bisexual and transgender
NAAT	Nucleic acid amplification test
Negative (-) NAAT reading	Result from the nucleic acid amplification test is negative
NR	This means no test was run and there is no result
NPA	Negative percent agreement
NPV	Negative predictive value
NG	*Neisseria gonorrhoeae*
NIH	National Institutes of Health
OHRP	Office of Human Research Protections
Positive (+) NAAT reading	Result from the nucleic acid amplification test is positive
PPA	Positive percent agreement
PPV	Positive predictive value
PI	Principal Investigator
RNA	Ribonucleic acid

rRNA	Ribosomal ribonucleic acid
SDMC	Statistical and Data Monitoring Center
STD	Sexually transmitted diseases
WHO	World Health Organization

Protocol Synopsis

Protocol Title:	Performance of Nucleic Acid Amplification Tests for the Detection of *Neisseria gonorrhoeae* and *Chlamydia trachomatis* in Extragenital Sites
Study Design:	A cross-sectional, single visit study to evaluate the diagnostic accuracy of nucleic acid amplification tests for detection of *Neisseria gonorrhoeae* and *Chlamydia trachomatis* from pharyngeal and rectal sites
Primary Study Objectives:	For each nucleic acid amplification assay, estimate the positive percent agreement (PPA) and negative percent agreement (NPA) for detecting:<br>• *Neisseria gonorrhoeae* in rectal swabs<br>• *Neisseria gonorrhoeae* in pharyngeal swabs<br>• *Chlamydia trachomatis* in rectal swabs<br>• *Chlamydia trachomatis* in pharyngeal swabs
Study Population	Patients presenting to an outpatient clinic for sexually transmitted disease (STD) testing for *Neisseria gonorrhoeae* and *Chlamydia trachomatis* infections
Number of subjects	Up to 2,500
Study duration	Approximately 12 months
Number of sites	Up to 10 clinical sites (as needed)
Clinical Samples	Pharyngeal and rectal swabs

1.0 Background and Scientific Rationale

1.1 Background Information

Nucleic acid amplification tests (NAATs) have become the gold standard for diagnosing *Neisseria gonorrhoeae* (NG) and *Chlamydia trachomatis* (CT) infections in the urogenital tract but are not currently approved by the Food and Drug Administration (FDA) for use in extragenital sites, including the pharynx and the rectum.

The goal of this study is to determine the diagnostic accuracy of the following 3 NAAT platforms for the detection of NG and CT from the pharynx and the rectum:

- Xpert® CT/NG Assay [Cepheid][1]
- Combo 2 ® Assay [Hologic][2]
- ProbeTec™ Q^x CT and GC Amplified DNA Assays [BD][3,4]

The assays being evaluated are intended for the direct qualitative detection of *Chlamydia trachomatis* and/or *Neisseria gonorrhoeae* in rectal or pharyngeal clinician-collected swabs from symptomatic or asymptomatic male or female patients.

1.2 Scientific Rationale

Infections due to NG and CT are a major public health threat. The World Health Organization (WHO) estimated more than 100 million new worldwide cases for each of NG and CT in 2008.[5] In the US alone, there were 333,000 NG and 1.4 million CT genitourinary infections in the year 2013.[6] Over the past 15 years, researchers have demonstrated an important burden of NG and CT infection in extragenital sites - the pharynx and rectum. Most CT and NG infections are asymptomatic, but infection can lead to serious sequelae, including infertility, chronic pelvic pain, adverse obstetrical outcomes, increased risk of human immunodeficiency virus (HIV) acquisition, and disseminated infection.[7-10] In addition to threats to individual health, there has been growing concern over antibiotic resistance, and in 2013, the US Centers for Disease Control and Prevention (CDC) classified drug-resistant NG as one of the three urgent-level resistant bacteria. Improved detection of extragenital NG is thought to be a crucial component of adequate treatment and thus prevention of further resistance.[11]

Currently, the CDC recommends the use of NAATs for NG and CT screening and diagnosis in the genitourinary tract due to superior sensitivity compared to traditional culture methods.[12] The sensitivity and specificity of commercially available NAATs for the detection of genitourinary NG and CT infection is estimated to be between 90-100%.[13] CDC also recommends the use of NAATs for extragenital sites.[12] Due to widespread use and interest in these tests for extragenital NG and CT infections, multiple studies have examined the test characteristics of NAAT tests in comparison to culture and to additional NAAT platforms (see Table 1). For diagnosis of CT, reported sensitivities are 80-100% (pharyngeal) and 46-100% (rectal), with significant variation by platform tested.[14-20] Reported specificities are >99% for pharyngeal site and 89-100% for the rectum. For diagnosis of NG, reported sensitivities are 72-100% (pharyngeal) and 75-100% (rectal), again with variation by platform.[14,15,17-19,21,22] Reported specificities are 72-100% (pharyngeal) and 95-100% (rectal).

Table 1. Prior Studies of extragenital testing for chlamydia and gonorrhea.

Screening test	Sensitivity/specificity* by specimen type (%)	
	**Pharyngeal**	**Rectal**
Neisseria gonorrhoeae		
**Hologic Gen-Probe Aptima Combo 2**		
Bachmann LH *et al,* 2010[17]	100 / 96.2	100 / 95.5
Cosentino LA *et al,* 2012[18]		76 / 100
Moncada J *et al,* 2009[20]		78.3 / 99.8
Ota KV et al, 2009[14]	95 / 99.6	100 / 100
Schachter J *et al*, 2008[15]	84.3 / 99.4	93.2 / 99.7
**BD ProbeTec CT/GC Q^X Assays**		
Pope CF *et al*, 2010[23]	100 / 98.4	100 / 100
Valencia F *et al*, 2013[24]	100 / 100	100 / 100
Gratzer B et al, 2012[25]	94.3 / 96.7	100 / 99.2
Van Der Pol B *et al*,2013[26]	97.4 / 100	
**Cepheid Xpert® CT/NG Assay**		
Goldenberg SD *et al*., 2012[27]		91.1 / 100
Cosentino LA *et al*, 2015[28]		100 / 100
Chlamydia trachomatis		
**Hologic Gen-Probe Aptima Combo 2**		
Bachmann LH *et al,* 2010[17]		100 / 88.8
Cosentino LA *et al,* 2012[18]		100 / 99.8
Moncada J *et al,* 2009[20]		71.2 / 99
Ota KV *et al*, 2009[14]	100 / 99.2	100 / 98.7
Schachter J *et al,* 2008[15]	100 / 99.6	93.5 / 97.7
**BD ProbeTec CT/GC Q^X Assays**		
Valencia F *et al,* 2013[24]	93 / 100	80 / 98
Van Der Pol B *et al*. 2013[26]	100 / 100	
**Cepheid Xpert® CT/NG Assay**		
Goldenberg SD *et al*., 2012[27]		86 / 99.2
Cosentino LA *et al*, 2015[28]		96.6 / 99.7

*Sensitivity and specificity as reported by authors, each using unique definitions for the reference standard. As discussed below, we will be using positive percent agreement (PPA) and negative percent agreement (NPA) for these calculations.

Despite the CDC recommendations, at this time, there are no FDA-cleared commercial NAAT tests for the detection of pharyngeal and rectal NG or CT infection. The FDA-clearance of such assays would increase the availability and uptake and subsequently lead to improved screening and enhanced control of NG and CT extragenital infections. The current proposed study will help provide the data required for FDA consideration to clear such assays for marketing.

Because of the multi-site nature of the ARLG, its strong laboratory network and inclusion of investigators with expertise in NG and CT detection and treatment, the ARLG is well-positioned to conduct such a study. Current commercial assays are “dual” assays and include targets for both NG and CT, so the goal of this study will be to evaluate the diagnostic accuracy for detection of both organisms at extragenital sites.

The molecular targets of the three NAAT platforms that will be tested differ, which will allow for comparative evaluation.[29]

- Xpert® CT/NG Assay (Cepheid) is a combination test that uses a real-time PCR technique to detect two noncontiguous chromosomal DNA regions from NG (NG2 and NG4) - both of which must be positive to yield a positive result - and one chromosomal DNA target from CT (CT1) [1]
- Combo 2 ® Assay (Hologic) is a combination test that utilizes target capture, transcription mediated amplification, and dual kinetic assay to detect regions of the ribosomal RNA (rRNA) from the 16S rRNA of GC and the 23S rRNA from CT using labeled DNA probes [2]
- ProbeTec™ Q^x *Chlamydia trachomatis* and *Neisseria gonorrhoeae* Amplified DNA Assays (BD) employ strand displacement amplification with a fluorescently-labeled probe to detect NG chromosomal DNA coding the pilin gene-inverting protein homologue and a region within the CT cryptic plasmid DNA [3,4]

Additional testing using the Aptima® *Chlamydia trachomatis* assay (Hologic)[30] and/or the Aptima® *Neisseria gonorrhoeae* assay (Hologic)[31] will be performed in cases of discordant results (see Section 3.5). This test utilizes target capture, transcription mediated amplification, and hybridization protection assays to identify the presence of RNA from the organism of interest in the clinical sample. The targets from the 16S rRNA for both GC and CT are different than those used in the Combo 2® Assay.[2,30,31] Though the diagnostic accuracy of these assays for extragenital infections has not previously been evaluated, studies evaluating the performance of these assays for confirmation of other NAATs suggest excellent concordance.[32-35]

2.0 Objectives

2.1 Primary Objective

For each NAAT, estimate the PPAs and NPAs for detecting:

- *Neisseria gonorrhoeae* in rectal swabs
- *Neisseria gonorrhoeae* in pharyngeal swabs
- *Chlamydia trachomatis* in rectal swabs
- *Chlamydia trachomatis* in pharyngeal swabs

2.2 Secondary Objectives

2.2.1 Global analyses:

For each NAAT, positive predictive values (PPVs), negative predictive values (NPVs), positive likelihood ratios, negative likelihood ratios, and sensitivity analyses will be calculated for detecting:

- *Neisseria gonorrhoeae* in rectal swabs
- *Neisseria gonorrhoeae* in pharyngeal swabs
- *Chlamydia trachomatis* in rectal swabs
- *Chlamydia trachomatis* in pharyngeal swabs

2.2.2 Subgroup analyses:

For each NAAT, to estimate the PPAs, NPAs, PPVs, and NPVs for detection of NG and CT from rectal and pharyngeal swab specimens by sex and by anatomic site-specific symptom status.

2.3 Outcome Measures

The anatomic site infected status (ASIS) will be determined for each anatomic site (pharyngeal and rectal) and each organism (NG and CT), as shown below in Table 2.

Possible ASIS outcomes are:

- Infected
- Not infected
- Indeterminate
- Invalid, exclude from analysis

The anatomic site is considered to be infected if both of the reference test results are positive. The anatomic site is considered to be not infected when both reference test results are negative. If there is discordance between the reference tests, an additional NAAT will be done as a tiebreaker. In this case, agreement of 2/3 of the reference NAATs will determine the ASIS. If two tests are equivocal or one equivocal and one not run, the third test result will stand as the ASIS if positive or negative. If two tests are not run, the ASIS will be considered invalid and will be excluded from the analysis. All possible combinations are shown in Table 2. The tiebreaker test will be run by the lab if any NAAT is not concordant with the others and interpreted only in the case of discordant results between the two planned reference tests for each assay. As the tiebreaker test is not a combination test, the tiebreaker will only be run for the organism with disagreement (e.g. if NG disagrees and CT agrees, the tiebreaker will only be run for NG).

To determine the ASIS, the test result for each respective site (pharyngeal or rectal) and each organism (NG or CT) for each NAAT platform will be used. Possible outcomes for each assay are:

- **Xpert® CT/NG Assay (Cepheid)**[1]: Not detected, detected, invalid (sample processing control or sample adequacy control failed), error (probe check control failed), or no result (insufficient data was collected, e.g. test aborted). Initial invalid, error, or no result tests will be repeated. If the repeat test returns invalid, error, or no result, the final result will be considered an invalid and will be categorized as <u>no result (NR)</u> for the ASIS determination below. If the repeat test returns not detected (negative) or detected (positive), this will be the result used for the ASIS determination.

- **Combo 2 ® Assay (Hologic)**[2]: Negative, positive, equivocal (result between positive and negative), or invalid (parameter outside the normal expected ranges). Initial equivocal and invalid test results will be repeated. If the repeat test result is equivocal or invalid, the final test result will be considered an <u>equivocal test result</u> for the ASIS determination below. If the repeat test returns negative or positive, this will be the result considered for the ASIS determination.

- **ProbeTec™ Q^{x} *Chlamydia trachomatis* and *Neisseria gonorrhoeae* Amplified DNA Assays (BD)**[3,4]: Negative, positive, extraction control failure, extraction transfer failure, liquid level failure, and error. Initial extraction control failure, extraction transfer failure, liquid level failure, and error results will be repeated. If the repeat test result is extraction control failure, extraction transfer failure, liquid level failure, or error, the final result will be considered an invalid result and will be categorized as <u>no result (NR)</u> for the ASIS determination below. If the repeat test returns negative or positive, this will be the result considered for the ASIS determination.

- **<u>Tiebreaker assays:</u> Aptima® *Chlamydia trachomatis* assay (Gen-Probe)[30] and the Aptima® *Neisseria gonorrhoeae* assay (Gen-Probe)[31]**: Negative, positive, equivocal (result between negative and positive ranges), or invalid (parameter outside the normal expected ranges). Initial equivocal and invalid test results will be repeated. If the repeated test remains equivocal or invalid, it will be considered an <u>equivocal test result</u> for the ASIS determination below. If the repeat test returns negative or positive, this will be the result considered for the ASIS determination.

Table 2. Determination of the Anatomic Site Infected Standard

Comparator NAAT 1 Result	Comparator NAAT 2 Result	Tiebreaker NAAT Result	Anatomic Site Infection status
+	+	Not indicated	Infected
+	-	+	Infected
+	E*	+	Infected
+	NR**	+	Infected
+	-	-	Not infected
+	-	E	Indeterminate
+	-	NR	Indeterminate
+	E	-	Indeterminate
+	E	E	Infected
+	E	NR	Infected
+	NR	-	Indeterminate
+	NR	E	Infected
+	NR	NR	Invalid,<br>remove from analysis
-	-	Not indicated	Not infected
-	+	-	Not infected
-	E	-	Not infected
-	NR	-	Not infected
-	+	+	Infected
-	+	E	Indeterminate
-	+	NR	Indeterminate
-	E	+	Indeterminate
-	E	E	Not infected
-	E	NR	Not infected
-	NR	+	Indeterminate
-	NR	E	Not infected
-	NR	NR	Invalid,<br>remove from analysis
E	+	+	Infected
E	-	-	Not infected
E	+	-	Indeterminate

E	+	E	Infected
E	+	NR	Infected
E	-	+	Indeterminate
E	-	E	Not infected
E	-	NR	Not infected
E	NR	+	Infected
E	NR	-	Not infected
E	NR	E	Indeterminate
E	NR	NR	Invalid, remove from analysis
NR	+	+	Infected
NR	-	-	Not infected
NR	NR	Not indicated	Invalid, remove from analysis
NR	+	-	Indeterminate
NR	+	E	Infected
NR	+	NR	Indeterminate
NR	-	+	Indeterminate
NR	-	E	Not infected
NR	-	NR	Invalid, remove from analysis
NR	E	+	Infected
NR	E	-	Not infected
NR	E	E	Indeterminate
NR	E	NR	Invalid, remove from analysis

*E **=** equivocal result
******NR = no result. This can occur either because the test result was invalid or because the test could not be run (e.g. too little sample, improperly shipped, no sample received).

3.0 Study Design

3.1 Study Design

This will be a cross-sectional, single visit study evaluating the performance of three commercial NAATs to detect NG and CT in the rectum and pharynx.[1-4] A fourth NAAT is being used as a tiebreaker.[30,31] We are not evaluating the performance of this fourth NAAT.

The study will include specimens collected from adult subjects seeking sexually transmitted disease (STD) testing at the participating clinics, which include clinics focused on sexually transmitted diseases, women's health, student health, family planning clinics, and clinics specializing in lesbian, gay, bisexual, and transgender (LGBT) health. Potential subjects will be identified, assessed for eligibility and

approached for informed consent. Both symptomatic and asymptomatic individuals will be included in the study population.

The study will enroll approximately 2,500 subjects who will have four pharyngeal and four rectal swabs collected as part of a one-time study visit. Each manufacturer will have specific collection kit and transport media. Additional data collection will include, but is not limited to, eligibility screening questions, gender, age, and patient report of relevant pharyngeal, rectal, or other relevant symptoms, and swab collection completeness. Subjects will be classified as symptomatic or asymptomatic for each anatomic site. Results of testing at other anatomic sites and results of clinical testing will not be available.

Each swab will be used for a specific NAAT, which will be run at one of the two reference testing laboratories. The ASIS will be defined by the results of the two other nucleic acid amplification tests, and a tiebreaker test (if necessary), as shown in Table 2. Each anatomic site will be considered in isolation.

When accrual is complete, the study will be closed and an analysis plan will be enacted to finalize the database for analysis. The study duration is expected be approximately 6-12 months.

3.2 Inclusion Criteria

a) Attending a participating clinic for evaluation of STDs
b) ≥18 years of age at date of screening
c) Able and willing to provide informed consent
d) Willing to comply with study procedures, including collection of 4 swabs each from the pharynx and rectum for NG and CT testing

NOTE: Male, female, and transgender individuals will be included, and both symptomatic and asymptomatic individuals will be included.

3.3 Exclusion Criteria

a) Receipt of any systemic antibacterial drug in the past 14 days
b) Receipt of myelosuppressive chemotherapy in the past 30 days

3.4 Nucleic Acid Amplification Tests (NAATs)

This study will evaluate three distinct NAAT platforms. The selected platforms[29] are:

- Xpert® CT/NG Assay (Cepheid) uses a real-time PCR technique to detect two noncontiguous chromosomal DNA regions from NG (NG2 and NG4) - both of which must be positive to yield a positive result - and one chromosomal DNA target from CT (CT1). [1]

- APTIMA Combo 2® Assay (Hologic) utilizes target capture, transcription mediated amplification, and dual kinetic assay to detect regions of the rRNA from the 16S rRNA of GC and the 23S rRNA from CT using labeled DNA probes.[2]

- ProbeTec™ Q^x *Chlamydia trachomatis* and *Neisseria gonorrhoeae* Amplified DNA Assays (BD) employ strand displacement amplification with a fluorescently-labeled probe to detect NG chromosomal DNA coding the pilin gene-inverting protein homologue and a region within the CT cryptic plasmid DNA.[3,4]

3.5 Tiebreaker Assay

In case of disagreement between reference NAAT results, a tiebreaker assay will be performed. This assay will use the Hologic Tigris platform with the Aptima® *Chlamydia trachomatis* assay and/or the Aptima® *Neisseria gonorrhoeae* assay (see Section 2.3). These tests utilize target capture, transcription mediated amplification, and hybridization protection assays to identify the presence of RNA from the organism of interest in the clinical sample. The targets from the 16S rRNA for both GC and CT are different than those used in the Combo 2® Assay.[30,31]

4.0 Study Procedures

4.1 Recruitment Plan

This study will enroll 2,500 participants who are seeking STD testing at one of the participating clinics. Potential subjects will be recruited without regard for risk behavior, symptom status, or gender. Participating clinics may include those designed for STD screening and management, family planning, student health, women's health, HIV management, and clinics focusing on the LGBT population.

4.2 Screening

Participants will be recruited from clinical sites performing routine STD testing. It is assumed that individuals presenting for routine STD testing at the participating clinics are at risk for such infections. Each person will undergo screening for eligibility and, if eligible, will be approached for oral informed consent.

4.3 Enrollment

Once enrolled, eight swabs will be taken: four swabs will be taken from the pharynx and four swabs will be taken from the rectum in addition to any swabs taken as part of routine clinical care. The swabs collected for routine clinical care will be taken first. The order of the research swabs will be randomized per subject to account for the possibility that the yield of the swab is affected by previous swabs at the same anatomic site. The swabs will be stored and transported per manufacturer guidelines. Results of those NAATs will <u>not</u> be reported back to the treating clinician for clinical purposes.

Data collection will include, but is not limited to, assessment of signs and symptoms of rectal and/or pharyngeal infection, as well as socio-demographic information such as age (not date of birth), race, ethnicity and gender, and swab collection completeness.

4.5 Incomplete Swab Collection

While required as an inclusion criteria for a potential subject to agree to both pharyngeal and rectal swabs, if only one anatomic site is swabbed after enrollment for whatever reason, and all four swabs are completed for that anatomic site, those swabs for that anatomic site will be included in the analysis. If fewer than four swabs at a site are completed, that anatomic site (and swab results) will be excluded from the analysis.

4.4 Subject Withdrawal and Replacement

Subjects may voluntarily withdraw their consent for study participation at any time and for any reason, without penalty. The primary reason for withdrawal from the study will be recorded on the Study Status case report form. Subjects who withdraw will not be replaced.

4.5 Study Discontinuation

This study may be terminated at any time by the Principal Investigator (PI) in consultation with the Antibacterial Resistance Leadership Group (ARLG) and/or the National Institutes of Health (NIH).

4.6 Follow-up Visit

There will not be a follow-up visit as part of this study. Subjects will continue with routine clinical care as directed by their medical providers.

4.7 Central Laboratory Procedures

There will be two reference testing laboratories, which will divide the processing and running of the NAATs for the study. Each clinical study site will be assigned to send swabs to one of the two laboratories. Swabs will be processed and run at the reference testing laboratories according to each manufacturer's Instructions for Use (IFU). Initial equivocal, invalid, or otherwise undetermined results will be repeated per manufacturer's guidelines before determining whether the result will be classified as positive, negative, equivocal, or no result. One of the reference testing laboratories will be responsible for running all of the tiebreaker assays, regardless of the location of original study site.

The reference testing laboratories will receive training for each of the study platforms from a representative from each of the manufacturers, in order to ensure adherence to the manufacturer's IFU procedures.

5.0 Device Monitoring

5.1 Specimen Collection Device Monitoring

Any complications with the specimen collection process, including device failure, significant patient discomfort, or inability to collect specimen, will be reported on the specimen collection case report form.

5.2 Laboratory Device Monitoring

The reference testing laboratories will maintain a log of all unanticipated device-related complications leading to no test, such as absence of transport media, quantity not sufficient, specimen transport collection system damage or incorrect transport system.

6.0 Statistics

6.1 Study Design

This is a cross-sectional, single visit study to evaluate the diagnostic accuracy of NAATs for the detection of NG and CT from pharyngeal and rectal swab specimens. Study participants will have swabs collected from both the rectum and pharynx, following any specimens taken for clinically indicated testing. Each of the swabs from each anatomic location will be tested for both NG and CT using three different NAATs, with a 4th test collected and tested as a tiebreaker, if needed. As the tiebreaker is not a combination test, it will only be run for the organism with disagreement. Diagnostic accuracy will be calculated with respect to an anatomic site infected status reference standard described in Section 3.5. As the reference standard is imperfect, we will report positive percent agreement and negative percent agreement in place of sensitivity and specificity.

One potential limitation of this study is that all three of the tests in the reference standard are NAATs. However, all three tests use different nucleic acid targets (rRNA vs. DNA with different molecular primers targeting different aspects of the CT and NG genome [see Section 3.4]), different methods of target amplification (transcription-mediated amplification, polymerase chain reaction and strand displacement amplification) and different methods of target detection (hybrid capture, multi-color laser detection and fluorescent polarization). So while, theoretically, there may be correlation in the errors produced by the three tests, the varied molecular targets and the different methods of capture, amplification and detection should mitigate this concern.

6.2 Endpoints

- Infection status for *Neisseria gonorrhoeae* in the rectum as determined by each NAAT
- Infection status for *Neisseria gonorrhoeae* in the pharynx as determined by each NAAT
- Infection status for *Chlamydia trachomatis* in the rectum as determined by each NAAT
- Infection status for *Chlamydia trachomatis* in the pharynx as determined by each NAAT
- Anatomic Site Infection status as determined by the reference standard (as described in Section 2.3)

6.3 Sample Size

For each assay, PPA and NPA will be estimated for each of three diagnostic tests defined by pathogen - anatomical site combinations:

- *Neisseria gonorrhoeae* in the rectum
- *Neisseria gonorrhoeae* in the pharynx
- *Chlamydia trachomatis* in the rectum
- *Chlamydia trachomatis* in the pharynx

Each pathogen/anatomic site combination will be evaluated by constructing 95% Score confidence interval estimates for PPA and NPA and evaluating whether 90% can be ruled out by the lower bound of the CI with reasonable confidence.

NPA is expected to be greater than PPA and there will be considerably more not-infected than infected anatomic site, implying that there will be greater power for evaluating NPA. Thus it will suffice to size the study to evaluate PPA.

Assuming that the true PPA is 96%,[14] then 157, 174, and 196 evaluable participants that are infected are needed to have 80%, 85%, and 90% power respectively to demonstrate a PPA of greater than 90%. Since all of the participants will contribute to the evaluation of all three pathogen-anatomical site diagnostic tests, it will suffice to size the study for the diagnostic test with the lowest expected prevalence (except CT in the pharynx). The study will be more than sufficiently sized for more prevalent disease.

It is expected that the disease prevalence of NG in the rectum, NG in the pharynx, and CT in the rectum will each be greater than 7.5% in the population under evaluation.[14,15,17-21] Table 3 summarizes the required number of participants to have 80% and 90% probabilities of obtaining the 157, 174, and 196 evaluable participants that are disease-positive to have 80%, 85%, and 90% power when the true prevalence of disease is 7.5%, 10%, and 12.5%.

Table 3: Sample size required number to have 80% and 90% probabilities of obtaining the 157, 174, and 196 evaluable participants that are disease-positive when the true prevalence of disease is 7.5%, 10%, and 12.5% in order to have 80%, 85%, and 90% power (two-sided alpha=0.05) to conclude that sensitivity is greater than 90% when the true sensitivity is 96%.

Prevalence of Disease Positive	Power	Required Disease Positive to Obtain Power	Required Sample Size to have 80% probability of obtaining required Disease positive participants	Required Sample Size to have 90% probability of obtaining required Disease positive participants
**7.5%**	**80%**	157	2250	2325
	**85%**	174	2475	2575
	**90%**	196	2775	2875
**10%**	**80%**	157	1700	1750
	**85%**	174	1860	1925
	**90%**	196	2100	2150
**12.5%**	**80%**	157	1350	1400
	**85%**	174	1485	1530
	**90%**	196	1665	1715

6.4 Randomization of Swabs

Randomization will be employed to ensure that the swab order distribution is the same for 4 tests, the three NAATs under investigation in the study and the tiebreaker test. SAS software (Cary, NC) will be used to generate permuted blocks with a block size of 24, to ensure that each NAAT and the tiebreaker test would have their swab distributions consisting of 1/4 being first swabs, 1/4 being second swabs, 1/4 being third swabs and 1/4 being fourth swabs.

6.5 Monitoring

The core protocol team will receive monthly reports monitoring study accrual, study conduct and completeness of data and swab collection.

The study will be reviewed by an independent statistician, who will not have an association with the protocol or device companies. The independent statistician will review the progress of the study.

Additionally, the study statisticians will prepare reports for the independent statistician that will include baseline summaries (demographics and symptom status) and will address issues concerning the viability and appropriate execution of the protocol such as accrual, endpoint evaluability, and frequency of tests with equivocal results, invalid results, or no results. Issues with specimen collection will be summarized. The independent statistician will also review and evaluate the rates of disease. Toxicity is not expected given the nature of the study.

The study cannot be stopped at the interim for reaching regulatory goals at this time to preserve error rates / coverage probability and ensure enough data for subgroup analyses. Since the trial cannot be stopped for attainment of the regulatory goal, no adjustment to confidence levels are necessary.

Monitoring design assumptions for appropriate study sizing

Infection rates will be evaluated to determine whether sample size adjustments are warranted. The sample size will not be adjusted based on the observed PPAs and NPAs, which will not be reviewed while the study is ongoing, but may be adjusted based only on the infection rates to ensure enough infected participants to estimate PPAs with desired precision. If infection is more prevalent than expected, a smaller sample size may be accepted. If however infection is rarer than anticipated, then increases to sample size will be considered. The infection rate will be evaluated approximately every 500 participants or every 3 months after the enrollment of the first participant, whichever comes first.

6.6 Analyses

6.6.1 Primary Analyses

The result for each test will be compared with the ASIS for that anatomic site and organism. For each diagnostic test, PPA and NPA will be estimated using 95% Score confidence intervals. Confidence intervals will be estimated using the Score method.[36]

In the primary analysis, we will follow the FDA guidance document for incorporating indeterminate ASIS or test results for the test under consideration that are equivocal.[36] Indeterminate ASIS results will be counted against the results for the test under consideration, as described in Tables 4 and 5. Tables 4 and 5 will be presented in the analysis report, showing all combinations of Infected/Indeterminate/Not Infected with all outcomes from the test under consideration (Positive/Equivocal/Negative/No result or Invalid)

If the test under consideration has “no result” because the test was not run and no attempt was made to test the sample, it will be excluded from the primary analysis.

Table 4. Use of the ASIS in calculation of the positive and negative percent agreement for the Aptima Combo 2® Assay (Hologic).

	**ASIS**		
	Infected	Indeterminate	Not infected

**Result of Test under Consideration**	Positive	A	D	G
	Equivocal	B	E	H
	Negative	C	F	I
	No result	Exclude from analysis		
	PPA	A / (A+B+C+F)		
	NPA			I / (G + H + I + D)

Sensitivity analyses will be conducted considering multiple scenarios. These include:

- Classify indeterminate tests on the basis of symptom status. Include indeterminate tests as infected if the participant is symptomatic in that compartment. Include indeterminate tests as not infected in the participant is asymptomatic in that compartment.
- Include all indeterminate tests as infected.
  - PPA=(A+D) / (A+B+C+D+E+F)
  - NPA=I / (G+H+I))
- Include all indeterminate tests as not infected.
  - PPA=A / (A+B+C)
  - NPA=(F+I) / (D+E+F+G+H+I)
- Considering indeterminate and equivocal test results as “missing”, with the assumption of missing at random, and model the missing results.
- Additional sensitivity analyses will be performed. Details will be in the statistical analysis plan.

Table 5. Use of the ASIS in calculation of the positive and negative percent agreement for the Xpert® CT/NG Assay (Cepheid) and ProbeTec™ Q^x *Chlamydia trachomatis* and *Neisseria gonorrhoeae* Amplified DNA Assays (BD)

		**ASIS**		
		Infected	Indeterminate	Not infected
**Result of Test under Consideration**	Positive	A	C	E
	Negative	B	D	F
	No result/ Invalid	Exclude from analysis		
	PPA	A / (A+B+D)		
	NPA			F / (C+E+F)

If the test under consideration has “no result” because the test was not run and no attempt was made to test the sample or because of invalid ASIS results it will be excluded from the primary analysis.

Sensitivity analyses will be conducted. These include:

- Classify indeterminate tests on the basis of symptom status. Include indeterminate tests as Infected if the participant is symptomatic in that compartment. Include indeterminate tests as not infected in the participant is asymptomatic in that compartment.
- Include all indeterminate tests as infected
  - PPA = (A+C) / (A+B+C+D)
  - NPA = I / (E+F)
- Include all indeterminate tests as not infected
  - PPA = A / (A+B
  - NPA = (D+F) / (C+D+E+F)

- Additional sensitivity analyses that consider indeterminate results as “missing” and utilize imputation strategies, and graphical techniques
- Additional sensitivity analyses will be performed. Details will be in the statistical analysis plan.

There are pros and cons to the manner in which indeterminates are handled and the resulting calculations of PPA and NPA. The primary definition is the most conservative but is biased downwards. If PPA > 90% under this scenario, then the conclusion of PPA > 90% is clear. The second and third sensitivity analyses count indeterminates (treat all as infected or not infected, respectively) but may not be conservative for calculations of PPA and NPA.

6.6.2 Secondary Analyses

Global Analyses

For each diagnostic test:

- Positive and negative predictive values (PPV and NPV) will be estimated. PPV and NPV will each be plotted as a function of prevalence (point estimates and 95% pointwise confidence bands)
- Positive and negative likelihood ratios will be estimated using 95% Score confidence intervals
- A sensitivity analysis will include calculating equivocal predictive values and likelihood ratios
- An additional sensitivity analysis will calculate PPV, NPV, and positive and negative likelihood ratios for the sensitivity analyses where indeterminates are counted as Infected and Not Infected

Subgroup Analyses

For each of the three diagnostic tests, subgroup analyses will be conducted for males, females, symptomatic participants, and asymptomatic participants by pathogen and anatomic site. The 95% Score confidence interval estimates of PPA and NPA will be displayed using forest plots (one plot per test). The 95% Score confidence interval estimates of positive and negative likelihood ratios will be displayed using forest plots (one plot per test). PPVs and NPVs will each be plotted as a function of prevalence (point estimates and pointwise confidence bands).

The sensitivity analyses where indeterminates are counted as Infected and Not Infected will be repeated for the four subgroup analyses.

Exploratory Analysis

Developing methods for diagnostic benefit:risk analyses (e.g., diagnostic yield) will be applied.

7.0 Quality Control

7.1 Quality Controls and Study Monitoring

The ARLG will provide direct access to the dataset for the purposes of monitoring and auditing by the DCRI, Harvard School of Public Health and inspection by local and regulatory authorities. The local site PI will ensure that study personnel are appropriately trained and applicable documentations are maintained.

The DCRI will implement a Quality Plan to ensure that protocol training, data quality and data security are being undertaken.

7.2 Source Documents and Access to Source Data

Source documents will not be removed from the sites. Study monitoring will occur remotely, unless there are quality or performance concerns at a site, in which case an on-site visit will occur.

8.0 Ethics / Protection of Human Subjects

8.1 Ethical Standards

The investigator will ensure that the study will be conducted in accordance with all applicable national, regional, and local regulations.

8.2 Institutional Review Board and Informed Consent

This protocol and any subsequent modifications must be reviewed and approved by the IRB responsible for oversight of the study, as listed on its FWA. To minimize risks associated with loss of privacy and loss of confidentiality, oral informed consent will be obtained from the subject. The informed consent process will describe the purpose of the study, the procedures to be followed, and the risks and benefits of participation. A copy of a patient information form may be given to the subject (per local policy).

8.3 Subject Confidentiality

Subjects will have code numbers and will not be identified by name. Subject confidentiality is strictly held in trust by the participating investigators, their staff, and the Sponsor(s) and their agents.

The study protocol, documentation, data, and all other information generated will be held in strict confidence. No information concerning the study or the data will be released to any unauthorized third party without prior written approval of the ARLG.

All information provided by the ARLG and all data and information generated by the participating clinical site as part of the trial will be kept confidential by the site PI and other study personnel. This information and data will not be used by the site PI or other study personnel for any purpose other than conducting the trial. These restrictions do not apply to: (1) information which becomes publicly available through no fault of the site principal investigator or other study personnel; (2) information which is necessary to disclose in confidence to an IRB solely for the evaluation of the trial (3) information which

is necessary to disclose in order to provide appropriate medical care to a study subject; or (4) study results which may be published as described in Section 10.

The study monitor or other authorized representatives of the sponsor may inspect all documents and records required to be maintained by the PI. The clinical study site will permit access to such records.

All laboratory specimens, case report form data, reports and other records will be identified only by a coded number to maintain subject confidentiality.

8.4 Specimen Handling

Specimens will be collected for this study and will be maintained by the ARLG at least until all companies have received their FDA approval for their assay.

Use of samples for future research will be part of the consent process. There will **not** be any opt-in/opt-out language. Samples may be shared with other investigators at other institutions. No human genetic tests will be performed on samples. Each sample will be encoded (labeled) only with a barcode and a unique tracking number to protect subject's confidentiality.

There are no benefits to subjects in the collection, storage and subsequent research use of specimens. Because the subject enrollment will be done anonymously, reports about future research done with subject's samples will NOT be reported into their health records.

9.0 Data Handling and Record Keeping

9.1 Data Collection

This study will use eClinical OS (eCOS), a Web-based e-CRF database used by the DCRI. The investigator's site staff who will be entering data will receive training on the system, after which each person will be issued a unique user identification ID and password.

For security reasons, and in compliance with regulatory guidelines, it is imperative that only the persons who own the user IDs and passwords access the system using their own unique access codes. Access codes are nontransferable. Site personnel who have not undergone training may not use the system and will not be issued a user ID and password until appropriate training is completed.

9.2 Inspection of Records

The DCRI will implement a Quality Plan to, at a minimum, ensure that activities proposed by the PI to ensure protocol training, data quality and data security are being undertaken.

Source Documents and Access to Source Data

Source documents will not be removed from the sites.

9.3 Retention of Records

Records will be maintained during the investigation and for a period of 2 years after the latter of the following dates: The date on which the investigation is terminated or completed, or the date that the records are no longer required for purposes of supporting a premarket approval application or a notice of completion of a product development protocol. Records may not be destroyed without the prior approval of the ARLG.

9.4 Confidentiality

The study protocol, documentation, data, and all other information generated by this study will be maintained in a secure manner and will be kept confidential as required by law.

The EDC database (eClinical OS data) used by the DCRI will be hosted by Merge which uses a Peak 10 datacenter located in Morrisville NC. Database access will be limited to study personnel who are issued a unique user identification and password. Data will be entered at each site by study personnel. No information concerning the study or the data will be released to any third party without prior written approval of the Sponsor. Study records may be reviewed in order to meet federal or state regulations. Reviewers may include the IRBs, the DCRI and the NIH.

10.0 Publication Policy

Following completion of the study, the investigator may publish the results of this research in a scientific journal under the oversight of the Publication Committee of the ARLG.

The ARLG Publication Committee comprises representatives of the network cores, thought-leaders, statistics and data management center (SDMC), and is responsible for generation and coordination of the publications that report scientific findings of the network. All public presentations (abstracts, manuscripts, slides and text of oral or other presentations, and text of any transmission through any electronic media) by participating investigators, participating institutions, SDMC, and ARLG that use ARLG data and are intended to represent the ARLG or are supported by the ARLG will be reviewed by the Publication Committee per the Publication Committee charter and must include the following statement: “Research reported in this publication was supported by the National Institute Of Allergy And Infectious Diseases of the National Institutes of Health under Award Number UM1AI104681. The content is solely the responsibility of the authors and does not necessarily represent the official views of the National Institutes of Health.”

The Publication Committee will ensure that the study results are presented by experts in the field that have working knowledge of the study design, implementation, data synthesis/analysis, and interpretation. The committee goals are to ensure that any confidential or proprietary information is protected, and that all appropriate statistical analyses have been included.

The ARLG Publication Committee will adhere to the trials registration policy adopted by the International Committee of Medical Journal Editors (ICMJE) member journals. This policy requires that all applicable clinical trials be registered in a public trials registry such as ClinicalTrials.gov, which is sponsored by the National Library of Medicine. Other biomedical journals are considering adopting similar policies.

In June 2007, the ICMJE adopted the WHO's definition of clinical trial: "any research study that prospectively assigns human participants or groups of humans to one or more health-related interventions to evaluate the effects on health outcomes."

- Health-related interventions include any intervention used to modify a biomedical or health-related outcome (for example, drugs, surgical procedures, devices, behavioral treatments, dietary interventions, and process-of-care changes).
- Health outcomes include any biomedical or health-related measures obtained in patients or participants, including pharmacokinetic measures and adverse events.

All investigators funded by the NIH must submit or have submitted for them to the National Library of Medicine’s PubMed Central an electronic version of their final, peer-reviewed manuscripts upon acceptance for publication, to be made publicly available no later than 12 months after the official date of publication. The NIH Public Access Policy ensures the public has access to the published results of NIH-funded research. It requires investigators to submit final peer-reviewed journal manuscripts that arise from NIH funds to the digital archive PubMed Central upon acceptance for publication. Further, the policy stipulates that these papers must be accessible to the public on PubMed Central no later than 12 months after publication.

Refer to: http://publicaccess.nih.gov/

11.0 References

1. Cepheid. Xpert CT/NG Assay package insert. Vol. 301-0234, Rev B (Cepheid, 2013).
2. Hologic. Aptima Combo 2 Assay package insert. Vol. 201798 Rev D (2012).
3. BD. BD ProbeTec *Chlamydia trachomatis* (CT) Qx Amplified DNA Assay package insert. Vol. 8081408 (2010).
4. BD. BD ProbeTec *Neisseria gonorrhoeae* (GC) Qx Amplified DNA Assay package insert. Vol. 8081409 (2010).
5. WHO. Global Incidence and prevalence of selected curable sexually transmitted infections. (WHO Department of Reproductive Health and Research, 2008).
6. CDC. Sexually Transmitted Disease Surveillance. (2013).
7. Liu, B.*, et al.* Chlamydia and gonorrhoea infections and the risk of adverse obstetric outcomes: a retrospective cohort study. *Sex Transm Infect* **89**, 672-678 (2013).
8. O'Brien, J.P., Goldenberg, D.L. & Rice, P.A. Disseminated gonococcal infection: a prospective analysis of 49 patients and a review of pathophysiology and immune mechanisms. *Medicine* **62**, 395-406 (1983).
9. Westrom, L., Joesoef, R., Reynolds, G., Hagdu, A. & Thompson, S.E. Pelvic inflammatory disease and fertility. A cohort study of 1,844 women with laparoscopically verified disease and 657 control women with normal laparoscopic results. *Sex Transm Dis* **19**, 185-192 (1992).
10. Rottingen, J.A., Cameron, D.W. & Garnett, G.P. A systematic review of the epidemiologic interactions between classic sexually transmitted diseases and HIV: how much really is known? *Sex Transm Dis* **28**, 579-597 (2001).
11. Deguchi, T., Yasuda, M. & Ito, S. Management of pharyngeal gonorrhea is crucial to prevent the emergence and spread of antibiotic-resistant *Neisseria gonorrhoeae*. *Antimicrob Agents Chemother.* **56**, 4039-4040 (2012).
12. Workowski, K.A. & Bolan, G.A. Sexually transmitted diseases treatment guidelines, 2015. *MMWR Recomm Rep* **64**, 1-137 (2015).
13. Zakher, B., Cantor, A.G., Pappas, M., Daeges, M. & Nelson, H.D. Screening for gonorrhea and chlamydia: a systematic review for the U.S. Preventive Services Task Force. *Ann Intern Med* **161**, 884-893 (2014).
14. Ota, K., Tamari, I. & Smieja, M. Detection of *Neisseria gonorrhoeae* and *Chlamydia trachomatis* in pharyngeal and rectal specimens using the BD ProbeTec ET system, the Gen-Probe Aptima Combo 2 assay and culture. *Sexually Transmitted Infections* **85**, 182-186 (2009).
15. Schachter, J., Moncada, J. & Liska, S. Nucleic acid amplification tests in the diagnosis of chlamydial and gonococcal infections of the oropharyngx and rectum in men who have sex with men. *Sexually Transmitted Diseases* **35**, 637-642 (2008).
16. Trebach, J.D., Chaulk, C.P., Page, K.R., Tuddenham, S. & Ghanem, K.G. *Neisseria gonorrhoeae* and *Chlamydia trachomatis* among women reporting extragenital exposures. *Sex Transm Dis* **42**, 233-239 (2015).
17. Bachmann, L., Johnson, R. & Cheng, H. Nucleic acid amplications test for diagnosis of *Neisseria gonorrhoeae* and *Chlamydia trachomatis* rectal infections. *Journal of Clinical Microbiology* **48**, 1827-1832 (2010).
18. Cosentino, L.A.*, et al.* Use of nucleic acid amplification testing for diagnosis of anorectal sexually transmitted infections. *J Clin Microbiol* **50**, 2005-2008 (2012).

19. Geelen, T.H.*, et al.* Performance of cobas(R) 4800 and m2000 real-time assays for detection of *Chlamydia trachomatis* and *Neisseria gonorrhoeae* in rectal and self-collected vaginal specimen. *Diagn Microbiol Infect Dis* **77**, 101-105 (2013).
20. Moncada, J., Schachter, J., Liska, S., Shayevich, C. & Klausner, J.D. Evaluation of self-collected glans and rectal swabs from men who have sex with men for detection of *Chlamydia trachomatis* and *Neisseria gonorrhoeae* by use of nucleic acid amplification tests. *J Clin Microbiol* **47**, 1657-1662 (2009).
21. Perry, M.D., Jones, R.N. & Corden, S.A. Is confirmatory testing of Roche cobas 4800 CT/NG test *Neisseria gonorrhoeae* positive samples required? Comparison of the Roche cobas 4800 CT/NG test with an opa/pap duplex assay for the detection of *N gonorrhoeae*. *Sex Transm Infect* **90**, 303-308 (2014).
22. Harryman, L.*, et al.* Comparative performance of culture using swabs transported in Amies medium and the Aptima Combo 2 nucleic acid amplification test in detection of Neisseria gonorrhoeae from genital and extra-genital sites: a retrospective study. *Sex Transm Infect* **88**, 27-31 (2012).
23. Pope, C.F.*, et al.* Positive predictive value of the Becton Dickinson VIPER system and the ProbeTec GC Q x assay, in extracted mode, for detection of Neisseria gonorrhoeae. *Sex Transm Infect* **86**, 465-469 (2010).
24. Valencia, F., Bufton, K. & Loeffelholz, M. Performance of the ProbeTec *Chlamydia trachomatis* and *Neisseria gonorrhoeae* Qx Assays on Throat and Rectal Swab Specimens. in *American Society of Microbiology* (Denver, Colorado, 2013).
25. Gratzer, B., Hattan, A., Nawrocki, J. & Janda, W. Performance of the BD ProbeTecTM Qx Amplified DNA Assay on the BD ViperTM System with XTRTM Technology for Non-Genital Neisseria Gonorrhoeae Screening Among MSM. in *National STD Prevention Conference*, Vol. P181 (Minneapolis, MN, 2012).
26. Pol, B.V.d., Williams, J., Pantone, A. & Arno, J. Evaluation of Three Diagnostic Systems for Detection of *Chlamydia trachomatis* and *Neisseria gonorrhoeae* from Oral Specimens. in *International Society for Sexually Transmitted Diseases* (Vienna, Austria, 2013).
27. Goldenberg, S., Finn, J., Sedudzi, E., White, J. & Tong, C. Performance of the GenExpert CT/NG Assay compared to that of the Aptima AC2 Assay for Detection of Rectal *Chlamydia trachomatis* and *Neisseria gonorrhoeae* by use of Residual Aptima Samples. *Journal of Clinical Microbiology* **50**, 3867-3869 (2012).
28. Cosentino, L.*, et al.* A Validation Study of the Cepheid Xpert CT/NG for detecting *Chlamydia trachomatis* and *Neisseria gonorrhoeae* in Rectal Samples. *Sex Transm Infect* **91**, A1-A258 (2015).
29. CDC. Recommendations for the Laboratory-Based Detection of Chlamydia trachomatis and Neisseria gonorrhoeae. (2014).
30. Gen-Probe. APTIMA Chlamydia trachomatis Assay. Vol. 501799 Revision D (San Diego, 2012).
31. Gen-Probe. APTIMA Neisseria gonorrhoeae Assay. Vol. 502486 Revision A 73 (San Diego, 2011).
32. Boyadzhyan, B., Yashina, T., Yatabe, J.H., Patnaik, M. & Hill, C.S. Comparison of the APTIMA CT and GC assays with the APTIMA combo 2 assay, the Abbott LCx assay, and direct fluorescent-antibody and culture assays for detection of Chlamydia trachomatis and Neisseria gonorrhoeae. *J Clin Microbiol* **42**, 3089-3093 (2004).
33. Chernesky, M.A.*, et al.* Ability of new APTIMA CT and APTIMA GC assays to detect Chlamydia trachomatis and Neisseria gonorrhoeae in male urine and urethral swabs. *J Clin Microbiol* **43**, 127-131 (2005).

34. Moncada, J., Donegan, E. & Schachter, J. Evaluation of CDC-recommended approaches for confirmatory testing of positive Neisseria gonorrhoeae nucleic acid amplification test results. *J Clin Microbiol* **46**, 1614-1619 (2008).
35. Tabrizi, S.N.*, et al.* Evaluation of six commercial nucleic acid amplification tests for detection of Neisseria gonorrhoeae and other Neisseria species. *J Clin Microbiol* **49**, 3610-3615 (2011).
36. FDA. Establishing the performance characteristics of *in vivo* diagnostics devices for *Chlamydia trachomatis* and/or *Neisseria gonorrhoeae*: screening and diagnostic testing. (2011).

APPENDIX A - Schedule of Events

	Enrollment
**Clinical Evaluations**	
Review of eligibility	X
Verbal consent	X
Baseline CRFs	X
**Collection of Specimens**	
Rectal swabs	X
Pharyngeal swabs	X

Antibacterial Resistance Leadership Group (ARLG)

Performance of Nucleic Acid Amplification Tests for the Detection of *Neisseria gonorrhoeae* and *Chlamydia trachomatis* in Extragenital Sites (pNAAT)

Protocol Number:

ARLG_pNAAT-Yr3

Summary of Changes Protocol Version 2.0

August 1st, 2016

Section	Changes	Rationale
**Entire Protocol**	The protocol has been revised to replace all references to BD's ProbeTec Q with Abbott's RealTime CT/NG as the third platform	Becton Dickenson (BD) informed DCRI that the company no longer wished to participate in the research study by providing its ProbeTec Q *Chlamydia trachomatis* and *Neisseria gonorrhoeae* Amplified DNA Assays as the third testing platform. Therefore, Duke will be purchasing the Abbott RealTime *Chlamydia trachomatis* and *Neisseria gonorrhoeae* assays for use in this research study as the third testing platform.

Antibacterial Resistance Leadership Group (ARLG)

Performance of Nucleic Acid Amplification Tests for the Detection of *Neisseria gonorrhoeae* and *Chlamydia trachomatis* in Extragenital Sites (pNAAT)

Protocol Number:

ARLG_pNAAT-Yr3

Summary of Changes Protocol Version 3.0

September 5th, 2017

Section	Changes	Rationale
**ARLG Protocol Team Roster**	Replace Project Leader Elizabeth Petzold with Zoe Sund	Update current protocol operations team
**2.3**	Modified wording of Outcome Measures for: Combo 2 Assay, Abbot RealTime CT/NG assay, and Tiebreaker assays	Changes in wording were made to coincide with the results terminology utilized in the package inserts from the platforms tested and confirmed by the manufacturers
**2.3 (Table 2)**	Changed wording from NR/+/NR Status from Indeterminate to Invalid remove from analysis	Changes in wording were adopted to clarify how this result would be reported
**6.3**	Added footnote [35]	Inadvertently left out in Protocol Version 2
**6.6 (Tables 4 and 5)**	Updated wording of tables and related protocol text	Changes made to be consistent with terminology that was altered in section 2.3

Antibacterial Resistance Leadership Group (ARLG)

Performance of Nucleic Acid Amplification Tests for the Detection of *Neisseria gonorrhoeae* and *Chlamydia trachomatis* in Extragenital Sites
(pNAAT)

Protocol Number:

ARLG_pNAAT-Yr3

Summary of Changes Protocol Version 4.0

December 4th, 2017

Section	Changes	Rationale
**Protocol Synopsis**	Increased enrollment number to “up to 3,000” and study duration to “ approximately 12 - 24 months”	Slightly lower than anticipated infection rate has necessitated the need for increased population up to 3,000 participants. Increasing population includes the potential for increased study duration.
**3.0**	Modified wording to reflect the increased enrollment number and removed a reference to the study duration	Consistent with increased enrollment number. Study duration addressed elsewhere in protocol.
**4.1**	Changed enrollment number to “up to 3000” participants	Consistent with increased enrollment number

Antibacterial Resistance Leadership Group (ARLG)

Performance of Nucleic Acid Amplification Tests for the Detection of *Neisseria gonorrhoeae* and *Chlamydia trachomatis* in Extragenital Sites (pNAAT)

Protocol Number:

ARLG_pNAAT-Yr3

Funding Sponsor:

National Institute of Allergy and Infectious Diseases (NIAID)

Funding Mechanism: 5UM1AI104681

**Protocol Date:**	December 4th, 2017
**Protocol Version:**	4.0
**Principal Investigator**	Dr. Jeffrey D. Klausner, MD, MPH

Signature Page

The signature below documents the review and approval of this protocol and provides the necessary assurances that this study will be conducted according to the protocol, including all statements regarding confidentiality, and according to national, regional, and local legal and regulatory requirements.

____
Site Principal Investigator Name (Print)

____ ___________________________
Signature Date

ARLG GC Protocol Team Roster

All questions concerning this protocol should be sent via email to arlg.gc@mc.duke.edu. The appropriate team member will respond to questions via email, generally within 24 hours (Monday-Friday).

Protocol Chair

Jeffrey D. Klausner, MD, MPH
Professor of Medicine and Public Health
University of California, Los Angeles
10833 Le Conte Avenue
Los Angeles, CA 90095
Phone: 310-267-0409
Email: JDKlausner@mednet.ucla.edu

Project Leader

Zoe Sund
Duke Clinical Research Institute
2400 Pratt Street
Durham NC 27705
Phone: 919-428-1007
Email: zoe.sund@duke.edu

Statisticians

Scott Evans, PhD
Harvard School of Public Health FXB 513
651 Huntington Avenue
Boston, MA 02115-6017
Phone: 617-432-2998
Email: evans@sdac.harvard.edu

Lauren Komarow, MSc
Harvard School of Public Health FXB 549A
651 Huntington Avenue
Boston, MA 02115-6017
Phone: 617-432-3233
Email: lkomarow@sdac.harvard.edu

Thuy Tran, MSc
Harvard School of Public Health FXB 608
651 Huntington Avenue
Boston, MA 02115-6017
Phone: 617-432-7523
Email: ttran@sdac.harvard.edu

Protocol Clinicians

Sarah Doernberg, MD, MAS
Division of Infectious Diseases
University of California, San Francisco
513 Parnassus Ave, Box 0654
San Francisco, CA 94143
Phone: 415-502-5548
Email: sarah.doernberg@ucsf.edu

Table of Contents

LIST OF ABBREVIATIONS AND DEFINITIONS 6

1.0 BACKGROUND AND SCIENTIFIC RATIONALE 9

1.1 BACKGROUND INFORMATION 9
1.2 SCIENTIFIC RATIONALE 9

2.0 OBJECTIVES 11

2.1 PRIMARY OBJECTIVE 11
2.2 SECONDARY OBJECTIVES 11
2.3 OUTCOME MEASURES 11

3.0 STUDY DESIGN 14

3.1 STUDY DESIGN 14
3.2 INCLUSION CRITERIA 15
3.3 EXCLUSION CRITERIA 15

4.0 STUDY PROCEDURES 16

4.1 RECRUITMENT PLAN 16
4.2 SCREENING 16
4.3 ENROLLMENT 16
4.6 FOLLOW-UP VISIT 17

5.0 DEVICE MONITORING 17

5.1 SPECIMEN COLLECTION DEVICE MONITORING 17
5.2 LABORATORY DEVICE MONITORING 17

6.0 STATISTICS 18

6.1 STUDY DESIGN 18
6.2 ENDPOINTS 18
6.3 SAMPLE SIZE 18
6.4 RANDOMIZATION OF SWABS 19
6.5 MONITORING 19
6.6 ANALYSES 20

7.0 QUALITY CONTROL 23

7.1 QUALITY CONTROLS AND STUDY MONITORING 23
7.2 SOURCE DOCUMENTS AND ACCESS TO SOURCE DATA 23

8.0 ETHICS / PROTECTION OF HUMAN SUBJECTS 23

8.1 ETHICAL STANDARDS 23
8.2 INSTITUTIONAL REVIEW BOARD AND INFORMED CONSENT 23
8.3 SUBJECT CONFIDENTIALITY 23
8.4 SPECIMEN HANDLING 24

9.0 DATA HANDLING AND RECORD KEEPING **24**
9.1 DATA COLLECTION 24
9.2 INSPECTION OF RECORDS 24
9.3 RETENTION OF RECORDS 24
9.4 CONFIDENTIALITY 25
10.0 PUBLICATION POLICY **25**
APPENDIX A - SCHEDULE OF EVENTS **30**

List of Abbreviations and Definitions

ARLG	Antibacterial Resistance Leadership Group
ASIS	Anatomic Site Infection Status
CDC	Center for Disease Control and Prevention
CFR	Code of Federal Regulations
CT	*Chlamydia trachomatis*
DCRI	Duke Clinical Research Institute
DNA	Deoxyribonucleic acid
Equivocal	Final equivocal result from nucleic acid amplification test, as determined by manufacturer's guidance. This means the test was run and the result was neither positive nor negative
FDA	Food and Drug Administration
HIV	Human immunodeficiency virus
ICMJE	International Committee of Medical Journal Editors
ID	Identification
IFU	Instructions for Use
Indeterminate	Anatomic site infected standard cannot be determined as positive or negative
IRB	Institutional Review Board
ISRC	Independent Study Review Committee
LGBT	Lesbian, gay, bisexual and transgender
NAAT	Nucleic acid amplification test
Negative (-) NAAT reading	Result from the nucleic acid amplification test is negative
NR	This means no test was run and there is no result
NPA	Negative percent agreement
NPV	Negative predictive value
NG	*Neisseria gonorrhoeae*
NIH	National Institutes of Health
OHRP	Office of Human Research Protections
Positive (+) NAAT reading	Result from the nucleic acid amplification test is positive
PPA	Positive percent agreement
PPV	Positive predictive value
PI	Principal Investigator
RNA	Ribonucleic acid

rRNA	Ribosomal ribonucleic acid
SDMC	Statistical and Data Monitoring Center
STD	Sexually transmitted diseases
WHO	World Health Organization

Protocol Synopsis

Protocol Title:	Performance of Nucleic Acid Amplification Tests for the Detection of *Neisseria gonorrhoeae* and *Chlamydia trachomatis* in Extragenital Sites
Study Design:	A cross-sectional, single visit study to evaluate the diagnostic accuracy of nucleic acid amplification tests for detection of *Neisseria gonorrhoeae* and *Chlamydia trachomatis* from pharyngeal and rectal sites
Primary Study Objectives:	For each nucleic acid amplification assay, estimate the positive percent agreement (PPA) and negative percent agreement (NPA) for detecting:<br>• *Neisseria gonorrhoeae* in rectal swabs<br>• *Neisseria gonorrhoeae* in pharyngeal swabs<br>• *Chlamydia trachomatis* in rectal swabs<br>• *Chlamydia trachomatis* in pharyngeal swabs
Study Population	Patients presenting to an outpatient clinic for sexually transmitted disease (STD) testing for *Neisseria gonorrhoeae* and *Chlamydia trachomatis* infections
Number of subjects	Up to 3,000
Study duration	Approximately 12 to 24 months
Number of sites	Up to 10 clinical sites (as needed)
Clinical Samples	Pharyngeal and rectal swabs

1.0 Background and Scientific Rationale

1.1 Background Information

Nucleic acid amplification tests (NAATs) have become the gold standard for diagnosing *Neisseria gonorrhoeae* (NG) and *Chlamydia trachomatis* (CT) infections in the urogenital tract but are not currently approved by the Food and Drug Administration (FDA) for use in extragenital sites, including the pharynx and the rectum.

The goal of this study is to determine the diagnostic accuracy of the following 3 NAAT platforms for the detection of NG and CT from the pharynx and the rectum:

- Xpert® CT/NG Assay [Cepheid] [1]
- Combo 2 ® Assay [Hologic] [2]
- Abbott RealTi*me* *Chlamydia trachomatis/Neisseria gonorrhoeae* assay [Abbott] [3]

The assays being evaluated are intended for the direct qualitative detection of *Chlamydia trachomatis* and/or *Neisseria gonorrhoeae* in rectal or pharyngeal clinician-collected swabs from symptomatic or asymptomatic male or female patients.

1.2 Scientific Rationale

Infections due to NG and CT are a major public health threat. The World Health Organization (WHO) estimated more than 100 million new worldwide cases for each of NG and CT in 2008.[4] In the US alone, there were 333,000 NG and 1.4 million CT genitourinary infections in the year 2013.[5] Over the past 15 years, researchers have demonstrated an important burden of NG and CT infection in extragenital sites - the pharynx and rectum. Most CT and NG infections are asymptomatic, but infection can lead to serious sequelae, including infertility, chronic pelvic pain, adverse obstetrical outcomes, increased risk of human immunodeficiency virus (HIV) acquisition, and disseminated infection.[6-9] In addition to threats to individual health, there has been growing concern over antibiotic resistance, and in 2013, the US Centers for Disease Control and Prevention (CDC) classified drug-resistant NG as one of the three urgent-level resistant bacteria. Improved detection of extragenital NG is thought to be a crucial component of adequate treatment and thus prevention of further resistance.[10]

Currently, the CDC recommends the use of NAATs for NG and CT screening and diagnosis in the genitourinary tract due to superior sensitivity compared to traditional culture methods.[11] The sensitivity and specificity of commercially available NAATs for the detection of genitourinary NG and CT infection is estimated to be between 90-100%.[12] CDC also recommends the use of NAATs for extragenital sites.[11] Due to widespread use and interest in these tests for extragenital NG and CT infections, multiple studies have examined the test characteristics of NAAT tests in comparison to culture and to additional NAAT platforms (see Table 1). For diagnosis of CT, reported sensitivities are 80-100% (pharyngeal) and 46-100% (rectal), with significant variation by platform tested.[13-19] Reported specificities are >99% for pharyngeal site and 89-100% for the rectum. For diagnosis of NG, reported sensitivities are 72-100% (pharyngeal) and 75-100% (rectal), again with variation by platform.[13,14,16-18,20,21] Reported specificities are 72-100% (pharyngeal) and 95-100% (rectal).

Table 1. Prior Studies of extragenital testing for chlamydia and gonorrhea.

**Screening test**	**Sensitivity/specificity* by specimen type (%)**	
	**Pharyngeal**	**Rectal**
Neisseria gonorrhoeae		
**Hologic Gen-Probe Aptima Combo 2**		
Bachmann LH *et al,* 2010[16]	100 / 96.2	100 / 95.5
Cosentino LA *et al,* 2012[17]		76 / 100
Moncada J *et al,* 2009[19]		78.3 / 99.8
Ota KV et al, 2009[13]	95 / 99.6	100 / 100
Schachter J *et al*, 2008[14]	84.3 / 99.4	93.2 / 99.7
**Abbott RealTi*me* CT/NG**		
Walsh A ***et al***, 2011[22]	100/100	100/100
**Cepheid Xpert® CT/NG Assay**		
Goldenberg SD *et al*., 2012[23]		91.1 / 100
Cosentino LA *et al*, 2015[24]		100 / 100
Chlamydia trachomatis		
**Hologic Gen-Probe Aptima Combo 2**		
Bachmann LH *et al,* 2010[16]		100 / 88.8
Cosentino LA *et al,* 2012[17]		100 / 99.8
Moncada J *et al,* 2009[19]		71.2 / 99
Ota KV *et al*, 2009[13]	100 / 99.2	100 / 98.7
Schachter J *et al,* 2008[14]	100 / 99.6	93.5 / 97.7
**Abbott RealTi*me* CT/NG**		
No studies available		
**Cepheid Xpert® CT/NG Assay**		
Goldenberg SD *et al*., 2012[23]		86 / 99.2
Cosentino LA *et al*, 2015[24]		96.6 / 99.7

*Sensitivity and specificity as reported by authors, each using unique definitions for the reference standard. As discussed below, we will be using positive percent agreement (PPA) and negative percent agreement (NPA) for these calculations.

Despite the CDC recommendations, at this time, there are no FDA-cleared commercial NAAT tests for the detection of pharyngeal and rectal NG or CT infection. The FDA-clearance of such assays would increase the availability and uptake and subsequently lead to improved screening and enhanced control of NG and CT extragenital infections. The current proposed study will help provide the data required for FDA consideration to clear such assays for marketing.

Because of the multi-site nature of the ARLG, its strong laboratory network and inclusion of investigators with expertise in NG and CT detection and treatment, the ARLG is well-positioned to conduct such a study. Current commercial assays are "dual" assays and include targets for both NG and CT, so the goal of this study will be to evaluate the diagnostic accuracy for detection of both organisms at extragenital sites.

The molecular targets of the three NAAT platforms that will be tested differ, which will allow for comparative evaluation.[25]

- Xpert® CT/NG Assay (Cepheid) is a combination test that uses a real-time PCR technique to detect two noncontiguous chromosomal DNA regions from NG (NG2 and NG4) - both of which must be positive to yield a positive result - and one chromosomal DNA target from CT (CT1) [1]

- Combo 2 ® Assay (Hologic) is a combination test that utilizes target capture, transcription mediated amplification, and dual kinetic assay to detect regions of the ribosomal RNA (rRNA) from the 16S rRNA of GC and the 23S rRNA from CT using labeled DNA probes [2]
- Abbott Real*Time Chlamydia trachomatis* and *Neisseria gonorrhoeae* assay (Abbott) is a combination test that uses a real-time PCR assay to detect a highly-conserved region within the *Opa* gene of NG and two distinct regions within the CT cryptic plasmid DNA [3]

Additional testing using the Aptima® *Chlamydia trachomatis* assay (Hologic)[26] and/or the Aptima® *Neisseria gonorrhoeae* assay (Hologic)[27] will be performed in cases of discordant results (see Section 3.5). This test utilizes target capture, transcription mediated amplification, and hybridization protection assays to identify the presence of RNA from the organism of interest in the clinical sample. The targets from the 16S rRNA for both GC and CT are different than those used in the Combo 2® Assay.[2,26,27] Though the diagnostic accuracy of these assays for extragenital infections has not previously been evaluated, studies evaluating the performance of these assays for confirmation of other NAATs suggest excellent concordance.[28-31]

2.0 Objectives

2.1 Primary Objective

For each NAAT, estimate the PPAs and NPAs for detecting:

- *Neisseria gonorrhoeae* in rectal swabs
- *Neisseria gonorrhoeae* in pharyngeal swabs
- *Chlamydia trachomatis* in rectal swabs
- *Chlamydia trachomatis* in pharyngeal swabs

2.2 Secondary Objectives

2.2.1 Global analyses:

For each NAAT, positive predictive values (PPVs), negative predictive values (NPVs), positive likelihood ratios, negative likelihood ratios, and sensitivity analyses will be calculated for detecting:

- *Neisseria gonorrhoeae* in rectal swabs
- *Neisseria gonorrhoeae* in pharyngeal swabs
- *Chlamydia trachomatis* in rectal swabs
- *Chlamydia trachomatis* in pharyngeal swabs

2.2.2 Subgroup analyses:

For each NAAT, to estimate the PPAs, NPAs, PPVs, and NPVs for detection of NG and CT from rectal and pharyngeal swab specimens by sex and by anatomic site-specific symptom status.

2.3 Outcome Measures

The anatomic site infected status (ASIS) will be determined for each anatomic site (pharyngeal and rectal) and each organism (NG and CT), as shown below in Table 2.

Possible ASIS outcomes are:

- Infected
- Not infected
- Indeterminate
- Invalid, exclude from analysis

The anatomic site is considered to be infected if both of the reference test results are positive. The anatomic site is considered to be not infected when both reference test results are negative. If there is discordance between the reference tests, an additional NAAT will be done as a tiebreaker. In this case, agreement of 2/3 of the reference NAATs will determine the ASIS. If two tests are equivocal or one equivocal and one not run, the third test result will stand as the ASIS if positive or negative. If two tests are not run, the ASIS will be considered invalid and will be excluded from the analysis. All possible combinations are shown in Table 2. The tiebreaker test will be run by the lab if any NAAT is not concordant with the others and interpreted only in the case of discordant results between the two planned reference tests for each assay. As the tiebreaker test is not a combination test, the tiebreaker will only be run for the organism with disagreement (e.g. if NG disagrees and CT agrees, the tiebreaker will only be run for NG).

To determine the ASIS, the test result for each respective site (pharyngeal or rectal) and each organism (NG or CT) for each NAAT platform will be used. Possible outcomes for each assay are:

- **Xpert® CT/NG Assay (Cepheid)**[1]: Not detected, detected, invalid (sample processing control or sample adequacy control failed), error (probe check control failed), or no result (insufficient data was collected, e.g. test aborted). Initial invalid, error, or no result tests will be repeated. If the repeat test returns invalid, error, or no result, the final result will be considered an invalid and will be categorized as <u>no result (NR)</u> for the ASIS determination and statistical analyses below. If the repeat test returns not detected (negative) or detected (positive), this will be the result used for the ASIS determination and statistical analyses.

- **Combo 2 ® Assay (Hologic)**[2]: Negative, positive, equivocal (result between positive and negative), invalid (run status is FAIL or other technical failure), or error (sample was not tested due to an error detected by the instrument). Initial equivocal, invalid, and error test results will be repeated. If the repeat test result returns equivocal, the final test result will be considered an <u>equivocal test result</u> for the ASIS determination and statistical analyses below. If the repeat test result returns invalid or error, the final test result will be categorized as <u>equivocal</u> if the initial test was equivocal and as <u>no result (NR)</u> if the initial test was invalid or error for the ASIS determination and statistical analyses below. If the repeat test returns negative or positive, this will be the result considered for the ASIS determination and statistical analyses.

- **Abbott RealTime CT/NG assay (Abbott)[3]:** For NG, possible results are positive (detected, with cycle number less than or equal to the assay cut-off), negative (no evidence of amplification or cycle number greater than the assay cut-off), and error. An equivocal interpretation does not apply. For CT, possible results are positive (detected, with cycle number less than or equal to the assay cut-off), negative (no evidence of amplification), equivocal (cycle number beyond the assay cut-off), and error. A sample with initial interpretation of error (both CT and NG) or equivocal (CT only) will be retested. If the repeat test returns negative or positive, this will be the result considered for the ASIS determination and statistical analyses. If the repeat

test result is equivocal (CT only), the final test result will be considered equivocal for the ASIS determination and statistical analyses below. If the repeat test result is error, the final test result will be categorized as no result (NR) for the ASIS determination and statistical analyses below if the initial test result was error and as equivocal if the initial test result was equivocal (CT only).

- **Tiebreaker assays: Aptima® *Chlamydia trachomatis* assay (Gen-Probe)[26] and the Aptima® *Neisseria gonorrhoeae* assay (Gen-Probe)[27]**: Negative, positive, equivocal (result between negative and positive ranges), invalid (run status is FAIL or other technical failure), or error (sample was not tested due to an error detected by the instrument). Initial equivocal, invalid and error test results will be repeated. If the repeated test result is equivocal, it will be considered an equivocal test result for the ASIS determination and statistical analyses below. If the repeat test result returns invalid or error, the final test result will be categorized as equivocal if the initial test was equivocal and as no result (NR) if the initial test was invalid or error for the ASIS determination and statistical analyses below. If the repeat test returns negative or positive, this will be the result considered for the ASIS determination and statistical analyses.

Table 2. Determination of the Anatomic Site Infected Standard

Comparator NAAT 1 Result	Comparator NAAT 2 Result	Tiebreaker NAAT Result	Anatomic Site Infection status
+	+	Not indicated	Infected
+	-	+	Infected
+	E*	+	Infected
+	NR**	+	Infected
+	-	-	Not infected
+	-	E	Indeterminate
+	-	NR	Indeterminate
+	E	-	Indeterminate
+	E	E	Infected
+	E	NR	Infected
+	NR	-	Indeterminate
+	NR	E	Infected
+	NR	NR	Invalid, remove from analysis
-	-	Not indicated	Not infected
-	+	-	Not infected
-	E	-	Not infected
-	NR	-	Not infected
-	+	+	Infected
-	+	E	Indeterminate
-	+	NR	Indeterminate
-	E	+	Indeterminate
-	E	E	Not infected

-	E	NR	Not infected
-	NR	+	Indeterminate
-	NR	E	Not infected
-	NR	NR	Invalid,<br>remove from analysis
E	+	+	Infected
E	-	-	Not infected
E	+	-	Indeterminate
E	+	E	Infected
E	+	NR	Infected
E	-	+	Indeterminate
E	-	E	Not infected
E	-	NR	Not infected
E	NR	+	Infected
E	NR	-	Not infected
E	NR	E	Indeterminate
E	NR	NR	Invalid,<br>remove from analysis
NR	+	+	Infected
NR	-	-	Not infected
NR	NR	Not indicated	Invalid,<br>remove from analysis
NR	+	-	Indeterminate
NR	+	E	Infected
NR	+	NR	Invalid,<br>remove from analysis
NR	-	+	Indeterminate
NR	-	E	Not infected
NR	-	NR	Invalid,<br>remove from analysis
NR	E	+	Infected
NR	E	-	Not infected
NR	E	E	Indeterminate
NR	E	NR	Invalid,<br>remove from analysis

*E **=** equivocal result
******NR = no result. This can occur either because the test result was invalid or because the test could not be run (e.g. too little sample, improperly shipped, no sample received).

3.0 Study Design

3.1 Study Design

This will be a cross-sectional, single visit study evaluating the performance of three commercial NAATs to detect NG and CT in the rectum and pharynx.[1,2,32,33] A fourth NAAT is being used as a tiebreaker.[26,27] We are not evaluating the performance of this fourth NAAT.

The study will include specimens collected from adult subjects seeking sexually transmitted disease (STD) testing at the participating clinics, which include clinics focused on sexually transmitted diseases, women's health, student health, family planning clinics, and clinics specializing in lesbian, gay, bisexual, and transgender (LGBT) health. Potential subjects will be identified, assessed for eligibility and approached for informed consent. Both symptomatic and asymptomatic individuals will be included in the study population.

The study will enroll up to 3,000 subjects who will have four pharyngeal and four rectal swabs collected as part of a one-time study visit. Each manufacturer will have specific collection kit and transport media. Additional data collection will include, but is not limited to, eligibility screening questions, gender, age, and patient report of relevant pharyngeal, rectal, or other relevant symptoms, and swab collection completeness. Subjects will be classified as symptomatic or asymptomatic for each anatomic site. Results of testing at other anatomic sites and results of clinical testing will not be available.

Each swab will be used for a specific NAAT, which will be run at one of the two reference testing laboratories. The ASIS will be defined by the results of the two other nucleic acid amplification tests, and a tiebreaker test (if necessary), as shown in Table 2. Each anatomic site will be considered in isolation.

When accrual is complete, the study will be closed and an analysis plan will be enacted to finalize the database for analysis.

3.2 Inclusion Criteria

a) Attending a participating clinic for evaluation of STDs
b) ≥18 years of age at date of screening
c) Able and willing to provide informed consent
d) Willing to comply with study procedures, including collection of 4 swabs each from the pharynx and rectum for NG and CT testing

NOTE: Male, female, and transgender individuals will be included, and both symptomatic and asymptomatic individuals will be included.

3.3 Exclusion Criteria

a) Receipt of any systemic antibacterial drug in the past 14 days
b) Receipt of myelosuppressive chemotherapy in the past 30 days

3.4 Nucleic Acid Amplification Tests (NAATs)

This study will evaluate three distinct NAAT platforms. The selected platforms[25] are:

- Xpert® CT/NG Assay (Cepheid) uses a real-time PCR technique to detect two noncontiguous chromosomal DNA regions from NG (NG2 and NG4) - both of which must be positive to yield a positive result - and one chromosomal DNA target from CT (CT1). [1]

- APTIMA Combo 2® Assay (Hologic) utilizes target capture, transcription mediated amplification, and dual kinetic assay to detect regions of the rRNA from the 16S rRNA of GC and the 23S rRNA from CT using labeled DNA probes.[2]

- Abbott RealTi*me* *Chlamydia trachomatis* and *Neisseria gonorrhoeae* assay (Abbott) is a combination test that uses a real-time PCR assay to detect a highly-conserved region within the *Opa* gene of NG and two distinct regions within the CT cryptic plasmid DNA [3]

3.5 Tiebreaker Assay

In case of disagreement between reference NAAT results, a tiebreaker assay will be performed. This assay will use the Hologic Tigris platform with the Aptima® *Chlamydia trachomatis* assay and/or the Aptima® *Neisseria gonorrhoeae* assay (see Section 2.3). These tests utilize target capture, transcription mediated amplification, and hybridization protection assays to identify the presence of RNA from the organism of interest in the clinical sample. The targets from the 16S rRNA for both GC and CT are different than those used in the Combo 2® Assay.[26,27]

4.0 Study Procedures

4.1 Recruitment Plan

This study will enroll up to 3,000 participants who are seeking STD testing at one of the participating clinics. Potential subjects will be recruited without regard for risk behavior, symptom status, or gender. Participating clinics may include those designed for STD screening and management, family planning, student health, women's health, HIV management, and clinics focusing on the LGBT population.

4.2 Screening

Participants will be recruited from clinical sites performing routine STD testing. It is assumed that individuals presenting for routine STD testing at the participating clinics are at risk for such infections. Each person will undergo screening for eligibility and, if eligible, will be approached for oral informed consent.

4.3 Enrollment

Once enrolled, eight swabs will be taken: four swabs will be taken from the pharynx and four swabs will be taken from the rectum in addition to any swabs taken as part of routine clinical care. The swabs collected for routine clinical care will be taken first. The order of the research swabs will be randomized per subject to account for the possibility that the yield of the swab is affected by previous swabs at the same anatomic site. The swabs will be stored and transported per manufacturer guidelines. Results of those NAATs will not be reported back to the treating clinician for clinical purposes.

Data collection will include, but is not limited to, assessment of signs and symptoms of rectal and/or pharyngeal infection, as well as socio-demographic information such as age (not date of birth), race, ethnicity and gender, and swab collection completeness.

4.5 Incomplete Swab Collection

While required as an inclusion criteria for a potential subject to agree to both pharyngeal and rectal swabs, if only one anatomic site is swabbed after enrollment for whatever reason, and all four swabs are completed for that anatomic site, those swabs for that anatomic site will be included in the analysis. If fewer than four swabs at a site are completed, that anatomic site (and swab results) will be excluded from the analysis.

4.4 Subject Withdrawal and Replacement

Subjects may voluntarily withdraw their consent for study participation at any time and for any reason, without penalty. The primary reason for withdrawal from the study will be recorded on the Study Status case report form. Subjects who withdraw will not be replaced.

4.5 Study Discontinuation

This study may be terminated at any time by the Principal Investigator (PI) in consultation with the Antibacterial Resistance Leadership Group (ARLG) and/or the National Institutes of Health (NIH).

4.6 Follow-up Visit

There will not be a follow-up visit as part of this study. Subjects will continue with routine clinical care as directed by their medical providers.

4.7 Central Laboratory Procedures

There will be two reference testing laboratories, which will divide the processing and running of the NAATs for the study. Each clinical study site will be assigned to send swabs to one of the two laboratories. Swabs will be processed and run at the reference testing laboratories according to each manufacturer’s Instructions for Use (IFU). Initial equivocal, invalid, or otherwise undetermined results will be repeated per manufacturer’s guidelines before determining whether the result will be classified as positive, negative, equivocal, or no result. One of the reference testing laboratories will be responsible for running all of the tiebreaker assays, regardless of the location of original study site.

The reference testing laboratories will receive training for each of the study platforms from a representative from each of the manufacturers, in order to ensure adherence to the manufacturer’s IFU procedures.

5.0 Device Monitoring

5.1 Specimen Collection Device Monitoring

Any complications with the specimen collection process, including device failure, significant patient discomfort, or inability to collect specimen, will be reported on the specimen collection case report form.

5.2 Laboratory Device Monitoring

The reference testing laboratories will maintain a log of all unanticipated device-related complications leading to no test, such as absence of transport media, quantity not sufficient, specimen transport collection system damage or incorrect transport system.

6.0 Statistics

6.1 Study Design

This is a cross-sectional, single visit study to evaluate the diagnostic accuracy of NAATs for the detection of NG and CT from pharyngeal and rectal swab specimens. Study participants will have swabs collected from both the rectum and pharynx, following any specimens taken for clinically indicated testing. Each of the swabs from each anatomic location will be tested for both NG and CT using three different NAATs, with a 4th test collected and tested as a tiebreaker, if needed. As the tiebreaker is not a combination test, it will only be run for the organism with disagreement. Diagnostic accuracy will be calculated with respect to an anatomic site infected status (ASIS) reference standard described in Section 3.5. As the reference standard is imperfect, we will report positive percent agreement and negative percent agreement in place of sensitivity and specificity.

One potential limitation of this study is that all three of the tests in the reference standard are NAATs. However, all three tests use different nucleic acid targets (rRNA vs. DNA with different molecular primers targeting different aspects of the CT and NG genome [see Section 3.4]), different methods of target amplification (transcription-mediated amplification, polymerase chain reaction and strand displacement amplification) and different methods of target detection (hybrid capture, multi-color laser detection and fluorescent polarization). So while, theoretically, there may be correlation in the errors produced by the three tests, the varied molecular targets and the different methods of capture, amplification and detection should mitigate this concern.

6.2 Endpoints

- Infection status for *Neisseria gonorrhoeae* in the rectum as determined by each NAAT
- Infection status for *Neisseria gonorrhoeae* in the pharynx as determined by each NAAT
- Infection status for *Chlamydia trachomatis* in the rectum as determined by each NAAT
- Infection status for *Chlamydia trachomatis* in the pharynx as determined by each NAAT
- Anatomic Site Infection status as determined by the reference standard (as described in Section 2.3)

6.3 Sample Size

For each assay, PPA and NPA[35] will be estimated for each of three diagnostic tests defined by pathogen - anatomical site combinations:

- *Neisseria gonorrhoeae* in the rectum
- *Neisseria gonorrhoeae* in the pharynx
- *Chlamydia trachomatis* in the rectum
- *Chlamydia trachomatis* in the pharynx

Each pathogen/anatomic site combination will be evaluated by constructing 95% Score confidence interval estimates for PPA and NPA and evaluating whether 90% can be ruled out by the lower bound of the CI with reasonable confidence.

NPA is expected to be greater than PPA and there will be considerably more not-infected than infected anatomic site, implying that there will be greater power for evaluating NPA. Thus it will suffice to size the study to evaluate PPA.

Assuming that the true PPA is 96%,[13] then 157, 174, and 196 evaluable participants that are infected are needed to have 80%, 85%, and 90% power respectively to demonstrate a PPA of greater than 90%. Since all of the participants will contribute to the evaluation of all three pathogen-anatomical site diagnostic tests, it will suffice to size the study for the diagnostic test with the lowest expected prevalence (except CT in the pharynx). The study will be more than sufficiently sized for more prevalent disease.

It is expected that the disease prevalence of NG in the rectum, NG in the pharynx, and CT in the rectum will each be greater than 7.5% in the population under evaluation.[13,14,16-20] Table 3 summarizes the required number of participants to have 80% and 90% probabilities of obtaining the 157, 174, and 196 evaluable participants that are disease-positive to have 80%, 85%, and 90% power when the true prevalence of disease is 7.5%, 10%, and 12.5%.

Table 3: Sample size required number to have 80% and 90% probabilities of obtaining the 157, 174, and 196 evaluable participants that are disease-positive when the true prevalence of disease is 7.5%, 10%, and 12.5% in order to have 80%, 85%, and 90% power (two-sided alpha=0.05) to conclude that sensitivity is greater than 90% when the true sensitivity is 96%.

Prevalence of Disease Positive	Power	Required Disease Positive to Obtain Power	Required Sample Size to have 80% probability of obtaining required Disease positive participants	Required Sample Size to have 90% probability of obtaining required Disease positive participants
**7.5%**	**80%**	157	2250	2325
	**85%**	174	2475	2575
	**90%**	196	2775	2875
**10%**	**80%**	157	1700	1750
	**85%**	174	1860	1925
	**90%**	196	2100	2150
**12.5%**	**80%**	157	1350	1400
	**85%**	174	1485	1530
	**90%**	196	1665	1715

6.4 Randomization of Swabs

Randomization will be employed to ensure that the swab order distribution is the same for 4 tests, the three NAATs under investigation in the study and the tiebreaker test. SAS software (Cary, NC) will be used to generate permuted blocks with a block size of 24, to ensure that each NAAT and the tiebreaker test would have their swab distributions consisting of 1/4 being first swabs, 1/4 being second swabs, 1/4 being third swabs and 1/4 being fourth swabs.

6.5 Monitoring

The core protocol team will receive monthly reports monitoring study accrual, study conduct and completeness of data and swab collection.

The study will be reviewed by an independent statistician, who will not have an association with the protocol or device companies. The independent statistician will review the progress of the study.

Additionally, the study statisticians will prepare reports for the independent statistician that will include baseline summaries (demographics and symptom status) and will address issues concerning the viability and appropriate execution of the protocol such as accrual, endpoint evaluability, and frequency of tests with equivocal results, invalid results, or no results. Issues with specimen collection will be summarized. The independent statistician will also review and evaluate the rates of disease. Toxicity is not expected given the nature of the study.

The study cannot be stopped at the interim for reaching regulatory goals at this time to preserve error rates / coverage probability and ensure enough data for subgroup analyses. Since the trial cannot be stopped for attainment of the regulatory goal, no adjustment to confidence levels are necessary.

Monitoring design assumptions for appropriate study sizing

Infection rates will be evaluated to determine whether sample size adjustments are warranted. The sample size will not be adjusted based on the observed PPAs and NPAs, which will not be reviewed while the study is ongoing, but may be adjusted based only on the infection rates to ensure enough infected participants to estimate PPAs with desired precision. If infection is more prevalent than expected, a smaller sample size may be accepted. If however infection is rarer than anticipated, then increases to sample size will be considered. The infection rate will be evaluated approximately every 500 participants or every 3 months after the enrollment of the first participant, whichever comes first.

6.6 Analyses

6.6.1 Primary Analyses

The result for each test will be compared with the ASIS for that anatomic site and organism. For each diagnostic test, PPA and NPA will be estimated using 95% Score confidence intervals. Confidence intervals will be estimated using the Score method.[34]

In the primary analysis, we will follow the FDA guidance document for incorporating indeterminate ASIS or test results for the test under consideration that are equivocal.[34] Indeterminate ASIS results will be counted against the results for the test under consideration, as described in Tables 4 and 5. Tables 4 and 5 will be presented in the analysis report, showing all combinations of Infected/Indeterminate/Not Infected with all outcomes from the test under consideration (Positive/Equivocal/Negative/No result or Invalid)

If the test under consideration has “no result” because the test was not run and no attempt was made to test the sample, it will be excluded from the primary analysis.

Table 4. Use of the ASIS in calculation of the positive and negative percent agreement for the Aptima Combo 2® Assay (Hologic) and Abbott RealTime CT/NG assay (CT only).

	**ASIS**		
	Infected	Indeterminate*	Not infected

**Result of Test under Consideration****	Positive	A	D	G
	Equivocal	B	E	H
	Negative	C	F	I
	No result	Exclude from analysis		
	PPA	A / (A+B+C+F)		
	NPA			I / (G + H + I + D)

*The middle, indeterminate column will be included in the analyses as “Infected” or “Not infected” as described in the sensitivity analyses below.
**Per the ASIS definition in Section 2.3

Comprehensive sensitivity analyses will be conducted considering multiple scenarios. These include:

- Classify indeterminate tests on the basis of symptom status. Include indeterminate tests as infected if the participant is symptomatic in that compartment. Include indeterminate tests as not infected in the participant is asymptomatic in that compartment.
- Include all indeterminate tests as infected.
  - o PPA=(A+D) / (A+B+C+D+E+F)
  - o NPA=I / (G+H+I)
- Include all indeterminate tests as not infected.
  - o PPA=A / (A+B+C)
  - o NPA=(F+I) / (D+E+F+G+H+I)
- Considering indeterminate and equivocal test results as “missing”, with the assumption of missing at random, and model the missing results.
- Additional sensitivity analyses, including the use of latent class models, will be explored. Details will be in the statistical analysis plan.

Table 5. Use of the ASIS in calculation of the positive and negative percent agreement for the Xpert® CT/NG Assay (Cepheid) and Abbott RealTime CT/NG assay (NG only).

		**ASIS**		
		Infected	Indeterminate*	Not infected
**Result of Test under Consideration****	Positive/Detected	A	C	E
	Negative/Not detected	B	D	F
	No result	Exclude from analysis		
	PPA	A / (A+B+D)		
	NPA			F / (C+E+F)

*The middle, indeterminate column will be included in the analyses as “Infected” or “Not infected” as described in the sensitivity analyses below.
**Per the ASIS definition in Section 2.3

If the test under consideration has “no result” because the test was not run and no attempt was made to test the sample or because of invalid ASIS results it will be excluded from the primary analysis.

Comprehensive sensitivity analyses will be conducted. These include:

- Classify indeterminate tests on the basis of symptom status. Include indeterminate tests as Infected if the participant is symptomatic in that compartment. Include indeterminate tests as not infected in the participant is asymptomatic in that compartment.

- Include all indeterminate tests as infected
  - o PPA = (A+C) / (A+B+C+D)
  - o NPA = F / (E+F)
- Include all indeterminate tests as not infected
  - o PPA = A / (A+B)
  - o NPA = (D+F) / (C+D+E+F)
- Additional sensitivity analyses that consider indeterminate results as “missing” and utilize imputation strategies, and graphical techniques
- Additional sensitivity analyses, including the use of latent class models, will be explored. Details will be in the statistical analysis plan.

There are pros and cons to the manner in which indeterminates are handled and the resulting calculations of PPA and NPA. The primary definition is the most conservative but is biased downwards. If PPA > 90% under this scenario, then the conclusion of PPA > 90% is clear. The second and third sensitivity analyses count indeterminates (treat all as infected or not infected, respectively) but may not be conservative for calculations of PPA and NPA.

6.6.2 Secondary Analyses

Global Analyses

For each diagnostic test:

- Positive and negative predictive values (PPV and NPV) will be estimated. PPV and NPV will each be plotted as a function of prevalence (point estimates and 95% pointwise confidence bands)
- Positive and negative likelihood ratios will be estimated using 95% Score confidence intervals
- A sensitivity analysis will include calculating equivocal predictive values and likelihood ratios
- An additional sensitivity analysis will calculate PPV, NPV, and positive and negative likelihood ratios for the sensitivity analyses where indeterminates are counted as Infected and Not Infected

Subgroup Analyses

For each of the three diagnostic tests, subgroup analyses will be conducted for males, females, symptomatic participants, and asymptomatic participants by pathogen and anatomic site. The 95% Score confidence interval estimates of PPA and NPA will be displayed using forest plots (one plot per test). The 95% Score confidence interval estimates of positive and negative likelihood ratios will be displayed using forest plots (one plot per test). PPVs and NPVs will each be plotted as a function of prevalence (point estimates and pointwise confidence bands).

The sensitivity analyses where indeterminates are counted as Infected and Not Infected will be repeated for the four subgroup analyses.

Exploratory Analysis

Developing methods for diagnostic benefit:risk analyses (e.g., diagnostic yield) will be applied.

7.0 Quality Control

7.1 Quality Controls and Study Monitoring

The ARLG will provide direct access to the dataset for the purposes of monitoring and auditing by the DCRI, Harvard School of Public Health and inspection by local and regulatory authorities. The local site PI will ensure that study personnel are appropriately trained and applicable documentations are maintained.

The DCRI will implement a Quality Plan to ensure that protocol training, data quality and data security are being undertaken.

7.2 Source Documents and Access to Source Data

Source documents will not be removed from the sites. Study monitoring will occur remotely, unless there are quality or performance concerns at a site, in which case an on-site visit will occur.

8.0 Ethics / Protection of Human Subjects

8.1 Ethical Standards

The investigator will ensure that the study will be conducted in accordance with all applicable national, regional, and local regulations.

8.2 Institutional Review Board and Informed Consent

This protocol and any subsequent modifications must be reviewed and approved by the IRB responsible for oversight of the study, as listed on its FWA. To minimize risks associated with loss of privacy and loss of confidentiality, oral informed consent will be obtained from the subject. The informed consent process will describe the purpose of the study, the procedures to be followed, and the risks and benefits of participation. A copy of a patient information form may be given to the subject (per local policy).

8.3 Subject Confidentiality

Subjects will have code numbers and will not be identified by name. Subject confidentiality is strictly held in trust by the participating investigators, their staff, and the Sponsor(s) and their agents.

The study protocol, documentation, data, and all other information generated will be held in strict confidence. No information concerning the study or the data will be released to any unauthorized third party without prior written approval of the ARLG.

All information provided by the ARLG and all data and information generated by the participating clinical site as part of the trial will be kept confidential by the site PI and other study personnel. This information and data will not be used by the site PI or other study personnel for any purpose other than conducting the trial. These restrictions do not apply to: (1) information which becomes publicly available through no fault of the site principal investigator or other study personnel; (2) information which is necessary to

disclose in confidence to an IRB solely for the evaluation of the trial (3) information which is necessary to disclose in order to provide appropriate medical care to a study subject; or (4) study results which may be published as described in Section 10.

The study monitor or other authorized representatives of the sponsor may inspect all documents and records required to be maintained by the PI. The clinical study site will permit access to such records.

All laboratory specimens, case report form data, reports and other records will be identified only by a coded number to maintain subject confidentiality.

8.4 Specimen Handling

Specimens will be collected for this study and will be maintained by the ARLG at least until all companies have received their FDA approval for their assay.

Use of samples for future research will be part of the consent process. There will **not** be any opt-in/opt-out language. Samples may be shared with other investigators at other institutions. No human genetic tests will be performed on samples. Each sample will be encoded (labeled) only with a barcode and a unique tracking number to protect subject's confidentiality.

There are no benefits to subjects in the collection, storage and subsequent research use of specimens. Because the subject enrollment will be done anonymously, reports about future research done with subject's samples will NOT be reported into their health records.

9.0 Data Handling and Record Keeping

9.1 Data Collection

This study will use eClinical OS (eCOS), a Web-based e-CRF database used by the DCRI. The investigator's site staff who will be entering data will receive training on the system, after which each person will be issued a unique user identification ID and password.

For security reasons, and in compliance with regulatory guidelines, it is imperative that only the persons who own the user IDs and passwords access the system using their own unique access codes. Access codes are nontransferable. Site personnel who have not undergone training may not use the system and will not be issued a user ID and password until appropriate training is completed.

9.2 Inspection of Records

The DCRI will implement a Quality Plan to, at a minimum, ensure that activities proposed by the PI to ensure protocol training, data quality and data security are being undertaken.

Source Documents and Access to Source Data

Source documents will not be removed from the sites.

9.3 Retention of Records

Records will be maintained during the investigation and for a period of 2 years after the latter of the following dates: The date on which the investigation is terminated or completed, or the date that the records are no longer required for purposes of supporting a premarket approval application or a notice of completion of a product development protocol. Records may not be destroyed without the prior approval of the ARLG.

9.4 Confidentiality

The study protocol, documentation, data, and all other information generated by this study will be maintained in a secure manner and will be kept confidential as required by law.

The EDC database (eClinical OS data) used by the DCRI will be hosted by Merge which uses a Peak 10 datacenter located in Morrisville NC. Database access will be limited to study personnel who are issued a unique user identification and password. Data will be entered at each site by study personnel. No information concerning the study or the data will be released to any third party without prior written approval of the Sponsor. Study records may be reviewed in order to meet federal or state regulations. Reviewers may include the IRBs, the DCRI and the NIH.

10.0 Publication Policy

Following completion of the study, the investigator may publish the results of this research in a scientific journal under the oversight of the Publication Committee of the ARLG.

The ARLG Publication Committee comprises representatives of the network cores, thought-leaders, statistics and data management center (SDMC), and is responsible for generation and coordination of the publications that report scientific findings of the network. All public presentations (abstracts, manuscripts, slides and text of oral or other presentations, and text of any transmission through any electronic media) by participating investigators, participating institutions, SDMC, and ARLG that use ARLG data and are intended to represent the ARLG or are supported by the ARLG will be reviewed by the Publication Committee per the Publication Committee charter and must include the following statement: “Research reported in this publication was supported by the National Institute Of Allergy And Infectious Diseases of the National Institutes of Health under Award Number UM1AI104681. The content is solely the responsibility of the authors and does not necessarily represent the official views of the National Institutes of Health.”

The Publication Committee will ensure that the study results are presented by experts in the field that have working knowledge of the study design, implementation, data synthesis/analysis, and interpretation. The committee goals are to ensure that any confidential or proprietary information is protected, and that all appropriate statistical analyses have been included.

The ARLG Publication Committee will adhere to the trials registration policy adopted by the International Committee of Medical Journal Editors (ICMJE) member journals. This policy requires that all applicable clinical trials be registered in a public trials registry such as ClinicalTrials.gov, which is sponsored by the National Library of Medicine. Other biomedical journals are considering adopting similar policies.

In June 2007, the ICMJE adopted the WHO's definition of clinical trial: "any research study that prospectively assigns human participants or groups of humans to one or more health-related interventions to evaluate the effects on health outcomes."

- Health-related interventions include any intervention used to modify a biomedical or health-related outcome (for example, drugs, surgical procedures, devices, behavioral treatments, dietary interventions, and process-of-care changes).
- Health outcomes include any biomedical or health-related measures obtained in patients or participants, including pharmacokinetic measures and adverse events.

All investigators funded by the NIH must submit or have submitted for them to the National Library of Medicine's PubMed Central an electronic version of their final, peer-reviewed manuscripts upon acceptance for publication, to be made publicly available no later than 12 months after the official date of publication. The NIH Public Access Policy ensures the public has access to the published results of NIH-funded research. It requires investigators to submit final peer-reviewed journal manuscripts that arise from NIH funds to the digital archive PubMed Central upon acceptance for publication. Further, the policy stipulates that these papers must be accessible to the public on PubMed Central no later than 12 months after publication.

Refer to: http://publicaccess.nih.gov/

11.0 References

1. Cepheid. Xpert CT/NG Assay package insert. Vol. 301-0234, Rev B (Cepheid, 2013).
2. Hologic. Aptima Combo 2 Assay package insert. Vol. 201798 Rev D (2012).
3. Abbott. Abbott RealTime CT/NG Package Insert. Vol. Ref 8L07-91 (2010).
4. WHO. Global Incidence and prevalence of selected curable sexually transmitted infections. (WHO Department of Reproductive Health and Research, 2008).
5. CDC. Sexually Transmitted Disease Surveillance. (2013).
6. Liu, B., *et al.* Chlamydia and gonorrhoea infections and the risk of adverse obstetric outcomes: a retrospective cohort study. *Sex Transm Infect* **89**, 672-678 (2013).
7. O'Brien, J.P., Goldenberg, D.L. & Rice, P.A. Disseminated gonococcal infection: a prospective analysis of 49 patients and a review of pathophysiology and immune mechanisms. *Medicine* **62**, 395-406 (1983).
8. Westrom, L., Joesoef, R., Reynolds, G., Hagdu, A. & Thompson, S.E. Pelvic inflammatory disease and fertility. A cohort study of 1,844 women with laparoscopically verified disease and 657 control women with normal laparoscopic results. *Sex Transm Dis* **19**, 185-192 (1992).
9. Rottingen, J.A., Cameron, D.W. & Garnett, G.P. A systematic review of the epidemiologic interactions between classic sexually transmitted diseases and HIV: how much really is known? *Sex Transm Dis* **28**, 579-597 (2001).
10. Deguchi, T., Yasuda, M. & Ito, S. Management of pharyngeal gonorrhea is crucial to prevent the emergence and spread of antibiotic-resistant *Neisseria gonorrhoeae*. *Antimicrob Agents Chemother.* **56**, 4039-4040 (2012).
11. Workowski, K.A. & Bolan, G.A. Sexually transmitted diseases treatment guidelines, 2015. *MMWR Recomm Rep* **64**, 1-137 (2015).
12. Zakher, B., Cantor, A.G., Pappas, M., Daeges, M. & Nelson, H.D. Screening for gonorrhea and chlamydia: a systematic review for the U.S. Preventive Services Task Force. *Ann Intern Med* **161**, 884-893 (2014).
13. Ota, K., Tamari, I. & Smieja, M. Detection of *Neisseria gonorrhoeae* and *Chlamydia trachomatis* in pharyngeal and rectal specimens using the BD ProbeTec ET system, the Gen-Probe Aptima Combo 2 assay and culture. *Sexually Transmitted Infections* **85**, 182-186 (2009).
14. Schachter, J., Moncada, J. & Liska, S. Nucleic acid amplification tests in the diagnosis of chlamydial and gonococcal infections of the oropharyngx and rectum in men who have sex with men. *Sexually Transmitted Diseases* **35**, 637-642 (2008).
15. Trebach, J.D., Chaulk, C.P., Page, K.R., Tuddenham, S. & Ghanem, K.G. *Neisseria gonorrhoeae* and *Chlamydia trachomatis* among women reporting extragenital exposures. *Sex Transm Dis* **42**, 233-239 (2015).
16. Bachmann, L., Johnson, R. & Cheng, H. Nucleic acid amplications test for diagnosis of *Neisseria gonorrhoeae* and *Chlamydia trachomatis* rectal infections. *Journal of Clinical Microbiology* **48**, 1827-1832 (2010).
17. Cosentino, L.A., *et al.* Use of nucleic acid amplification testing for diagnosis of anorectal sexually transmitted infections. *J Clin Microbiol* **50**, 2005-2008 (2012).
18. Geelen, T.H., *et al.* Performance of cobas(R) 4800 and m2000 real-time assays for detection of *Chlamydia trachomatis* and *Neisseria gonorrhoeae* in rectal and self-collected vaginal specimen. *Diagn Microbiol Infect Dis* **77**, 101-105 (2013).
19. Moncada, J., Schachter, J., Liska, S., Shayevich, C. & Klausner, J.D. Evaluation of self-collected glans and rectal swabs from men who have sex with men for detection of

Chlamydia trachomatis and *Neisseria gonorrhoeae* by use of nucleic acid amplification tests. *J Clin Microbiol* **47**, 1657-1662 (2009).

20. Perry, M.D., Jones, R.N. & Corden, S.A. Is confirmatory testing of Roche cobas 4800 CT/NG test *Neisseria gonorrhoeae* positive samples required? Comparison of the Roche cobas 4800 CT/NG test with an opa/pap duplex assay for the detection of *N gonorrhoeae*. *Sex Transm Infect* **90**, 303-308 (2014).
21. Harryman, L.*, et al.* Comparative performance of culture using swabs transported in Amies medium and the Aptima Combo 2 nucleic acid amplification test in detection of Neisseria gonorrhoeae from genital and extra-genital sites: a retrospective study. *Sex Transm Infect* **88**, 27-31 (2012).
22. Walsh, A., Rourke, F.O. & Crowley, B. Molecular detection and confirmation of Neisseria gonorrhoeae in urogenital and extragenital specimens using the Abbott CT/NG RealTime assay and an in-house assay targeting the porA pseudogene. *Eur J Clin Microbiol Infect Dis* **30**, 561-567 (2011).
23. Goldenberg, S., Finn, J., Sedudzi, E., White, J. & Tong, C. Performance of the GenExpert CT/NG Assay compared to that of the Aptima AC2 Assay for Detection of Rectal *Chlamydia trachomatis* and *Neisseria gonorrhoeae* by use of Residual Aptima Samples. *Journal of Clinical Microbiology* **50**, 3867-3869 (2012).
24. Cosentino, L.*, et al.* A Validation Study of the Cepheid Xpert CT/NG for detecting *Chlamydia trachomatis* and *Neisseria gonorrhoeae* in Rectal Samples. *Sex Transm Infect* **91**, A1-A258 (2015).
25. CDC. Recommendations for the Laboratory-Based Detection of Chlamydia trachomatis and Neisseria gonorrhoeae. (2014).
26. Gen-Probe. APTIMA Chlamydia trachomatis Assay. Vol. 501799 Revision D (San Diego, 2012).
27. Gen-Probe. APTIMA Neisseria gonorrhoeae Assay. Vol. 502486 Revision A 73 (San Diego, 2011).
28. Boyadzhyan, B., Yashina, T., Yatabe, J.H., Patnaik, M. & Hill, C.S. Comparison of the APTIMA CT and GC assays with the APTIMA combo 2 assay, the Abbott LCx assay, and direct fluorescent-antibody and culture assays for detection of Chlamydia trachomatis and Neisseria gonorrhoeae. *J Clin Microbiol* **42**, 3089-3093 (2004).
29. Chernesky, M.A.*, et al.* Ability of new APTIMA CT and APTIMA GC assays to detect Chlamydia trachomatis and Neisseria gonorrhoeae in male urine and urethral swabs. *J Clin Microbiol* **43**, 127-131 (2005).
30. Moncada, J., Donegan, E. & Schachter, J. Evaluation of CDC-recommended approaches for confirmatory testing of positive Neisseria gonorrhoeae nucleic acid amplification test results. *J Clin Microbiol* **46**, 1614-1619 (2008).
31. Tabrizi, S.N.*, et al.* Evaluation of six commercial nucleic acid amplification tests for detection of Neisseria gonorrhoeae and other Neisseria species. *J Clin Microbiol* **49**, 3610-3615 (2011).
32. BD. BD ProbeTec *Chlamydia trachomatis* (CT) Qx Amplified DNA Assay package insert. Vol. 8081408 (2010).
33. BD. BD ProbeTec *Neisseria gonorrhoeae* (GC) Qx Amplified DNA Assay package insert. Vol. 8081409 (2010).
34. FDA. Establishing the performance characteristics of *in vivo* diagnostics devices for *Chlamydia trachomatis* and/or *Neisseria gonorrhoeae*: screening and diagnostic testing. (2011).

35. FDA. Statistical Guidance on Reporting Results from Studies Evaluating Diagnostic Tests. (2007).

APPENDIX A - Schedule of Events

	Enrollment
**Clinical Evaluations**	
Review of eligibility	X
Verbal consent	X
Baseline CRFs	X
**Collection of Specimens**	
Rectal swabs	X
Pharyngeal swabs	X

Antibacterial Resistance Leadership Group (ARLG)

Performance of Nucleic Acid Amplification Tests for the Detection of *Neisseria gonorrhoeae* and *Chlamydia trachomatis* in Extragenital Sites

GC Statistical Analysis Plan
Version 1.0

Scott Evans, Lauren Komarow, Thuy Tran
Harvard T. H. Chan School of Public Health

April 10, 2017

CONFIDENTIAL

CONFIDENTIAL

CONFIDENTIAL MATERIAL

The content of this statistical analysis plan is confidential; please do not distribute.

INTRODUCTION

This document describes the content proposed for the statistical monitoring and primary statistical analysis of the study titled "Performance of Nucleic Acid Amplification Tests for the Detection of Neisseria gonorrhoeae (NG) and Chlamydia trachomatis (CT) in Extragenital Sites." The focus of this analysis will include the primary and secondary objectives to evaluate diagnostic accuracy of the following three nucleic acid amplification test (NAAT) platforms for the detection of NG and CT from the extragenital sites of the pharynx and the rectum:

- Xpert® CT/NG Assay [Cepheid]
- Combo 2 ® Assay [Hologic]
- Abott RealTime Chlamydia trachomatis and Neisseria gonorrhoeae assay [Abbott].

A subset of these analyses (as described herein) will form the basis of reports provided to the independent statistician as part of independent interim study monitoring while the study is ongoing. Therefore, this analysis plan includes a description of the key analyses which might lead to the modification of the study sample size, and hence also forms the core of any presentation or publication used to disseminate the primary conclusions of the study.

KEY UPDATES TO STATISTICAL ANALYSIS PLAN, VERSION 1.0 (DATED: APRIL 10, 2017):

N/A

PROTOCOL HISTORY

- Protocol Version 1.0 (April 25, 2016, Protocol ARLG_pNAAT-Yr3)
- Protocol Version 2.0 (August 1, 2016, Protocol ARLG_pNAAT-Yr3)

CONFIDENTIAL

LIST OF ABBREVIATIONS

**ARLG**	Antibacterial Resistance Leadership Group
**ASIS**	Anatomic Site Infection Status
**CDC**	Center for Disease Control and Prevention
**CFR**	Code of Federal Regulations
**CT**	*Chlamydia trachomatis*
**DCRI**	Duke Clinical Research Institute
**DNA**	Deoxyribonucleic acid
**FDA**	Food and Drug Administration
**HIV**	Human immunodeficiency virus
**ICMJE**	International Committee of Medical Journal Editors
**ID**	Identification
**IFU**	Instructions for Use
**IRB**	Institutional Review Board
**ISRC**	Independent Study Review Committee
**LGBT**	Lesbian, gay, bisexual and transgender
**NAAT**	Nucleic acid amplification test
**NR**	This means no test was run and there is no result
**NPA**	Negative percent agreement
**NPV**	Negative predictive value
**NG**	*Neisseria gonorrhoeae*
**NIH**	National Institutes of Health
**OHRP**	Office of Human Research Protections
**PPA**	Positive percent agreement
**PPV**	Positive predictive value
**PI**	Principal Investigator
**RNA**	Ribonucleic acid
**rRNA**	Ribosomal ribonucleic acid
**SDMC**	Statistical and Data Monitoring Center
**STD**	Sexually transmitted diseases
**WHO**	World Health Organization

CONFIDENTIAL

STUDY SCHEMA AND OBJECTIVES

DESIGN	A cross-sectional, single visit study to evaluate the diagnostic accuracy of three nucleic acid amplification tests (NAATs) for detection of *Neisseria gonorrhoeae* and *Chlamydia trachomatis* from a set of four swabs each collected from both the pharyngeal and rectal sites, respectively.
DURATION	This is a single visit study. It is estimated that the study will take between 6 and12 months after enrollment of the first participant to fully enroll.
SAMPLE SIZE	Up to 2,500 participants
POPULATION	Symptomatic or asymptomatic male, female, or transgender participants:<br>o Who are patients attending a participating clinic for evaluation of sexually transmitted disease (STD) , and<br>o ≥18 years of age at date of screening, and<br>o Able and willing to provide informed consent, and<br>o Willing to comply with study procedures, including collection of 4 swabs each from the pharynx and rectum for NG and CT testing.
NUMBER OF SITES	Up to 10
STRATIFICATION	Randomization of the swab order will not be stratified. Swabs will be collected from all participants at each extragenital site.
DIAGNOSTICS	While the diagnostic accuracy of three NAATs will be evaluated, a total of four swab kits will be collected from both the pharyngeal and rectal sites, respectively (8 swabs in total). The swab kits associated with the NAATs under investigation will be tested using the corresponding laboratory test assay and test system as defined in **Table 1**. The remaining NAAT will only be performed in cases of discordant results and serve as the tiebreaker assay. There will be no evaluation for diagnostic accuracy for the tie breaker NAAT.

1.1. PRIMARY OBJECTIVES

1.1.1. For each NAAT under evaluation, estimate the positive percent agreement (PPA) and negative percent agreement (NPA) for detection of the organism and extragenital site combinations listed below.

- *Neisseria gonorrhoeae* in rectal swabs
- *Neisseria gonorrhoeae* in pharyngeal swabs
- *Chlamydia trachomatis* in rectal swabs
- *Chlamydia trachomatis* in pharyngeal swabs

1.2. SECONDARY OBJECTIVES

1.2.1. Global analyses

For each NAAT under evaluation, positive predictive values (PPVs), negative predictive values (NPVs), positive likelihood ratios, and negative likelihood ratios will be calculated for detection of the organism and extragenital site combinations listed below.

- *Neisseria gonorrhoeae* in rectal swabs
- *Neisseria gonorrhoeae* in pharyngeal swabs
- *Chlamydia trachomatis* in rectal swabs
- *Chlamydia trachomatis* in pharyngeal swabs

1.2.2. Subgroup analyses

For each NAAT under evaluation, to estimate the PPAs, NPAs, PPVs, and NPVs for detection of NG and CT from rectal and pharyngeal swab specimens by sex and by anatomic site-specific symptom status.

CONFIDENTIAL

1.3. EXPLORATORY OBJECTIVES

1.3.1. Application of developed diagnostic benefit:risk analyses, including BED-FRAME methodologies[1].

Table 1: List of Corresponding Swab Collection Kits, Laboratory Assay and Laboratory Machine

Swab Collection Kit Name	Corresponding Laboratory Test Assay	Corresponding Laboratory Test System (Machine)
NAATs Under Investigation (as defined in Section 1.2 of Protocol)		
**Cepheid** Xpert® CT/NG Vaginal/ Endocervical Specimen Collection Kit	Xpert® CT/NG assay<br>A combination test using Real-time PCR technique to detect two noncontiguous chromosomal DNA regions from NG (NG2 and NG4) - both of which must be positive to yield a positive result - and one chromosomal DNA target from CT (CT1).	GeneXpert System
**Hologic** Aptima® Multitest Swab Specimen Collection Kit	Aptima® Combo 2 for CT/NG assay<br>A combination test using target capture, transcription mediated amplification, and dual kinetic assay to detect regions of the rRNA from the 16S rRNA of GC and the 23S rRNA from CT using labeled DNA probes.	Panther system
**Abbot** multi-Collect Specimen Collection Kit	Abbott RealTime CT/NG assay<br>A combination test that uses a real-time PCR assay to detect a highly-conserved region within the Opa gene of NG and two distinct regions within the CT cryptic plasmid DNA .	Abbott m2000 RealTime System
Tiebreaker Assay (as defined in Section 1.2 of Protocol)		
**Hologic** Aptima® Multitest Swab Specimen Collection Kit	Aptima® Chlamydia trachomatis assay and/or the Aptima® Neisseria gonorrhoeae assay<br>These tests use target capture, transcription mediated amplification, and hybridization protection assays to identify the presence of RNA from the organism of interest in the clinical sample. (Note: The targets from the 16S rRNA for both GC and CT are different than those used in the Combo 2® Assay.)	Tigris DTS system

[1] Evans, S.R., Pennello, G., Pantoja-Galicia, N., Jiang, H., Hujer, A.M., Hujer, K.M., Manca, C., Hill, C., Jacobs, M.R., Chen, L. and Patel, R., 2016. Benefit-risk evaluation for diagnostics: a framework (BED-FRAME). Clinical Infectious Diseases, p.ciw329.

ANALYSIS PLAN OVERVIEW

A. General Analysis Considerations ... 8
B. Statistical Monitoring Considerations ... 8
C. Interim Analysis Considerations ... 8
D. Final Analysis Considerations ... 9
E. Report Distribution List ... 9
F. Application Validation ... 10
G. Analysis Plan ... 10
1. Study Population ... 10
1.1. Accrual and Eligibility Violations ... 10
1.2. Study Population Characteristics ... 10
2. Study Status ... 11
3. Swab Collection Completeness and Monitoring ... 11
3.1. Swab Collection Completeness and Complications ... 11
3.2. Laboratory Device Monitoring ... 12
4. Swab Testing Completeness and Monitoring ... 12
5. Infection Status by Anatomic Site and Organism ... 12
5.1. NAAT Test Results ... 12
5.2. Anatomic Site Infection Status (ASIS) Determination ... 13
5.3. Interim Analysis ... 14
5.4. Final Analysis ... 18
5.5. Exploratory Analyses ... 21
H. APPENDIX 1: CBAR SOP PROG.10033 ... 22

CONFIDENTIAL

A. General Analysis Considerations

Data summaries and analyses will be presented overall and by sex or, where appropriate, by anatomic site and organism.

Because the Master-GC study is a single visit study (i.e, no follow-up), the only date captured in this study is the date of a participant's clinic visit (recorded on the DEMOG case report form) and represents both the "study entry" and "off study" dates.

To ensure participant confidentiality, any listing of individual patient level data will be minimized as much as possible during study monitoring and for interim and final analyses. If such data are provided, they will be indexed with a unique blinded identifier or with identifiers removed. Study dates will not be presented.

Of note, while participants may need to seek additional care beyond this single visit, this is beyond the purview of the study. Similarly, this study will not be used to inform or determine treatment practices.

B. Statistical Monitoring Considerations

Routine statistical monitoring of accrual, study conduct, and completeness of swab and data collection will be conducted by the Harvard statistical team. A monitoring report summarizing these components will be distributed by the Harvard team on a bi-monthly basis until completion of study accrual; see sections listed below for details. The report will be distributed to the core protocol team (see Section E for details). It is planned at this time that distribution of the first monitoring report will occur two weeks after transfer of data from DCRI to Harvard has been tested and successfully confirmed by both groups.

1. Study Population (Section G, Part 1, pg 10)
2. Study Status (Section G, Part 2, pg 11)
3. Swab Collection Completeness and Monitoring (Section G, Part 3, pg 11)
4. Swab Testing Completeness (Section G, Part 4, pg 12)

Additional monitoring and querying of all data will be conducted concurrently with the statistical monitoring report to ensure cross-form consistency and data quality.

C. Interim Analysis Considerations

The Master-GC study will undergo interim review every 500 participants or every 3 months after the enrollment of the first participant (whichever occurs first) by an independent statistician who will not have an association with the protocol or device companies.

The study cannot be stopped at the interim analysis for reaching regulatory goals in order to preserve error rates / coverage probability and to ensure enough data for subgroup analyses. Since the trial cannot be stopped for attainment of the regulatory goal, no adjustment to confidence levels is necessary.

Infection rates will be evaluated by the independent statistician to determine whether sample size adjustments are warranted to ensure sufficient number of infected participants to estimate PPAs with desired precision. The sample size will not be adjusted based on the observed PPAs and NPAs (which will not be reviewed while the study is ongoing). If infection is more prevalent than expected, a smaller sample size may be accepted. If infection is rarer than anticipated, then increases to sample size will be considered. Detailed analysis considerations are provided in Section 5.3.

The independent statistician will also monitor study accrual and review endpoint evaluability with particular focus on the frequency of tests with equivocal results, invalid results, or no results for each platform. Sample size adjustments may also be considered if there is evidence that results categorized as equivocal, invalid, or no results will impact study accrual time and/or data analyses.

Two separate analysis reports will be prepared and distributed for each interim review. The summary below provides the sections of this analysis plan that are relevant to each report; see sections for details.

<u>Open Administrative Report:</u> This report will be distributed to the independent statistician, core study team and DMID representative (see Section E for details). Presentation of data by swab or platform will be minimized as much as possible; any inclusion will use generic identifiers (i.e., platform or swab 1, 2, etc.) as appropriate. The report will include summaries of:

CONFIDENTIAL

1. Study Population (Section G, Part 1, pg 10)
2. Study Status (Section G, Part 2, pg 11)
3. Swab Collection Completeness and Monitoring (Section G, Part 3, pg 11)
4. Swab Testing Completeness (Section G, Part 4, pg 12)

Closed Administrative Report: This report will be distributed only to the independent statistician (see Section E). The report will include analysis results from all assay platforms (swab and platform names will be presented). Upon completing review of interim report

1. Study Population (Section G, Part 1, pg 10)
2. Study Status (Section G, Part 2, pg 11)
3. Swab Collection Completeness and Monitoring (Section G, Part 3, pg 11)
4. Swab Testing Completeness (Section G, Part 4, pg 12)
5. Infection Status by Anatomic Site and Organism (Section G, Part 5, pg 12)

Of note, some analyses, tables or figures may be omitted at interim analyses if there are insufficient data to warrant analysis. Additional analyses may be provided if requested by the independent statistician.

Upon completing review of the interim analysis reports, it is anticipated that the independent statistician will provide recommendations to the protocol chair, protocol clinician and project lead. The choice of sharing these recommendations with members of protocol team will be left to the discretion of this latter group.

D. Final Analysis Considerations

The primary analysis will be conducted once data from the last participant enrolled has been received. The final analysis report will be distributed to the protocol team after Harvard receives the final, locked data transfer from DCRI and validation of primary analyses and internal review at Harvard are completed. The protocol team is defined in Section E.

The final analysis population will include participants who meet all eligibility criteria and provide four swabs from at least one anatomic site.

The primary analysis will evaluate the result for each diagnostic test (i.e., test under consideration) and compare with the ASIS for each anatomic site and organism combination. For each diagnostic test, positive percent agreement (PPA) and negative percent agreement (NPA) will be estimated using 95% confidence intervals. Confidence intervals will be estimated using the Score method. The primary analysis will also follow FDA guidance for incorporating indeterminate ASIS or test results for the test under consideration that are equivocal. As a result, all combinations of Infected/Indeterminate/Not Infected with all outcomes from the test under consideration (Positive/Equivocal/Negative/No result or Invalid) will be presented. If the test under consideration has "no result" because the test was not run and no attempt was made to test the sample, it will be excluded from the primary analysis.

Sensitivity analyses associated with the primary analyses will be conducted to examine the impact of different classifications of indeterminate results (i.e., all infected, all not infected, account for symptom status). Additional subgroup and secondary analyses are planned.

E. Report Distribution List

Unless otherwise mentioned, distribution lists for monitoring and analysis reports are comprised of:

Protocol Team: arlg.gc@mc.duke.edu members

Core Team: Protocol Chair, Protocol Clinician, Project Lead, Statisticians, Data Management, Clinical Trials Manager, Clinical Research Associate, Regulatory Associate.

DMID: DMID representative

DIR: Designated Independent Reviewer

CONFIDENTIAL

**Table 2: Report Distribution Summary**		
**Report**	**Frequency**	**Distribution List**
Harvard Monitoring Report	Bi-monthly (or as determined by core team)	Core Team
Open Administrative Interim Report	Every 3 months or every 500 participants (whichever comes first)	DIR, Core Team, DMID
Closed Administrative Interim Report	Every 3 months or every 500 participants (whichever comes first)	DIR, Statisticians
Final Analysis Report	End of study per study timeline	Protocol Team

F. Application Validation

All study-specific programs for creation of derived datasets for derivation of the primary outcomes defined in this document will require application validation per standing operating procedures (SOP) defined in CBAR PROG.10033 as appropriate; when applicable, requirements for independent results verifications of these datasets and application validation requirements for analysis programs are provided as annotations throughout the analysis plan. A copy of CBAR PROG.10033 is provided in Section H.

G. Analysis Plan

Throughout, annotations in square brackets ([xxx]) provide the data source.

1. Study Population

1.1. Accrual and Eligibility Violations

1. Table: Number (%) enrolled overall and by month and site.

   Note: Dates of first and last enrollments will be provided in a footnote to the table.

2. Table: Number (%) enrolled by month, site and reported sex at birth [DEMOG].
3. List: Description of violations of eligibility criteria if applicable. [INCEXC]

   Note: Participants enrolled and later found ineligible will be excluded from all analyses and be included in this listing. Information on whether swabs were collected for these participants will be noted.

4. Figure: Observed, cumulative and targeted accrual by month.

   Note: Targeted accrual is assumed to be approximately 209 participants per month for 12 months or 417 participants per month for 6 months to achieve full accrual of 2500 participants.

1.2. Study Population Characteristics

Table summaries will present the following study population characteristics overall and by sex. All variables, as noted below, will be analyzed on the continuous scale or as categories or both as appropriate.

For continuous variables, summary statistics will include # of participants, # of missing data points, mean and standard deviation, median (Q1-Q3), P10 and P90, and minimum and maximum.

For categorical variables, summary statistics will include number (%) for each category. In calculation of percentages, participants with missing data will not be included in the denominator.

1. Demographics
   a. Sex at birth: By category (male/female) [DEMOG]
   b. Gender: By category (man, woman, transman, transwoman, genderqueer, additional category, decline to answer) [DEMOG]
   c. Self-reported race, ethnicity and race/ethnicity as defined by NIH reporting standards: By category [DEMOG]

CONFIDENTIAL

d. Current age on day of study entry (years): Continuous and by age group (18-29, 30-39, 40-49, 50-59, 60+) [DEMOG]

2. Health Status [*For interim and final analyses only*]

a. Abnormalities or symptoms in the pharynx in the past 7 days: By category (Yes/No) [SIGNS AND SYMPTOMS]
Note: If yes, sub-categorization of the reported symptom will also be provided. This includes sore throat, painful swallowing, swollen/tender lymph nodes in the neck, and other symptom (with listed reasons).

b. Abnormalities or symptoms in the rectum in the past 7 days: By category (Yes/No) [SIGNS AND SYMPTOMS]
Note: If yes, sub-categorization of the reported symptom will also be provided. This includes rectal discharge, rectal bleeding, rectal itching, painful bowel movements, and other symptom (with listed reasons).

2. Study Status

As noted, the date of "study entry" is the same as the "off study" date. Study status will be determined by cross checking the study completion form with the sample collection form.

1. Table: Number (%) by category of study status.

Categories: Completed study per sample collection form; incomplete- subject withdrew consent; incomplete – investigator decision; other reasons (with listing of reasons). [STUDY COMPLETION AND SAMPLE COLLECTION]

Note: Study completion is defined as collection of at least four swabs from one anatomic site.

3. Swab Collection Completeness and Monitoring

All tables will be presented by anatomic site.

Of note, swab collection data will also be examined by study site and presented if low data completeness or high numbers of complications are observed.

3.1. Swab Collection Completeness and Complications

1. Table: Number (%) of participants reporting collected swabs.

Categories: All 4 swabs, 3 swabs, 2 swabs, 1 swab, no swabs. [SAMPLE COLLECTION]

Note: [CLOSED administrative report at interim analysis and final analyses only]: List swabs by name for each numeric category.

2. Listing/Table: Number (%) of participants with swabs not collected per assigned swab order. [SAMPLE COLLECTION]

Note: If few deviations are reported, list the reported order the swabs were collected.

3. Table: Number (%) of participants reporting sample collection complications.

Categories: Patient declined – due to excessive discomfort; patient declined – other reason; problem with testing materials, other (with listed reasons). [SAMPLE COMPLICATIONS]

Note: If few complications are reported, include the number of swabs collected (4, 3, 2, 1 or no swabs) for each complication. For CLOSED administrative report at interim analysis and final analyses only, list by swab name.

CONFIDENTIAL

3.2. Laboratory Device Monitoring

The testing laboratories will maintain a log of all unanticipated device-related complications leading to no test, such as absence of transport media, quantity not sufficient, interference issues during testing, specimen transport collection system damage or incorrect transport system. No additional monitoring will be conducted.

4. Swab Testing Completeness and Monitoring

1. Table: Number (%) of participants with swabs tested (presented by anatomic site).

   Categories: All 4 swabs tested; 1-3 swabs tested; no swabs tested. [LAB DATA]

   For participants with fewer than 4 swabs tested, reasons why the laboratory did not test the swabs will be listed. [LAB DATA]

5. Infection Status by Anatomic Site and Organism

[NOTE: Interim (Closed Report) and Final Analyses Only]

5.1. NAAT Test Results

Possible test results for each NAAT platform (as listed in **Table 3**) will be used for each anatomic site (pharynx or rectum) and organism (NG or CT) combination. These four combinations include NG of pharynx, CT of pharynx, NG of rectum, and CT of rectum.

Table 3: Summary of NAAT Test Results

NAAT	Possible Test Results	Notes
Xpert® CT/NG Assay (Cepheid)[2]	1. Not detected<br>2. Detected<br>3. Invalid (sample processing control or sample adequacy control failed)<br>4. Error (probe check control failed)<br>5. No result (insufficient data was collected, e.g. test aborted).	Initial invalid, error, or no result tests will be repeated. If the repeat test returns invalid, error, or no result, the final result will be considered an invalid and will be categorized as no result (NR) for the ASIS determination below. If the repeat test returns not detected (negative) or detected (positive), this will be the result used for the ASIS determination.
Combo 2 ® Assay (Hologic)[3]	1. Negative<br>2. Positive<br>3. Equivocal (result between positive and negative)<br>4. Invalid (parameter outside the normal expected ranges).	Initial equivocal and invalid test results will be repeated. If the repeat test result is equivocal or invalid, the final test result will be considered an equivocal test result for the ASIS determination below. If the repeat test returns negative or positive, this will be the result considered for the ASIS determination.
Abbott RealTime CT/NG assay (Abbott)[4]	For NG:<br>1. Positive (detected, with cycle number less than or equal to the assay cut-off)<br>2. Negative (no evidence of amplification or cycle number greater than the assay cut-off).<br>3. Note: An equivocal interpretation does not apply.<br><br>For CT:<br>1. Positive (detected, with cycle number less than or equal to the assay cut-off)<br>2. Negative (no evidence of amplification)<br>3. Equivocal (cycle number beyond the assay cut-off).	For CT only:<br>A sample with initial interpretation of "equivocal" for CT will be retested. If the repeat test returns negative or positive, this will be the result considered for the ASIS determination. If the repeat test result is equivocal, the final test result will be considered an equivocal test result for the ASIS determination below.

CONFIDENTIAL

[2] Cepheid. Xpert CT/NG Assay package insert. Vol. 301-0234, Rev B (Cepheid, 2013).
[3] Hologic. Aptima Combo 2 Assay package insert. Vol. 201798 Rev D (2012).
[4] Abbott. Abbott RealTime CT/NG Package Insert. Vol. Ref 8L07-91 (2010).

Tiebreaker assays: Aptima® Chlamydia trachomatis assay (Gen-Probe) and the Aptima® Neisseria gonorrhoeae assay (Gen-Probe)	1. Negative<br>2. Positive<br>3. Equivocal (result between negative and positive ranges)<br>4. Invalid (parameter outside the normal expected ranges).	Initial equivocal and invalid test results will be repeated. If the repeated test remains equivocal or invalid, it will be considered an equivocal test result for the ASIS determination below. If the repeat test returns negative or positive, this will be the result considered for the ASIS determination.

5.2. Anatomic Site Infection Status (ASIS) Determination

Per protocol, determination of the ASIS will be NAAT-specific and evaluated for each anatomic site and organism combination.

Possible ASIS outcomes include:

- Infected
- Not infected
- Indeterminate
- Invalid, exclude from analysis

The anatomic site is considered to be **infected** when both reference test results are positive.

The anatomic site is considered to be **not infected** when both reference test results are negative.

If there is discordance between the reference tests, an additional NAAT test will be performed as a tiebreaker. In this case, agreement of 2/3 of the reference NAATs will determine the ASIS. If two tests are equivocal or one equivocal and one not run, the third test result will stand as the ASIS if positive or negative. If two tests are not run, the ASIS will be considered invalid and will be excluded from the analysis.

All possible test result combinations are shown in **Table 4.** The tiebreaker test will be run by the lab if any NAAT is not concordant with the others and interpreted only in the case of discordant results between the two planned reference tests for each assay. As the tiebreaker test is not a combination test, the tiebreaker will only be run for the organism with disagreement (e.g. if NG disagrees and CT agrees, the tiebreaker will only be run for NG).

To determine the ASIS, the test result for each respective site (pharynx or rectum) and each organism (NG or CT) for each NAAT platform will be used.

Table 4: Determination of the Anatomic Site Infection Status (ASIS)
Note:
*E **=** equivocal result;
****NR = no result. This can occur either because the test result was invalid or because the test could not be run (e.g. too little sample, improperly shipped, no sample received).

Comparator NAAT 1 Result	Comparator NAAT 2 Result	Tiebreaker NAAT Result	Anatomic Site Infection Status (ASIS)
+	+	Not indicated	Infected
+	-	+	Infected
+	E*	+	Infected
+	NR**	+	Infected
+	-	-	Not infected
+	-	E	Indeterminate
+	-	NR	Indeterminate
+	E	-	Indeterminate
+	E	E	Infected
+	E	NR	Infected
+	NR	-	Indeterminate
+	NR	E	Infected
+	NR	NR	Invalid, remove from analysis
-	-	Not indicated	Not infected
-	+	-	Not infected

CONFIDENTIAL

-	E	-	Not infected
-	NR	-	Not infected
-	+	+	Infected
-	+	E	Indeterminate
-	+	NR	Indeterminate
-	E	+	Indeterminate
-	E	E	Not infected
-	E	NR	Not infected
-	NR	+	Indeterminate
-	NR	E	Not infected
-	NR	NR	Invalid, remove from analysis
E	+	+	Infected
E	-	-	Not infected
E	+	-	Indeterminate
E	+	E	Infected
E	+	NR	Infected
E	-	+	Indeterminate
E	-	E	Not infected
E	-	NR	Not infected
E	NR	+	Infected
E	NR	-	Not infected
E	NR	E	Indeterminate
E	NR	NR	Invalid, remove from analysis
NR	+	+	Infected
NR	-	-	Not infected
NR	NR	Not indicated	Invalid, remove from analysis
NR	+	-	Indeterminate
NR	+	E	Infected
NR	+	NR	Indeterminate
NR	-	+	Indeterminate
NR	-	E	Not infected
NR	-	NR	Invalid, remove from analysis
NR	E	+	Infected
NR	E	-	Not infected
NR	E	E	Indeterminate
NR	E	NR	Invalid, remove from analysis

5.3. Interim Analysis

5.3.1. Interim Analysis Considerations

The study cannot be stopped at the interim for reaching regulatory goals in order to preserve error rates / coverage probability and to ensure enough data for subgroup analyses. Since the trial cannot be stopped for attainment of the regulatory goal, no adjustment to confidence levels are necessary.

Although it is anticipated that swab collection and data completeness will be high for both anatomical sites, both the Intention-to-Diagnose (ITD) and modified Intention-to-Diagnose (mITD) infection rates[5], as described in **Table 5**, will be estimated. It is expected that disease prevalence (infection rate) of NG in the rectum, NG in the pharynx, and CT in the rectum will each be greater than 7.5% in the population under evaluation. Disease prevalence of CT in the pharynx is expected to be rare.

CONFIDENTIAL

[5] Fundamental Concepts for New Clinical Trialists. A Evans, S. and A Ting, N. 9781420090871. https://books.google.com/books?id=G1IUPQAACAAJ. 2015. Taylor & Francis.

Table 5: Infection rate calculation for each test under consideration

**Intent-to-Diagnose (ITD) Infection Rate**	Number of infected ASIS results for test under consideration / Total number of ASIS results[1]
**Modified Intent-to-Diagnose (mITD) Infection Rate**	Number of infected ASIS results for test under consideration / Total number of ASIS results with exclusion of invalid results[2]

[1] *The denominator will include the sum of all infected, not infected, indeterminate and invalid ASIS results.*
[2] *The denominator will include the sum of all infected, not infected, and indeterminate ASIS results.*

Operationally, a range of scenarios for the unobserved data will be generated at each interim review to assist with the decision making regarding sample size adjustments. To ensure enough infected participants to estimate PPAs with desired precision, the lowest ITD/mITD infection rate across the three assays and the three organism/site combinations (except CT in the pharynx) will be used as the observed rate for interim analysis. Of note, it is assumed that there will be greater precision to evaluate NPA as it is expected that there will be more not-infected results than infected results for each anatomic site.

Table 6 and **Table 7** illustrate hypothetical scenarios when the observed infection rate after the first 1000 participants is rarer than anticipated (equal to 5%, **Table 6)** or more prevalent than anticipated (equal to 10%, **Table 7**), respectively. For both tables, a range of infection rates for the unobserved data (scenarios A-C) are also presented to demonstrate the probability and corresponding total sample sizes to obtain 150, 175 or 200 disease positive participants, respectively, at the end of study.

If infection is rarer than anticipated, then increases to sample size may be considered as demonstrated in scenario A from Table 6. In this case, should the observed prevalence of 5% remain unchanged for the unobserved data, then enrollment of approximately 3200 total participants would be needed to ensure at least 80% probability of obtaining 150 disease positive participants. However, should the infection rate increase to 9% (Scenario C, Table 6), then sample size adjustments may not be warranted.

Alternatively, if infection is more prevalent than expected, a smaller sample size may be considered as demonstrated in scenarios A and B from **Table 7**.

Sample size adjustments may also be considered if there is evidence that the number of ASIS results categorized as invalid will impact study accrual time and/or data analyses.

Table 6: Hypothetical illustration of simulated infection rates after first 1000 participants and observed prevalence of 5%

**Observed Response Rate and Count**		
**ITD infection rate at interim**	**Prevalence of Disease Positive (%)**	**Disease Positive (N)**
	**5%**	**50 participants**

**Example Scenarios for Unobserved Data**								
**Scenario**	**Assumed prevalence rate[1]**	**Average prevalence rate[2]**	**Estimated probability (P) and sample size (N) to obtain:**					
			**150 Disease+ total participants**		**175 Disease+ total participants**		**200 Disease+ total participants**	
			**P**	**N**	**P**	**N**	**P**	**N**
**A**	**5%**	**5%**	<50%	2500	<50%	2500	<50%	2500
			80%	3220	80%	3740	80%	4260
			90%	3340	90%	3860	90%	4380
**B**	**7%**	**6.2%-6.4%**	64%	2500	<50%	2500	<50%	2500
			80%	2589	80%	2956	80%	3323
			90%	2669	90%	3046	90%	3413
**C**	**9%**	**7.2%-7.6%**	80%	2232	76%	2500	<50%	2500
			90%	2302	80%	2519	80%	2807
			99%	2500	90%	2589	90%	2877

[1] The assumed prevalence rate for the remaining unobserved data. [2] Average prevalence rate is the average of the observed and assumed prevalence rates for the entire duration of study for a given sample size.

CONFIDENTIAL

Table 7: Hypothetical illustration of simulated infection rates after first 1000 participants and observed prevalence of 10%

**Observed Response Rate and Count**			
**ITD infection rate at interim**	**Prevalence of Disease Positive (%)**	**Disease Positive (N)**	
	**10%**	**100 participants**	

**Example Scenarios for Unobserved Data**								
**Scenario**	**Assumed prevalence rate[1]**	**Average prevalence rate[2]**	**Estimated probability (P) and sample size (N) to obtain:**					
			**150 Disease+ total participants**		**175 Disease+ total participants**		**200 Disease+ total participants**	
			**P**	**N**	**P**	**N**	**P**	**N**
**A**	**10%**	**10%**	80%	1610	80%	1870	80%	2130
			90%	1670	90%	1930	90%	2190
			>95%	2500	>95%	2500	>95%	2500
**B**	**7%**	**8.1%-8.6%**	80%	1875	80%	2242	63%	2500
			90%	1955	90%	2322	80%	2609
			>95%	2500	>95%	2500	90%	2699
**C**	**5%**	**6.5%-7.3%**	80%	2220	<50%	2500	<50%	2500
			90%	2330	80%	2740	80%	3250
			>95%	2500	90%	2860	90%	3380

[1] The assumed prevalence rate for the remaining unobserved data. [2] Average prevalence rate is the average of the observed and assumed prevalence rates for the entire duration of study for a given sample size.

5.3.2. Interim Analyses

Note: Analysis programs will require independent results verification per CBAR PROG.10033.

a. Table: For each diagnostic assay, frequency of observed test outcomes by anatomical site and organism combination as shown in **Table 8**. [Data source: LAB DATA]

Note: Test results for each diagnostic assay were defined previously in Section 5.1.

Table 8: Frequency of observed test outcomes by anatomical site and organism combination

**NAAT: GeneXpert System Test Results**					
	Not detected	Detected	Invalid	Error	No result
***NG*, rectum**					
***NG*, throat**					
***CT*, rectum**					
***CT*, throat**					
**NAAT: Panther System Test Results**					
	Negative	Positive	Equivocal	Invalid	
***NG*, rectum**					
***NG*, throat**					
***CT*, rectum**					
***CT*, throat**					
**NAAT: Abbott m2000 Test Results**					
	Negative	Positive			
***NG*, rectum**					
***NG*, throat**					
	Negative	Positive	Equivocal		
***CT*, rectum**					
***CT*, throat**					
**NAAT: Tigris DTS system Test Results (TIEBREAKER)**					
	Negative	Positive	Equivocal	Invalid	
***NG*, rectum**					

***NG*, throat**					
***CT*, rectum**					
***CT*, throat**					

b. Table/Figure: Frequency of comparator NAAT result and tiebreaker result combinations to define ASIS as shown in **Table 9**. This will be conducted for each test under consideration and be presented by anatomical site and organism combination. There will be a total of three tables to reflect the three tests under consideration. [Data source: LAB DATA]

Note: Only combinations with a frequency of one or greater will be shown. Any combination not shown will indicate that this combination was not observed (i.e., frequency equal to 0).

Table 9: Frequency of comparator NAAT result and tiebreaker result combinations by anatomical site and organism for test under consideration XX

**Test Under Consideration: XXX**				
**Anatomical Site and Organism: *NG*, rectum**				
Comparator NAAT Result 1	Comparator NAAT Result 1	Tiebreaker NAAT result	ASIS	Frequency (n)
+	+	Not indicated	Infected	XX
+	-	+	Infected	XX
(Remaining observed combinations as described in **Table 4**.)				
**Anatomical Site and Organism: *NG*, throat**				
Comparator NAAT Result 1	Comparator NAAT Result 1	Tiebreaker NAAT result	ASIS	Frequency (n)
+	+	Not indicated	Infected	XX
+	-	+	Infected	XX
(Remaining observed combinations as described in **Table 4**.)				
**Anatomical Site and Organism: *CT*, rectum**				
Comparator NAAT Result 1	Comparator NAAT Result 1	Tiebreaker NAAT result	ASIS	Frequency (n)
+	+	Not indicated	Infected	XX
+	-	+	Infected	XX
(Remaining observed combinations as described in **Table 4**.)				
**Anatomical Site and Organism: *CT*, throat**				
Comparator NAAT Result 1	Comparator NAAT Result 1	Tiebreaker NAAT result	ASIS	Frequency (n)
+	+	Not indicated	Infected	XX
+	-	+	Infected	XX
(Remaining observed combinations as described in **Table 4**.)				

c. Table/Figure: Number (%) of observed total test results, ASIS results, and prevalence (i.e., infection) rate. This will be conducted for each test under consideration and be presented by anatomical site and organism combination as shown in **Table 10** below. [Data source: LAB DATA]

Table 10: Number (%) of observed total test results, ASIS results, and prevalence (i.e., infection) rate for each test under consideration by anatomical site and organism

**Test under consideration:** GeneXpert System								
**Comparator NAATs:** Abbott m2000 and Panther System<br>**Tiebreaker NAAT:** Tigris DTS System								
	Sample Size (N)		ASIS				Prevalence (P, %)	
	N (ITD)	N (mITD)	Infected	Not infected	Indeterminate	Invalid	P (ITD)	P (mITD)
***NG*, rectum**								
***NG*, throat**								
***CT*, rectum**								
***CT*, throat**								

CONFIDENTIAL

**Test under consideration:** Panther System								
**Comparator NAATs:** Abbott m2000 and GeneXpert System<br>**Tiebreaker NAAT:** Tigris DTS System								
	Sample Size (N)		ASIS				Prevalence (P, %)	
	N (ITD)	N (mITD)	Infected	Not infected	Indeterminate	Invalid	P (ITD)	P (mITD)
***NG,* rectum**								
***NG,* throat**								
***CT,* rectum**								
***CT,* throat**								
**Test under consideration:** Abbott m2000								
**Comparator NAATs:** Panther System and GeneXpert System<br>**Tiebreaker NAAT:** Tigris DTS System								
	Sample Size (N)		ASIS				Prevalence (P, %)	
	N (ITD)	N (mITD)	Infected	Not infected	Indeterminate	Invalid	P (ITD)	P (mITD)
***NG,* rectum**								
***NG,* throat**								
***CT,* rectum**								
***CT,* throat**								

a. Table/Figure: Predicted infection rate summary for range of scenarios (see **Table 6** and **Table 7** described in Section 5.3.1).
b. Table: Observed frequency of tiebreaker run versus expected number of runs (number, %).
c. Table: Using Fischer's exact test, the association between of test results and randomized swab order will be examined. Associations will be conducted by anatomical site and organism combination.

5.4. Final Analysis

5.4.1. Endpoint Definitions

For each participant, primary and secondary endpoints will be defined for each anatomical site and organism. [Primary source data: SAMPLE COLLECTION, STUDY COMPLETION and LAB DATA]

a. Primary Endpoints

Note: Derived datasets relating to the derivation of these endpoints will undergo independent results verification in accordance with CBAR PROG.10033.

Anatomic site infection status is determined by the reference standard (described in Section 4.2).

- Infection status for Neisseria gonorrhoeae in the rectum as determined by each NAAT
- Infection status for Neisseria gonorrhoeae in the pharynx as determined by each NAAT
- Infection status for Chlamydia trachomatis in the rectum as determined by each NAAT
- Infection status for Chlamydia trachomatis in the pharynx as determined by each NAAT

5.4.2. Final Analysis Considerations

Note: All dataset derivation programs for this endpoint will undergo independent results verification in accordance with CBAR PROG.10033; validation requirements for analysis programs are stated below.

The result for each diagnostic test will be compared with the ASIS for that anatomic site and organism. PPA and NPA will also be estimated for each diagnostic test with 95% confidence intervals. Confidence intervals will be estimated using the Score method.[6] If the test under consideration has "no result" because the test was not run and no attempt was made to test the sample, the test result will be excluded from primary analysis. If the ASIS result is invalid, this result will be excluded from the primary analysis.

[6] FDA. Establishing the performance characteristics of in vivo diagnostics devices for Chlamydia trachomatis and/or Neisseria gonorrhoeae: screening and diagnostic testing. (2011).

CONFIDENTIAL

FDA guidance documents will be followed as part of the primary analysis to incorporate indeterminate ASIS or test results for the test under consideration that are equivocal.[7] It is recognized that there are pros and cons to the manner in which indeterminates are handled and how these impact the resulting estimates of PPA and NPA. The primary analysis approach is the most conservative and is biased downwards. If PPA is >90% under this scenario, then the conclusion of PPA >90% is clear.

Sensitivity analyses will be conducted as appropriate to evaluate the impact of diagnostic accuracy for a range of scenarios addressing indeterminate ASIS results. This will include counting indeterminate ASIS results against the results for the test under consideration with all combinations of Infected/Indeterminate/Not Infected with all outcomes from the test under consideration (Positive/Equivocal/Negative/No result or Invalid) evaluated. These analyses may not be conservative for calculations of PPA and NPA.

5.4.3. Primary Analyses

The following will be conducted for the three diagnostic tests.

Note: Analysis programs will require independent results verification per CBAR PROG.10033.

a. Table: For each diagnostic assay, frequency of observed test outcomes by anatomical site and organism combination. [See **Table 8** described in Section 5.3.2; data source: LAB DATA]
b. Table/Figure: Frequency of comparator NAAT result and tiebreaker result combinations to define ASIS. This will be conducted for each test under consideration and be presented by anatomical site and organism combination. There will be a total of three tables to reflect the three tests under consideration. [See **Table 9** previously described in in Section 5.3.2; data source: LAB DATA]
c. Table/Figure: Number (%) of observed total test results, ASIS results, and prevalence (i.e., infection) rate. This will be conducted for each test under consideration and be presented by anatomical site and organism combination. [See **Table 10** previously described in Section 5.3.2; data source: LAB DATA]
d. Tables:
    - Cross comparison of number (%) of results of test under consideration versus ASIS;
    - Estimates of PPA and NPA with 95% Score confidence intervals;
    - Due to differences in PPA and NPA calculations for NAAT platforms that do not have an “equivocal” test result when it is the test under consideration, analysis results will be estimated and presented separately based on this distinction as follows:
        - **Table 11** and **Table 12**: Aptima Combo 2® Assay (Hologic) and Abbott RealTime CT Assay (Abbott).
        - **Table 13** and **Table 14**: Xpert® CT/NG Assay (Cepheid) and Abbott RealTime NG Assay (Abbott).
e. Figure: Plot of PPA and 95% confidence interval band versus proportion of indeterminate ASIS results assumed to be positive (range of 0 to 1); vice-versa for NPA plot.
f. Table: Using Fischer’s exact test, the association between of test results and randomized swab order will be examined. Associations will be conducted by anatomical site and organism combination.

[7] FDA. Establishing the performance characteristics of in vivo diagnostics devices for Chlamydia trachomatis and/or Neisseria gonorrhoeae: screening and diagnostic testing. (2011).

CONFIDENTIAL

Table 11: Result of Test under Consideration versus ASIS: Aptima Combo 2® Assay (Hologic) and Abbott RealTime CT Assay (Abbott)

		ASIS		
		**Infected**	**Indeterminate**	**Not infected**
**Result of Test under Consideration**	**Positive**	A	D	G
	**Equivocal**	B	E	H
	**Negative**	C	F	I
	**No result**	Exclude from analysis[1]		

[1] *Note: If the test under consideration has "no result" because the test was not run and no attempt was made to test the sample, the test result will be excluded from primary analysis. If the ASIS result is invalid, this result will be excluded from the primary analysis.*

Table 12: Calculation of the positive and negative percent agreement: Aptima Combo 2® Assay (Hologic) and Abbott RealTime CT Assay (Abbott)

**Analysis Type**	**PPA (95% CI)**	**NPA (95% CI)**
**Primary Analysis**	A / (A+B+C)	I / (G + H + I)
**Sensitivity Analysis Scenarios**		
Classify indeterminates[1] using symptom status reported from [SIGNS AND SYMPTOMS]	A / (A+B+C+F)	I / (G + H + I + D)
Include all indeterminate tests as infected.	(A+D) / (A+B+C+D+E+F)	I / (G+H+I)
Include all indeterminate tests as not infected.	A / (A+B+C)	(I+F) / (D+E+F+G+H+I)
Consider indeterminate and equivocal test results as "missing", with the assumption of missing at random, and model the missing results. [2]	A / (A+C)	I / (G+I)

[1] *Classify indeterminate tests on the basis of symptom status. Include indeterminate tests as Infected if the participant is symptomatic in that compartment; include indeterminate tests as not infected in the participant is asymptomatic in that compartment.*
[2] *Cells B, D, E, F and H will be assigned or weighted to cells A, C, G and I based on modeling of the missing results.*

Table 13: Result of Test under Consideration versus ASIS: Xpert® CT/NG Assay (Cepheid) and Abbott RealTime NG Assay (Abbott)

		ASIS		
		Infected	Indeterminate	Not infected
**Result of Test under Consideration**	**Positive**	A	C	E
	**Negative**	B	D	F
	**No result/ Invalid**	Exclude from analysis[1]		

[1] *Note: If the test under consideration has "no result" because the test was not run and no attempt was made to test the sample, the test result will be excluded from primary analysis. If the ASIS result is invalid, this result will be excluded from the primary analysis.*

Table 14: Calculation of the positive and negative percent agreement: Xpert® CT/NG Assay (Cepheid) and for the Abbott RealTime NG Assay (Abbott)

**Analysis Type**	**PPA**	**NPA**
**Primary Analysis**	A / (A+B)	F / (C+E)
**Sensitivity Analysis Scenarios**		
Classify indeterminates[1] using symptom status reported from [SIGNS AND SYMPTOMS]	A / (A+B+D)	F / (C+E+F)
Include all indeterminate tests as infected.	(A+C) / (A+B+C+D)	F / (E+F)
Include all indeterminate tests as not infected.	A / (A+B)	(D+F) / (C+D+E+F)
Consider indeterminate and equivocal test results as "missing", with the assumption of missing at random, and model the missing results. [2]	A / (A+B)	F / (E+F)

[1] *Classify indeterminate tests on the basis of symptom status. Include indeterminate tests as Infected if the participant is symptomatic in that compartment; include indeterminate tests as not infected in the participant is asymptomatic in that compartment.*
[2] *Cells C and D will be assigned or weighted to cells A, B, E and F based on modeling of the missing results.*

CONFIDENTIAL

5.4.4. Secondary (Global) Analyses

Note: Analysis programs may require independent results verification per CBAR PROG.10033.

The following will be conducted for the three diagnostic tests.

a. Table: Summary of estimated positive and negative predictive values (PPV and NPV).
b. Figure: Plot of predictive PPV and NPV estimates as a function of prevalence for each test (point estimates and 95% pointwise confidence bands)
c. Table/Figure: The 95% Score confidence interval estimates of positive and negative likelihood ratios with forest plot display (one plot per test).
d. Table/Figure: Sensitivity analyses estimating PPV, NPV, and positive and negative likelihood ratios where indeterminates are: a) counted as all infected, b) all not infected, and c) based on symptom status reported in [SIGNS and SYMPTOMS].

5.4.5. Subgroup Analyses

Note: Analysis programs may require independent results verification per CBAR PROG.10033.

For each of the three diagnostic tests, subgroup analyses will be conducted for males, females, symptomatic participants, and asymptomatic participants by pathogen and anatomic site. The analyses described in Sections 5.4.3 and 5.4.4 will be conducted for each group.

5.5. Exploratory Analyses

Application of methods for diagnostic benefit:risk analyses will be conducted, including BED-FRAME methodologies[8]. Analyses related to these objectives will be initiated upon completion of primary analyses.

CONFIDENTIAL

[8] Evans, S.R., Pennello, G., Pantoja-Galicia, N., Jiang, H., Hujer, A.M., Hujer, K.M., Manca, C., Hill, C., Jacobs, M.R., Chen, L. and Patel, R., 2016. Benefit-risk evaluation for diagnostics: a framework (BED-FRAME). Clinical Infectious Diseases, p.ciw329.

H. APPENDIX 1: CBAR SOP PROG.10033

Center for Biostatistics in AIDS Research (CBAR)	
**STANDARD OPERATING PROCEDURE**	
Title: Application Validation	
Document ID: PROG.10033	Document Version: 4
	Page 22 of 26

1. Purpose

This document provides procedures for validation of programs and applications developed at CBAR as part of work on clinical studies.

2. Scope and Applicability

This document is applicable to programs and applications developed and used by CBAR workforce members in conjunction with work with data from clinical studies.

3. Introduction

Validation of programs and applications used at CBAR to create or analyze datasets in conjunction with clinical studies ensures that analysis results accurately reflect the original source data and conform to analysis specifications. Using a risk-based approach to validation, CBAR has developed standard operating procedures (SOPs) that define minimum programming standards for coding, testing and validation for the range of programming applications used at CBAR. This document provides a reference to the specific programming standards that apply to specific types of programs or applications and outlines procedures for documenting their validation.

4. Definitions

- **CBAR program:** Any of the various types of programs or applications that are created and maintained by the CBAR Programming Core for use across CBAR for the purpose of creating reports, files, or SAS datasets with minimal input from users. This includes SAS table and format programs and reporting macros as well as UNIX and R packages (e.g., MAKETOX2 and the PIPS library of functions).
- **CBAR derived dataset program:** A program, created and maintained by the CBAR Programming Core, that creates one or more standardized derived datasets for use across CBAR, which does not represent a single CRF or codebook at the DMC.
- **CBAR macro:** A generic SAS macro, created and maintained by the CBAR Programming Core, that consists of flexible SAS code that can be easily tailored to individual studies for reporting purposes (i.e., CBAR reporting macro) or perform simple operations similar to SAS functions (i.e., CBAR autocall macro).
- **CBAR R program:** An R program used by a CBAR R package created or maintained by members of the CBAR Programming Core.
- **CBAR SAS format program:** A program, created and maintained by the CBAR Programming Core, that creates permanent SAS (in)format(s) for the CBAR format catalog.
- **CBAR SAS template program:** A program, created and maintained by the CBAR Programming Core, that defines one or more SAS ODS templates (e.g., a style template, ExcelXP tagset, or graphic template).
- **CBAR UNIX package:** A collection of UNIX programs and other files, created and maintained by members of the CBAR Programming Core, for use across CBAR for the purpose of creating reports, files, or SAS datasets with minimal input from users. This includes all modules included by the main executable program file. For example, the MAKE_SMR UNIX package contains the MAKE_SMR file, smr.sas, and all of the macro program files included by smr.sas, and the MAKEDATA downloading scripts are part of a CBAR UNIX Package.
- **CBAR UNIX program:** A UNIX shell script, created and maintained by members of the CBAR Programming Core, comprised of all modules utilized by the executable program file for use across CBAR for the purpose of creating reports, files, or SAS datasets with minimal input from users. For example: Both MAKEDATA and MAKEDATA_NOSTUDY are both CBAR UNIX programs within the CBAR UNIX package MAKEDATA.

CONFIDENTIAL

- **Clinical Study:** A clinical trial or observational study involving human subjects.
- **Miscellaneous CBAR Program:** A program or application, created and maintained by the CBAR Programming Core, that does not fall under the definition of any of the CBAR programs in Section 5.0 of PROG.10033 Application Validation.
- **SAS table program:** A SAS program, created and maintained by the CBAR Programming Core, which creates one or more SAS datasets from ASCII data to represent a single CRF or codebook from the DMC.
- **Workforce members:** Employees (both academic appointees and staff), and other persons whose conduct, in the performance of CBAR work is under the direct control of CBAR whether or not they are paid by Harvard University.

5. Procedure

The following table outlines the different types of programs or applications based on their intended purposes and programming language. The table provides the CBAR SOP that defines the relevant programming standards for the development, testing, and validation of the program or application as well as the appropriate validation form that documents the validation process.

Formal validation of programs and applications not covered by the scope of relevant programming standards SOP is not required, but adherence to the practices described therein (as appropriate) is recommended.

Development, testing, and validation standards of CBAR programs and applications of a type not covered in the table below (miscellaneous CBAR programs) are at the discretion of the Head of the CBAR Programming Core. Validation review of these programs consists of 5 areas of focus: Source Code Control, Supporting Documentation, Program Code Review, Program Logic Review, and Testing Program Review, including Input and Output. Specific criteria within each of the 5 areas are at the discretion of the validation reviewer and are approved by the Head of the CBAR Programming Core.

**Type of Program/ Application**	**Programming Standards SOP**	**Validation Form**	**Personnel to be Notified upon Completion of Validation**
Study-Specific Derived Dataset Creation Programs (including format and macro programs)	PROG.10066 Study-Specific Derived Dataset Creation Programming Standards	PROG.10066.f1 Study-Specific Derived Dataset Creation Programming Review	Programmer
Study-Specific Analysis programs (including macro, template, and R programs)	PROG.10067 Study-Specific Analysis Programming Standards	PROG.10067.f1 Study-Specific Analysis Programming Review	Programmer
Study-Specific Fix Files	PROG.10030 Study-Specific Fix File Programming Standards	PROG.10030.f1 Study-Specific Fix File Programming Standards	Programmer
Study-Specific User Options Files	PROG.10071 Study-Specific User Options File Standards	PROG.10071.f1 Study-Specific User Options File Review	Programmer
SAS Table Programs	PROG.10029 SAS Table Programming Standards	PROG.10029.f1 SAS Table Programming Review	None
CBAR Derived Dataset Programs	PROG.10035 SAS Derived Dataset Programming Standards	PROG.10035.f1 SAS Derived Dataset Programming Review	Programmer, cbar.cda
CBAR SAS Format Programs	PROG.10037 SAS Format Programming Standards	PROG.10037.f1 SAS Format Programming Review	None
CBAR Macros	PROG.10034 SAS Macro Programming Standards	PROG.10034.f1 SAS Macro Programming Review	Programmer, head of CBAR programming core

CONFIDENTIAL

UNIX Programs	PROG.10031 UNIX Package Programming Standards	PROG.10031.f1 UNIX Package Programming Review	Programmer, head of CBAR programming core
CBAR R Programs	PROG.10036 R Programming Standards	PROG.10036.f1 R Programming Review	Programmer, head of CBAR programming core
CBAR SAS Template Programs	PROG.10038 SAS Template Programming Standards	PROG.10038.f1 SAS Template Programming Review	Programmer, head of CBAR programming core

5.1 Validation Form Submission

The completed and signed validation form is submitted to Document Management within 5 days of validation completion in one of the following ways:

- Place the form in the Document Management mailbox; or
- Mail the form to Document Management at CBAR; or
- Contact Document Management to arrange another method of submitting the signed form.

In the event that completion and signing of the validation form conflicts with the analysis reporting timeline, notification of validation completion may be documented via email to the required personnel. In this case, submission of the completed validation form to Document Management at CBAR occurs within 5 business days of distribution of the analysis report.

For the validation of miscellaneous CBAR programs, the validation criteria are submitted with the validation form.

5.2 Content of PROG.10033.f1 Miscellaneous CBAR Programming Review

- Program name and location
- Last date in change history
- Coding programmer
- Validation reviewer
- Head of the CBAR Programming Core
- Date of validation
- Version of PROG.10033 Application Validation used
- Miscellaneous CBAR Program Review
  - Review Criteria
  - Finding(s) during review
  - Resolution(s) prior to validation
- Signature of validation reviewer
- Date of signature
- Signature of Head of the CBAR Programming Core
- Date of signature

Referenced Documents

Document Title	Location
PROG.10029 SAS Table Programming Standards	Secure location on the CBAR network
PROG.10029.f1 SAS Table Programming Review	Secure location on the CBAR network
PROG.10030 Fix File Programming Standards	Secure location on the CBAR network
PROG.10030.f1 Fix File Programming	Secure location on the CBAR network

CONFIDENTIAL

PROG.10031 UNIX Programming Standards	Secure location on the CBAR network
PROG.10031.f1 UNIX Programming Review	Secure location on the CBAR network
PROG.10033.f1 Miscellaneous CBAR Programming Review	Secure location on the CBAR network
PROG.10034 SAS Macro Programming Standards	Secure location on the CBAR network
PROG.10034.f1 SAS Macro Programming Review	Secure location on the CBAR network
PROG.10035 SAS Derived Dataset Programming Standards	Secure location on the CBAR network
PROG.10035.f1 SAS Derived Dataset Programming Review	Secure location on the CBAR network
PROG.10036 SAS Template Programming Standards	Secure location on the CBAR network
PROG.10036.f1 SAS Template Programming Review	Secure location on the CBAR network
PROG.10037 SAS Format Programming Standards	Secure location on the CBAR network
PROG.10037.f1 SAS Format Programming Review	Secure location on the CBAR network
PROG.10038 R Programming Standards	Secure location on the CBAR network
PROG.10038.f1 R Programming Review	Secure location on the CBAR network
PROG.10066 Study-Specific Derived Dataset Creation Programming Standards	Secure location on the CBAR network
PROG.10066.f1 Study-Specific Derived Dataset Creation Programming Review	Secure location on the CBAR network
PROG.10067 Study-Specific Analysis Programming Standards	Secure location on the CBAR network
PROG.10067.f1 Study-Specific Analysis Programming Review	Secure location on the CBAR network
PROG.10071 Study-Specific User Options File Standards	Secure location on the CBAR network
PROG.10071.f1 Study-Specific User Options File Review	Secure location on the CBAR network

Version History

Version	Changes Made	Effective Date
1	Original Version	12/1/2013
2	Rationale: This version redefines Section 7. Version History to provide more information about the reason for the new version and the major changes included. This also more clearly describes the scope, procedure, and other parts of the SOP.<br>Purpose, Scope, Introduction, Definitions, Section 5: Clarification of language to better describe the scope and procedure; updating of outdated information and removal of unused definitions<br>Section 7: Format of section was changed	12/1/2013

CONFIDENTIAL

3	Purpose, Scope, and Introduction: Updated to clarify the rationale for validation and better define how the programming standards and validation procedure work together.<br>Definitions:<br>- The definition of CBAR program has been updated<br>- A new definition of a clinical study has been added.<br>Table: Minor changes to table headings<br>5.0 Procedure:<br>- Minor modifications to improve clarity<br>- Addition of standards to be defined by Head of Programming Core for CBAR programs not otherwise covered by current standards<br>- Notification of programmer and cbar.cda for SAS Table programs and SAS format programs have been removed<br>5.1 Validation Form Submission<br>- Order of the sequence of events changed to clarify that submission of the validation form occurs for the validation to be considered complete<br>- Time-frame for validation document submission removed since, per SOP, it is required for validation completion<br>- Flexibility is provided for validation form completion in the case of time-constraints	7/1/2014
4	Rationale: The updates made to this SOP for this version detail validation procedures for miscellaneous CBAR programs.<br>Definitions:<br>- Added definition of Miscellaneous CBAR program<br>5.0 Procedure:<br>- Added description of the validation review of miscellaneous CBAR programs<br>5.1 Validation Form Submission<br>- Described validation form submission for miscellaneous CBAR programs<br>- Further updates provided for validation form submission<br>5.2 Content of PROG.10033.f1 Miscellaneous CBAR Programming Review<br>- Added this section to describe the necessary fields for the creation of PROG.10033.f1.	12/1/2015

CONFIDENTIAL

ARLG
Antibacterial Resistance Leadership Group

Antibacterial Resistance Leadership Group (ARLG)

Performance of Nucleic Acid Amplification Tests for the Detection of *Neisseria gonorrhoeae* and *Chlamydia trachomatis* in Extragenital Sites

GC Statistical Analysis Plan
Version 2.0

Scott Evans, Lauren Komarow, Thuy Tran
Harvard T. H. Chan School of Public Health

Updated: April 12, 2018

(Version 1.0 finalized April 10, 2017)

INTRODUCTION

This document describes the content proposed for the statistical monitoring and primary statistical analysis of the study titled "Performance of Nucleic Acid Amplification Tests for the Detection of Neisseria gonorrhoeae (NG) and Chlamydia trachomatis (CT) in Extragenital Sites." The focus of this analysis will include the primary and secondary objectives to evaluate diagnostic accuracy of the following three nucleic acid amplification test (NAAT) platforms for the detection of NG and CT from the extragenital sites of the pharynx and the rectum:

- Xpert® CT/NG Assay [Cepheid]
- Combo 2 ® Assay [Hologic]
- Abott RealTime Chlamydia trachomatis and Neisseria gonorrhoeae assay [Abbott].

A subset of these analyses (as described herein) will form the basis of reports provided to the independent statistician as part of independent interim study monitoring while the study is ongoing. Therefore, this analysis plan includes a description of the key analyses which might lead to the modification of the study sample size, and hence also forms the core of any presentation or publication used to disseminate the primary conclusions of the study.

THE CURRENT STATISTICAL ANALYSIS PLAN (VERSION 2.0) IS BASED ON PROTOCOL VERSION 4.0 (DATED DECEMBER 4, 2017). PROTOCOL HISTORY

- Protocol Version 1.0 (April 25, 2016, Protocol ARLG_pNAAT-Yr3)
- Protocol Version 2.0 (August 1, 2016, Protocol ARLG_pNAAT-Yr3)
- **Protocol Version 3.0 (September 5, 2017, Protocol ARLG_pNAAT-Yr3)**
- **Protocol Version 4.0 (December 4, 2017, Protocol ARLG_pNAAT-Yr3)**

REVISIONS TO STATISTICAL ANALYSIS PLAN, VERSION 1.0 (FINALIZED APRIL 12, 2017):

- **Master-GC: Statistical Analysis Plan, Version 1.0 (April 10, 2017)**
    - **SAP was based on Protocol Version 2.0 (August 1, 2016, Protocol ARLG_pNAAT-Yr3)**

- **Master-GC: Statistical Analysis Plan, Version 2.0 (April 12, 2018)**
    - **SAP is based on Protocol Version 4.0 (December 4, 2017, Protocol ARLG_pNAAT-Yr3)**
    - **Updates include:**
        - **Final Analysis Considerations, pg 9**
            - **Clarification regarding final analysis population.**
            - **Includes exclusion of 167 participants from the final analysis population due to protocol deviations.**
        - **Swab Collection Completeness and Complications, pg 11**
            - **Clarification regarding swabs not collected per assigned order.**
        - **Swab Testing Completeness and Monitoring, pg 12**
            - **Added table to examine completeness of tiebreaker testing.**
        - **NAAT Test Results, pg 12**
            - **The "Possible Test Results" and "Notes" columns updated to reflect changes from Protocol Version 3.**
            - **Footnotes added to Tiebreaker assay section.**
        - **Anatomic Site Infection Status (ASIS) Determination, pg. 13**
            - **Row 42: Anatomic Site Infection Status was changed from "Indeterminate" to "Invalid, remove from analysis" to reflect changes from Protocol Version 3.**
        - **Table 9: Frequency of comparator NAAT result and tiebreaker result combinations by anatomical site and organism for test under consideration XX, pg 17**
            - **Added "2" to second column (omitted in the original SAP)**
        - **Final Analysis section, pg. 19**
            - **Section title updated from "Endpoint Definitions" to "Outcome Measures"**

- **Additional text clarifying data for final analysis was added to the Final Analysis Considerations, pg19.**
- **The following analysis tables were added to the Primary Analyses section, pg 19.**
  - **Frequency of re-tests by clinic and platform**
  - **Listing and frequency of re-test results by platform**
  - **Observed infection rate by clinic.**
- **Table 12: Calculation of the positive and negative percent agreement: Aptima Combo 2® Assay (Hologic) and Abbott RealTime CT Assay (Abbott), pg 21**
  - **Removed incorrectly labeled "(95% CI)" from PPA and NPA column headers**
  - **Added cell F to PPA denominator (was omitted in SAP, v1.0)**
  - **Added cell D to NPA denominator (was omitted in SAP, v1.0)**
- **Table 13: Result of Test under Consideration versus ASIS: Xpert® CT/NG Assay (Cepheid) and Abbott 21RealTime NG Assay (Abbott), pg. 21**
  - **For clarity, added "Detected" and "Not detected" to "Results of Test under Consideration" rows.**
  - **Removed "Invalid" from "No result" row to reflect changes from Protocol Version 3.**
- **Table 14: Calculation of the positive and negative percent agreement: Xpert® CT/NG Assay (Cepheid) and for the Abbott RealTime NG Assay (Abbott), pg 21**
  - **Added cell D to PPA denominator (was omitted in SAP, v1.0)**
  - **Added cell F to NPA denominator (was omitted in SAP, v1.0)**

LIST OF ABBREVIATIONS

**ARLG**	Antibacterial Resistance Leadership Group
**ASIS**	Anatomic Site Infection Status
**CDC**	Center for Disease Control and Prevention
**CFR**	Code of Federal Regulations
**CT**	*Chlamydia trachomatis*
**DCRI**	Duke Clinical Research Institute
**DNA**	Deoxyribonucleic acid
**FDA**	Food and Drug Administration
**HIV**	Human immunodeficiency virus
**ICMJE**	International Committee of Medical Journal Editors
**ID**	Identification
**IFU**	Instructions for Use
**IRB**	Institutional Review Board
**ISRC**	Independent Study Review Committee
**LGBT**	Lesbian, gay, bisexual and transgender
**NAAT**	Nucleic acid amplification test
**NR**	This means no test was run and there is no result
**NPA**	Negative percent agreement
**NPV**	Negative predictive value
**NG**	*Neisseria gonorrhoeae*
**NIH**	National Institutes of Health
**OHRP**	Office of Human Research Protections
**PPA**	Positive percent agreement
**PPV**	Positive predictive value
**PI**	Principal Investigator
**RNA**	Ribonucleic acid
**rRNA**	Ribosomal ribonucleic acid
**SDMC**	Statistical and Data Monitoring Center
**STD**	Sexually transmitted diseases
**WHO**	World Health Organization

STUDY SCHEMA AND OBJECTIVES

<u>DESIGN</u>	A cross-sectional, single visit study to evaluate the diagnostic accuracy of three nucleic acid amplification tests (NAATs) for detection of *Neisseria gonorrhoeae* and *Chlamydia trachomatis* from a set of four swabs each collected from both the pharyngeal and rectal sites, respectively.
<u>DURATION</u>	This is a single visit study. It is estimated that the study will take between 6 and12 months after enrollment of the first participant to fully enroll.<br><br>Note: Study duration was updated to 12 to 24 months during the study.
<u>SAMPLE SIZE</u>	Up to 2,500 participants<br><br>Note: Sample size was increased to up to 3000 participants per recommended changes by the independent statistician during the study.
<u>POPULATION</u>	Symptomatic or asymptomatic male, female, or transgender participants:<br>o Who are patients attending a participating clinic for evaluation of sexually transmitted disease (STD) , and<br>o ≥18 years of age at date of screening, and<br>o Able and willing to provide informed consent, and<br>o Willing to comply with study procedures, including collection of 4 swabs each from the pharynx and rectum for NG and CT testing.
<u>NUMBER OF SITES</u>	Up to 10
<u>STRATIFICATION</u>	Randomization of the swab order will not be stratified. Swabs will be collected from all participants at each extragenital site.
<u>DIAGNOSTICS</u>	While the diagnostic accuracy of three NAATs will be evaluated, a total of four swab kits will be collected from both the pharyngeal and rectal sites, respectively (8 swabs in total). The swab kits associated with the NAATs under investigation will be tested using the corresponding laboratory test assay and test system as defined in **Table 1**. The remaining NAAT will only be performed in cases of discordant results and serve as the tiebreaker assay. There will be no evaluation for diagnostic accuracy for the tie breaker NAAT.

1.1. <u>PRIMARY OBJECTIVES</u>

1.1.1. For each NAAT under evaluation, estimate the positive percent agreement (PPA) and negative percent agreement (NPA) for detection of the organism and extragenital site combinations listed below.

- *Neisseria gonorrhoeae* in rectal swabs
- *Neisseria gonorrhoeae* in pharyngeal swabs
- *Chlamydia trachomatis* in rectal swabs
- *Chlamydia trachomatis* in pharyngeal swabs

1.2. <u>SECONDARY OBJECTIVES</u>

1.2.1. Global analyses

For each NAAT under evaluation, positive predictive values (PPVs), negative predictive values (NPVs), positive likelihood ratios, and negative likelihood ratios will be calculated for detection of the organism and extragenital site combinations listed below.

- *Neisseria gonorrhoeae* in rectal swabs
- *Neisseria gonorrhoeae* in pharyngeal swabs
- *Chlamydia trachomatis* in rectal swabs
- *Chlamydia trachomatis* in pharyngeal swabs

1.2.2. Subgroup analyses

For each NAAT under evaluation, to estimate the PPAs, NPAs, PPVs, and NPVs for detection of NG and CT from rectal and pharyngeal swab specimens by sex and by anatomic site-specific symptom status.

1.3. EXPLORATORY OBJECTIVES

1.3.1. Application of developed diagnostic benefit:risk analyses, including BED-FRAME methodologies[1].

Table 1: List of Corresponding Swab Collection Kits, Laboratory Assay and Laboratory Machine

Swab Collection Kit Name	Corresponding Laboratory Test Assay	Corresponding Laboratory Test System (Machine)
NAATs Under Investigation (as defined in Section 1.2 of Protocol)		
**Cepheid** Xpert® CT/NG Vaginal/ Endocervical Specimen Collection Kit	Xpert® CT/NG assay<br>A combination test using Real-time PCR technique to detect two noncontiguous chromosomal DNA regions from NG (NG2 and NG4) - both of which must be positive to yield a positive result - and one chromosomal DNA target from CT (CT1).	GeneXpert System
**Hologic** Aptima® Multitest Swab Specimen Collection Kit	Aptima® Combo 2 for CT/NG assay<br>A combination test using target capture, transcription mediated amplification, and dual kinetic assay to detect regions of the rRNA from the 16S rRNA of GC and the 23S rRNA from CT using labeled DNA probes.	Panther system
**Abbot** multi-Collect Specimen Collection Kit	Abbott RealTime CT/NG assay<br>A combination test that uses a real-time PCR assay to detect a highly-conserved region within the Opa gene of NG and two distinct regions within the CT cryptic plasmid DNA .	Abbott m2000 RealTime System
Tiebreaker Assay (as defined in Section 1.2 of Protocol)		
**Hologic** Aptima® Multitest Swab Specimen Collection Kit	Aptima® Chlamydia trachomatis assay and/or the Aptima® Neisseria gonorrhoeae assay<br>These tests use target capture, transcription mediated amplification, and hybridization protection assays to identify the presence of RNA from the organism of interest in the clinical sample. (Note: The targets from the 16S rRNA for both GC and CT are different than those used in the Combo 2® Assay.)	Tigris DTS system

[1] Evans, S.R., Pennello, G., Pantoja-Galicia, N., Jiang, H., Hujer, A.M., Hujer, K.M., Manca, C., Hill, C., Jacobs, M.R., Chen, L. and Patel, R., 2016. Benefit-risk evaluation for diagnostics: a framework (BED-FRAME). Clinical Infectious Diseases, p.ciw329.

ANALYSIS PLAN OVERVIEW

A. General Analysis Considerations ... 8

B. Statistical Monitoring Considerations ... 8

C. Interim Analysis Considerations ... 8

D. Final Analysis Considerations ... 9

E. Report Distribution List ... 10

F. Application Validation ... 10

G. Analysis Plan ... 10

1. Study Population ... 10

1.1. Accrual and Eligibility Violations ... 10
1.2. Study Population Characteristics ... 10

2. Study Status ... 11

3. Swab Collection Completeness and Monitoring ... 11

3.1. Swab Collection Completeness and Complications ... 11
3.2. Laboratory Device Monitoring ... 12

4. Swab Testing Completeness and Monitoring ... 12

5. Infection Status by Anatomic Site and Organism ... 12

5.1. NAAT Test Results ... 12
5.2. Anatomic Site Infection Status (ASIS) Determination ... 13
5.3. Interim Analysis ... 15
5.4. Final Analysis ... 19
5.5. Exploratory Analyses ... 22

H. APPENDIX 1: CBAR SOP PROG.10033 ... 23

A. General Analysis Considerations

Data summaries and analyses will be presented overall and by sex or, where appropriate, by anatomic site and organism.

Because the Master-GC study is a single visit study (i.e, no follow-up), the only date captured in this study is the date of a participant's clinic visit (recorded on the DEMOG case report form) and represents both the "study entry" and "off study" dates.

To ensure participant confidentiality, any listing of individual patient level data will be minimized as much as possible during study monitoring and for interim and final analyses. If such data are provided, they will be indexed with a unique blinded identifier or with identifiers removed. Study dates will not be presented.

Of note, while participants may need to seek additional care beyond this single visit, this is beyond the purview of the study. Similarly, this study will not be used to inform or determine treatment practices.

B. Statistical Monitoring Considerations

Routine statistical monitoring of accrual, study conduct, and completeness of swab and data collection will be conducted by the Harvard statistical team. A monitoring report summarizing these components will be distributed by the Harvard team on a bi-monthly basis until completion of study accrual; see sections listed below for details. The report will be distributed to the core protocol team (see Section E for details). It is planned at this time that distribution of the first monitoring report will occur two weeks after transfer of data from DCRI to Harvard has been tested and successfully confirmed by both groups.

1. Study Population (Section G, Part 1, pg 10)
2. Study Status (Section G, Part 2, pg 11)
3. Swab Collection Completeness and Monitoring (Section G, Part 3, pg 11)
4. Swab Testing Completeness (Section G, Part 4, pg 12)

Additional monitoring and querying of all data will be conducted concurrently with the statistical monitoring report to ensure cross-form consistency and data quality.

C. Interim Analysis Considerations

The Master-GC study will undergo interim review every 500 participants or every 3 months after the enrollment of the first participant (whichever occurs first) by an independent statistician who will not have an association with the protocol or device companies.

The study cannot be stopped at the interim analysis for reaching regulatory goals in order to preserve error rates / coverage probability and to ensure enough data for subgroup analyses. Since the trial cannot be stopped for attainment of the regulatory goal, no adjustment to confidence levels is necessary.

Infection rates will be evaluated by the independent statistician to determine whether sample size adjustments are warranted to ensure sufficient number of infected participants to estimate PPAs with desired precision. The sample size will not be adjusted based on the observed PPAs and NPAs (which will not be reviewed while the study is ongoing). If infection is more prevalent than expected, a smaller sample size may be accepted. If infection is rarer than anticipated, then increases to sample size will be considered. Detailed analysis considerations are provided in Section 5.3.

The independent statistician will also monitor study accrual and review endpoint evaluability with particular focus on the frequency of tests with equivocal results, invalid results, or no results for each platform. Sample size adjustments may also be considered if there is evidence that results categorized as equivocal, invalid, or no results will impact study accrual time and/or data analyses.

Two separate analysis reports will be prepared and distributed for each interim review. The summary below provides the sections of this analysis plan that are relevant to each report; see sections for details.

<u>Open Administrative Report:</u> This report will be distributed to the independent statistician, core study team and DMID representative (see Section E for details). Presentation of data by swab or platform will be minimized as much as possible; any inclusion will use generic identifiers (i.e., platform or swab 1, 2, etc.) as appropriate. The report will include summaries of:

1. Study Population (Section G, Part 1, pg 10)
2. Study Status (Section G, Part 2, pg 11)
3. Swab Collection Completeness and Monitoring (Section G, Part 3, pg 11)
4. Swab Testing Completeness (Section G, Part 4, pg 12)

Closed Administrative Report: This report will be distributed only to the independent statistician (see Section E). The report will include analysis results from all assay platforms (swab and platform names will be presented).

1. Study Population (Section G, Part 1, pg 10)
2. Study Status (Section G, Part 2, pg 11)
3. Swab Collection Completeness and Monitoring (Section G, Part 3, pg 11)
4. Swab Testing Completeness (Section G, Part 4, pg 12)
5. Infection Status by Anatomic Site and Organism (Section G, Part 5, pg 12)

Of note, some analyses, tables or figures may be omitted at interim analyses if there are insufficient data to warrant analysis. Additional analyses may be provided if requested by the independent statistician.

Upon completing review of the interim analysis reports, it is anticipated that the independent statistician will provide recommendations to the protocol chair, protocol clinician and project lead. The choice of sharing these recommendations with members of protocol team will be left to the discretion of this latter group.

D. Final Analysis Considerations

The primary analysis will be conducted once data from the last participant enrolled has been received. The final analysis report will be distributed to the protocol team after Harvard receives the final, locked data transfer from DCRI and validation of primary analyses and internal review at Harvard are completed. The protocol team is defined in Section E. **The Final Analysis Report will include the following components.**

1. **Study Population (pg 10)**
2. **Study Status (pg 11)**
3. **Swab Collection Completeness and Monitoring (pg 11)**
4. **Swab Testing Completeness (pg 12)**
5. **Infection Status by Anatomic Site and Organism (pg 12, excludes Exploratory Analyses section)**

The final analysis population will include participants who meet all eligibility criteria **and are assigned a randomized swab order, and** provide four swabs from at least one anatomic site. **However, during the course of the study, the team discovered that swabs collected from 167 participants were not stored at the correct temperature per protocol before lab testing. As a result, the final analysis population will exclude all results from these 167 participants. The exclusion of these participants assumes data are missing at random (MAR) as the temperature deviation was random and not related to the lab test results. We do not expect the exclusion of these 167 participants to bias estimates of PPA and NPA, though some loss in precision is possible. Sensitivity analyses will not be conducted as part of final analysis unless requested otherwise by the team.**

For all final analyses, data collected from eCRFs or data entered by the laboratories in the laboratory information management system (LIMS) will be used. The primary analysis will evaluate the result for each diagnostic test (i.e., test under consideration) and compare with the ASIS for each anatomic site and organism combination. For each diagnostic test, positive percent agreement (PPA) and negative percent agreement (NPA) will be estimated using 95% confidence intervals. Confidence intervals will be estimated using the Score method. The primary analysis will also follow FDA guidance for incorporating indeterminate ASIS or test results for the test under consideration that are equivocal. As a result, all combinations of Infected/Indeterminate/Not Infected with all outcomes from the test under consideration (Positive/Equivocal/Negative/No result or Invalid) will be presented. If the test under consideration has “no result” because the test was not run and no attempt was made to test the sample, it will be excluded from the primary analysis.

Sensitivity analyses associated with the primary analyses will be conducted to examine the impact of different classifications of indeterminate results (i.e., all infected, all not infected, account for symptom status). Additional subgroup and secondary analyses are planned.

E. Report Distribution List

Unless otherwise mentioned, distribution lists for monitoring and analysis reports are comprised of:

Protocol Team: arlg.gc@mc.duke.edu members

Core Team: Protocol Chair, Protocol Clinician, Project Lead, Statisticians, Data Management, Clinical Trials Manager, Clinical Research Associate, Regulatory Associate.

DMID: DMID representative

DIR: Designated Independent Reviewer

Table 2: Report Distribution Summary

Report	Frequency	Distribution List
Harvard Monitoring Report	Bi-monthly (or as determined by core team)	Core Team
Open Administrative Interim Report	Every 3 months or every 500 participants (whichever comes first)	DIR, Core Team, DMID
Closed Administrative Interim Report	Every 3 months or every 500 participants (whichever comes first)	DIR, Statisticians
Final Analysis Report	End of study per study timeline	Protocol Team

F. Application Validation

All study-specific programs for creation of derived datasets for derivation of the primary outcomes defined in this document will require application validation per standing operating procedures (SOP) defined in CBAR PROG.10033 as appropriate; when applicable, requirements for independent results verifications of these datasets and application validation requirements for analysis programs are provided as annotations throughout the analysis plan. A copy of CBAR PROG.10033 is provided in Section H.

G. Analysis Plan

Throughout, annotations in square brackets ([xxx]) provide the data source.

1. Study Population

1.1. Accrual and Eligibility Violations

1. Table: Number (%) enrolled overall and by month and site.

   Note: Dates of first and last enrollments will be provided in a footnote to the table.

2. Table: Number (%) enrolled by month, site and reported sex at birth [DEMOG].
3. List: Description of violations of eligibility criteria if applicable. [INCEXC]

   Note: Participants enrolled and later found ineligible will be excluded from all analyses and be included in this listing. Information on whether swabs were collected for these participants will be noted.

4. Figure: Observed, cumulative and targeted accrual by month.

   Note: Targeted accrual is assumed to be approximately 209 participants per month for 12 months or 417 participants per month for 6 months to achieve full accrual of 2500 participants.

1.2. Study Population Characteristics

Table summaries will present the following study population characteristics overall and by sex. All variables, as noted below, will be analyzed on the continuous scale or as categories or both as appropriate.

For continuous variables, summary statistics will include # of participants, # of missing data points, mean and standard deviation, median (Q1-Q3), P10 and P90, and minimum and maximum.

For categorical variables, summary statistics will include number (%) for each category. In calculation of percentages, participants with missing data will not be included in the denominator.

1. Demographics
   a. Sex at birth: By category (male/female) [DEMOG]
   b. Gender: By category (man, woman, transman, transwoman, genderqueer, additional category, decline to answer) [DEMOG]
   c. Self-reported race, ethnicity and race/ethnicity as defined by NIH reporting standards: By category [DEMOG]
   d. Current age on day of study entry (years): Continuous and by age group (18-29, 30-39, 40-49, 50-59, 60+) [DEMOG]
2. Health Status [*For interim and final analyses only*]
   a. Abnormalities or symptoms in the pharynx in the past 7 days: By category (Yes/No) [SIGNS AND SYMPTOMS]
   Note: If yes, sub-categorization of the reported symptom will also be provided. This includes sore throat, painful swallowing, swollen/tender lymph nodes in the neck, and other symptom (with listed reasons).
   b. Abnormalities or symptoms in the rectum in the past 7 days: By category (Yes/No) [SIGNS AND SYMPTOMS]
   Note: If yes, sub-categorization of the reported symptom will also be provided. This includes rectal discharge, rectal bleeding, rectal itching, painful bowel movements, and other symptom (with listed reasons).

2. Study Status

As noted, the date of “study entry” is the same as the “off study” date. Study status will be determined by cross checking the study completion form with the sample collection form.

1. Table: Number (%) by category of study status.

   Categories: Completed study per sample collection form; incomplete- subject withdrew consent; incomplete – investigator decision; other reasons (with listing of reasons). [STUDY COMPLETION AND SAMPLE COLLECTION]

   Note: Study completion is defined as collection of at least four swabs from one anatomic site.

3. Swab Collection Completeness and Monitoring

All tables will be presented by anatomic site.

Of note, swab collection data will also be examined by study site and presented if low data completeness or high numbers of complications are observed.

3.1. Swab Collection Completeness and Complications

1. Table: Number (%) of participants reporting collected swabs.

   Categories: All 4 swabs, 3 swabs, 2 swabs, 1 swab, no swabs. [SAMPLE COLLECTION]

   Note: [CLOSED administrative report at interim analysis and final analyses only]: List swabs by name for each numeric category.
2. Listing/Table: Number (%) of participants with swabs not collected per assigned swab order. [SAMPLE COLLECTION]

   Note: If few deviations are reported, list the reported order the swabs were collected. ***Swabs not collected per assigned order will still contribute to interim and final analyses.***
3. Table: Number (%) of participants reporting sample collection complications.

Categories: Patient declined – due to excessive discomfort; patient declined – other reason; problem with testing materials, other (with listed reasons). [SAMPLE COMPLICATIONS]

Note: If few complications are reported, include the number of swabs collected (4, 3, 2, 1 or no swabs) for each complication. For CLOSED administrative report at interim analysis and final analyses only, list by swab name.

3.2. Laboratory Device Monitoring

The testing laboratories will maintain a log of all unanticipated device-related complications leading to no test, such as absence of transport media, quantity not sufficient, interference issues during testing, specimen transport collection system damage or incorrect transport system. No additional monitoring will be conducted.

4. Swab Testing Completeness and Monitoring

1. Table: Number (%) of participants with swabs tested (presented by anatomic site).

   Categories: All 4 swabs tested; 1-3 swabs tested; no swabs tested. [LAB DATA]

   For participants with fewer than 4 swabs tested, reasons why the laboratory did not test the swabs will be listed. [**Where available, summarize reasons from the lab issue tracking log;** LAB DATA]

2. **Table: Number (%) of participants with tiebreaker swabs tested (presented by anatomic site).**

   Categories: All 4 swabs tested; 1-3 swabs tested; no swabs tested. [LAB DATA]

5. Infection Status by Anatomic Site and Organism

[NOTE: Interim (Closed Report) and Final Analyses Only]

5.1. NAAT Test Results

Possible test results for each NAAT platform (as listed in **Table 3**) will be used for each anatomic site (pharynx or rectum) and organism (NG or CT) combination. These four combinations include NG of pharynx, CT of pharynx, NG of rectum, and CT of rectum.

Of note, if an expected repeat test result is missing, then the final test result will be derived as "NO RESULT" for the ASIS determination. This does not apply, however, to an initial test result of "EQUIVOCAL". In this case, if the expected repeat test result is missing, then the final result for ASIS determination will be "EQUIVOCAL".

Table 3: Summary of NAAT Test Results

NAAT	Possible Test Results	Notes
Xpert® CT/NG Assay (Cepheid)[2]	1. Not detected<br>2. Detected<br>3. Invalid (sample processing control or sample adequacy control failed)<br>4. Error (probe check control failed)<br>5. No result (insufficient data was collected, e.g. test aborted).	Initial invalid, error, or no result tests will be repeated. If the repeat test returns invalid, error, or no result, the final result will be considered an invalid and will be categorized as no result (NR) for the ASIS determination. If the repeat test returns not detected (negative) or detected (positive), this will be the result used for the ASIS determination.
Combo 2 ® Assay (Hologic)[3]	1. Negative<br>2. Positive<br>3. Equivocal (result between positive and negative)<br>**4. Invalid (run status is FAIL or other technical failure)**<br>**5. Error (sample was not tested due to an**	Initial equivocal, invalid, **and error** test results will be repeated. **If the repeat test result returns equivocal, the final test result will be considered an <u>equivocal test result</u> for the ASIS determination. If the repeat test result returns invalid or error, the final test result will be categorized as <u>equivocal</u> if the initial test was equivocal and as <u>no result (NR)</u> if**

[2] Cepheid. Xpert CT/NG Assay package insert. Vol. 301-0234, Rev B (Cepheid, 2013).
[3] Hologic. Aptima Combo 2 Assay package insert. Vol. 201798 Rev D (2012).

	**error detected by the instrument).**	**the initial test was invalid or error for the ASIS determination.** If the repeat test returns negative or positive, this will be the result considered for the ASIS determination.
Abbott RealTime CT/NG assay (Abbott)[4]	For NG:<br>1. Positive (detected, with cycle number less than or equal to the assay cut-off)<br>2. Negative (no evidence of amplification or cycle number greater than the assay cut-off).<br>**3. Error**<br>***Note: An equivocal interpretation does not apply.***<br><br>For CT:<br>1. Positive (detected, with cycle number less than or equal to the assay cut-off)<br>2. Negative (no evidence of amplification)<br>3. Equivocal (cycle number beyond the assay cut-off).<br>**4. Error**	**A sample with initial interpretation of error (both CT and NG) or equivocal (CT only) will be retested. If the repeat test returns negative or positive, this will be the result considered for the ASIS determination and statistical analyses. If the repeat test result is equivocal (CT only), the final test result will be considered equivocal for the ASIS determination and statistical analyses below. If the repeat test result is error, the final test result will be categorized as no result (NR) for the ASIS determination and statistical analyses below if the initial test result was error and as equivocal if the initial test result was equivocal (CT only).**
Tiebreaker assays: Aptima® Chlamydia trachomatis assay (Gen-Probe) and the Aptima® Neisseria gonorrhoeae assay (Gen-Probeye)[5, 6]	1. Negative<br>2. Positive<br>3. Equivocal (result between negative and positive ranges)<br>4. **Invalid (run status is FAIL or other technical failure)**<br>5. **Error (sample was not tested due to an error detected by the instrument)**	Initial equivocal, invalid **and error test results** test results will be repeated. **If the repeated test result is equivocal, it will be considered an <u>equivocal test result</u> for the ASIS determination. If the repeat test result returns invalid or error, the final test result will be categorized as <u>equivocal</u> if the initial test was equivocal and as <u>no result (NR)</u> if the initial test was invalid or error for the ASIS determination.** If the repeat test returns negative or positive, this will be the result considered for the ASIS determination.

5.2. Anatomic Site Infection Status (ASIS) Determination

Per protocol, determination of the ASIS will be NAAT-specific and evaluated for each anatomic site and organism combination.

Possible ASIS outcomes include:

- Infected
- Not infected
- Indeterminate
- Invalid, exclude from analysis

The anatomic site is considered to be **infected** when both reference test results are positive/**detected**.

The anatomic site is considered to be **not infected** when both reference test results are negative/**not detected**.

If there is discordance between the reference tests, an additional NAAT test will be performed as a tiebreaker. In this case, agreement of 2/3 of the reference NAATs will determine the ASIS. If two tests are equivocal or one equivocal and one not run, the third test result will stand as the ASIS if positive or negative. If two tests are not run, the ASIS will be considered invalid and will be excluded from the analysis.

---

[4] Abbott. Abbott RealTime CT/NG Package Insert. Vol. Ref 8L07-91 (2010).
[5] Gen-Probe. APTIMA Chlamydia trachomatis Assay. Vol. 501799 Revision D (San Diego, 2012).
[6] Gen-Probe. APTIMA Neisseria gonorrhoeae Assay. Vol. 502486 Revision A 73 (San Diego, 2011).

All possible test result combinations are shown in **Table 4**. The tiebreaker test will be run by the lab if any NAAT is not concordant with the others and interpreted only in the case of discordant results between the two planned reference tests for each assay. As the tiebreaker test is not a combination test, the tiebreaker will only be run for the organism with disagreement (e.g. if NG disagrees and CT agrees, the tiebreaker will only be run for NG).

To determine the ASIS, the test result for each respective site (pharynx or rectum) and each organism (NG or CT) for each NAAT platform will be used.

Table 4: Determination of the Anatomic Site Infection Status (ASIS)
Note:
*E **=** equivocal result;
****NR = no result. This can occur either because the test result was invalid or because the test could not be run (e.g. too little sample, improperly shipped, no sample received).

Comparator NAAT 1 Result	Comparator NAAT 2 Result	Tiebreaker NAAT Result	Anatomic Site Infection Status (ASIS)
+	+	Not indicated	Infected
+	-	+	Infected
+	E*	+	Infected
+	NR**	+	Infected
+	-	-	Not infected
+	-	E	Indeterminate
+	-	NR	Indeterminate
+	E	-	Indeterminate
+	E	E	Infected
+	E	NR	Infected
+	NR	-	Indeterminate
+	NR	E	Infected
+	NR	NR	Invalid, remove from analysis
-	-	Not indicated	Not infected
-	+	-	Not infected
-	E	-	Not infected
-	NR	-	Not infected
-	+	+	Infected
-	+	E	Indeterminate
-	+	NR	Indeterminate
-	E	+	Indeterminate
-	E	E	Not infected
-	E	NR	Not infected
-	NR	+	Indeterminate
-	NR	E	Not infected
-	NR	NR	Invalid, remove from analysis
E	+	+	Infected
E	-	-	Not infected
E	+	-	Indeterminate
E	+	E	Infected
E	+	NR	Infected
E	-	+	Indeterminate
E	-	E	Not infected
E	-	NR	Not infected
E	NR	+	Infected
E	NR	-	Not infected
E	NR	E	Indeterminate
E	NR	NR	Invalid, remove from analysis
NR	+	+	Infected
NR	-	-	Not infected
NR	NR	Not indicated	Invalid, remove from analysis

NR	+	-	Indeterminate
NR	+	E	Infected
NR	+	NR	**Invalid, remove from analysis**
NR	-	+	Indeterminate
NR	-	E	Not infected
NR	-	NR	Invalid, remove from analysis
NR	E	+	Infected
NR	E	-	Not infected
NR	E	E	Indeterminate
NR	E	NR	Invalid, remove from analysis

5.3. Interim Analysis

5.3.1. Interim Analysis Considerations

The study cannot be stopped at the interim for reaching regulatory goals in order to preserve error rates / coverage probability and to ensure enough data for subgroup analyses. Since the trial cannot be stopped for attainment of the regulatory goal, no adjustment to confidence levels are necessary.

Although it is anticipated that swab collection and data completeness will be high for both anatomical sites, both the Intention-to-Diagnose (ITD) and modified Intention-to-Diagnose (mITD) infection rates[7], as described in **Table 5**, will be estimated. It is expected that disease prevalence (infection rate) of NG in the rectum, NG in the pharynx, and CT in the rectum will each be greater than 7.5% in the population under evaluation. Disease prevalence of CT in the pharynx is expected to be rare.

Table 5: Infection rate calculation for each test under consideration

**Intent-to-Diagnose (ITD) Infection Rate**	Number of infected ASIS results for test under consideration / Total number of ASIS results[1]
**Modified Intent-to-Diagnose (mITD) Infection Rate**	Number of infected ASIS results for test under consideration / Total number of ASIS results with exclusion of invalid results[2]
[1] *The denominator will include the sum of all infected, not infected, indeterminate and invalid ASIS results.* [2] *The denominator will include the sum of all infected, not infected, and indeterminate ASIS results.*	

Operationally, a range of scenarios for the unobserved data will be generated at each interim review to assist with the decision making regarding sample size adjustments. To ensure enough infected participants to estimate PPAs with desired precision, the lowest ITD/mITD infection rate across the three assays and the three organism/site combinations (except CT in the pharynx) will be used as the observed rate for interim analysis. Of note, it is assumed that there will be greater precision to evaluate NPA as it is expected that there will be more not-infected results than infected results for each anatomic site.

Table 6 and **Table 7** illustrate hypothetical scenarios when the observed infection rate after the first 1000 participants is rarer than anticipated (equal to 5%, **Table 6)** or more prevalent than anticipated (equal to 10%, **Table 7**), respectively. For both tables, a range of infection rates for the unobserved data (scenarios A-C) are also presented to demonstrate the probability and corresponding total sample sizes to obtain 150, 175 or 200 disease positive participants, respectively, at the end of study.

If infection is rarer than anticipated, then increases to sample size may be considered as demonstrated in scenario A from Table 6. In this case, should the observed prevalence of 5% remain unchanged for the unobserved data, then enrollment of approximately 3200 total participants would be needed to ensure at least 80% probability of obtaining 150 disease positive participants. However, should the infection rate increase to 9% (Scenario C, **Table** 6), then sample size adjustments may not be warranted.

Alternatively, if infection is more prevalent than expected, a smaller sample size may be considered as demonstrated in scenarios A and B from **Table 7**.

[7] Fundamental Concepts for New Clinical Trialists. A Evans, S. and A Ting, N. 9781420090871. https://books.google.com/books?id=G1IUPQAACAAJ. 2015. Taylor & Francis.

Sample size adjustments may also be considered if there is evidence that the number of ASIS results categorized as invalid will impact study accrual time and/or data analyses.

Table 6: Hypothetical illustration of simulated infection rates after first 1000 participants and observed prevalence of 5%

**Observed Response Rate and Count**			
**ITD infection rate at interim**	**Prevalence of Disease Positive (%)**	**Disease Positive (N)**	
	**5%**	**50 participants**	

**Example Scenarios for Unobserved Data**								
**Scenario**	**Assumed prevalence rate[1]**	**Average prevalence rate[2]**	**Estimated probability (P) and sample size (N) to obtain:**					
			**150 Disease+ total participants**		**175 Disease+ total participants**		**200 Disease+ total participants**	
			**P**	**N**	**P**	**N**	**P**	**N**
**A**	**5%**	**5%**	<50%	2500	<50%	2500	<50%	2500
			80%	3220	80%	3740	80%	4260
			90%	3340	90%	3860	90%	4380
**B**	**7%**	**6.2%-6.4%**	64%	2500	<50%	2500	<50%	2500
			80%	2589	80%	2956	80%	3323
			90%	2669	90%	3046	90%	3413
**C**	**9%**	**7.2%-7.6%**	80%	2232	76%	2500	<50%	2500
			90%	2302	80%	2519	80%	2807
			99%	2500	90%	2589	90%	2877

[1] The assumed prevalence rate for the remaining unobserved data. [2] Average prevalence rate is the average of the observed and assumed prevalence rates for the entire duration of study for a given sample size.

Table 7: Hypothetical illustration of simulated infection rates after first 1000 participants and observed prevalence of 10%

**Observed Response Rate and Count**			
**ITD infection rate at interim**	**Prevalence of Disease Positive (%)**	**Disease Positive (N)**	
	**10%**	**100 participants**	

**Example Scenarios for Unobserved Data**								
**Scenario**	**Assumed prevalence rate[1]**	**Average prevalence rate[2]**	**Estimated probability (P) and sample size (N) to obtain:**					
			**150 Disease+ total participants**		**175 Disease+ total participants**		**200 Disease+ total participants**	
			**P**	**N**	**P**	**N**	**P**	**N**
**A**	**10%**	**10%**	80%	1610	80%	1870	80%	2130
			90%	1670	90%	1930	90%	2190
			>95%	2500	>95%	2500	>95%	2500
**B**	**7%**	**8.1%-8.6%**	80%	1875	80%	2242	63%	2500
			90%	1955	90%	2322	80%	2609
			>95%	2500	>95%	2500	90%	2699
**C**	**5%**	**6.5%-7.3%**	80%	2220	<50%	2500	<50%	2500
			90%	2330	80%	2740	80%	3250
			>95%	2500	90%	2860	90%	3380

[1] The assumed prevalence rate for the remaining unobserved data. [2] Average prevalence rate is the average of the observed and assumed prevalence rates for the entire duration of study for a given sample size.

5.3.2. Interim Analyses

Note: Analysis programs will require independent results verification per CBAR PROG.10033.

a. Table: For each diagnostic assay, frequency of observed test outcomes by anatomical site and organism combination as shown in **Table 8**. [Data source: LAB DATA]

Note: Test results for each diagnostic assay were defined previously in Section 5.1.

Table 8: Frequency of observed test outcomes by anatomical site and organism combination

**NAAT: GeneXpert System Test Results**					
	Not detected	Detected	Invalid	Error	No result
***NG,* rectum**					
***NG,* throat**					
***CT,* rectum**					
***CT,* throat**					
**NAAT: Panther System Test Results**					
	Negative	Positive	Equivocal	Invalid	
***NG,* rectum**					
***NG,* throat**					
***CT,* rectum**					
***CT,* throat**					
**NAAT: Abbott m2000 Test Results**					
	Negative	Positive			
***NG,* rectum**					
***NG,* throat**					
	Negative	Positive	Equivocal		
***CT,* rectum**					
***CT,* throat**					
**NAAT: Tigris DTS system Test Results (TIEBREAKER)**					
	Negative	Positive	Equivocal	Invalid	
***NG,* rectum**					
***NG,* throat**					
***CT,* rectum**					
***CT,* throat**					

b. Table/Figure: Frequency of comparator NAAT result and tiebreaker result combinations to define ASIS as shown in **Table 9**. This will be conducted for each test under consideration and be presented by anatomical site and organism combination. There will be a total of three tables to reflect the three tests under consideration. [Data source: LAB DATA]

Note: Only combinations with a frequency of one or greater will be shown. Any combination not shown will indicate that this combination was not observed (i.e., frequency equal to 0).

Table 9: Frequency of comparator NAAT result and tiebreaker result combinations by anatomical site and organism for test under consideration XX

**Test Under Consideration: XXX**				
**Anatomical Site and Organism: *NG,* rectum**				
Comparator NAAT Result 1	Comparator NAAT Result **2**	Tiebreaker NAAT result	ASIS	Frequency (n)
+	+	Not indicated	Infected	XX
+	-	+	Infected	XX
(Remaining observed combinations as described in **Table 4**.)				
**Anatomical Site and Organism: *NG,* throat**				
Comparator NAAT Result 1	Comparator NAAT Result **2**	Tiebreaker NAAT result	ASIS	Frequency (n)
+	+	Not indicated	Infected	XX
+	-	+	Infected	XX
(Remaining observed combinations as described in **Table 4**.)				
**Anatomical Site and Organism: *CT,* rectum**				

Comparator NAAT Result 1	Comparator NAAT Result **2**	Tiebreaker NAAT result	ASIS	Frequency (n)
+	+	Not indicated	Infected	XX
+	-	+	Infected	XX
(Remaining observed combinations as described in **Table 4**.)				
**Anatomical Site and Organism: *CT*, throat**				
Comparator NAAT Result 1	Comparator NAAT Result **2**	Tiebreaker NAAT result	ASIS	Frequency (n)
+	+	Not indicated	Infected	XX
+	-	+	Infected	XX
(Remaining observed combinations as described in **Table 4**.)				

c. Table/Figure: Number (%) of observed total test results, ASIS results, and prevalence (i.e., infection) rate. This will be conducted for each test under consideration and be presented by anatomical site and organism combination as shown in **Table 10** below. [Data source: LAB DATA]

Table 10: Number (%) of observed total test results, ASIS results, and prevalence (i.e., infection) rate for each test under consideration by anatomical site and organism

**Test under consideration:** GeneXpert System								
**Comparator NAATs:** Abbott m2000 and Panther System<br>**Tiebreaker NAAT:** Tigris DTS System								
	Sample Size (N)		ASIS				Prevalence (P, %)	
	N (ITD)	N (mITD)	Infected	Not infected	Indeterminate	Invalid	P (ITD)	P (mITD)
***NG*, rectum**								
***NG*, throat**								
***CT*, rectum**								
***CT*, throat**								
**Test under consideration:** Panther System								
**Comparator NAATs:** Abbott m2000 and GeneXpert System<br>**Tiebreaker NAAT:** Tigris DTS System								
	Sample Size (N)		ASIS				Prevalence (P, %)	
	N (ITD)	N (mITD)	Infected	Not infected	Indeterminate	Invalid	P (ITD)	P (mITD)
***NG*, rectum**								
***NG*, throat**								
***CT*, rectum**								
***CT*, throat**								
**Test under consideration:** Abbott m2000								
**Comparator NAATs:** Panther System and GeneXpert System<br>**Tiebreaker NAAT:** Tigris DTS System								
	Sample Size (N)		ASIS				Prevalence (P, %)	
	N (ITD)	N (mITD)	Infected	Not infected	Indeterminate	Invalid	P (ITD)	P (mITD)
***NG*, rectum**								
***NG*, throat**								
***CT*, rectum**								
***CT*, throat**								

a. Table/Figure: Predicted infection rate summary for range of scenarios (see **Table 6** and **Table 7** described in Section 5.3.1).
b. Table: Observed frequency of tiebreaker run versus expected number of runs (number, %).

c. Table: Using Fischer's exact test, the association between of test results and randomized swab order will be examined. Associations will be conducted by anatomical site and organism combination.

5.4. Final Analysis

5.4.1. Outcome Measures

For each participant, primary and secondary endpoints will be defined for each anatomical site and organism. [Primary source data: SAMPLE COLLECTION, STUDY COMPLETION and LAB DATA]

a. Primary Endpoints

Note: Derived datasets relating to the derivation of these endpoints will undergo independent results verification in accordance with CBAR PROG.10033.

Anatomic site infection status is determined by the reference standard (described in Section 4.2).

- Infection status for Neisseria gonorrhoeae in the rectum as determined by each NAAT
- Infection status for Neisseria gonorrhoeae in the pharynx as determined by each NAAT
- Infection status for Chlamydia trachomatis in the rectum as determined by each NAAT
- Infection status for Chlamydia trachomatis in the pharynx as determined by each NAAT

5.4.2. Final Analysis Considerations

Note: All dataset derivation programs for this endpoint will undergo independent results verification in accordance with CBAR PROG.10033; validation requirements for analysis programs are stated below.

As noted previously, for all final analyses, data collected from eCRFs or data entered by the laboratories in the laboratory information management system (LIMS) will be used. Data from additional sources such as machine data or issue tracking logs may be reviewed for clarification, but will not be used for analysis.

The result for each diagnostic test will be compared with the ASIS for that anatomic site and organism. PPA and NPA will also be estimated for each diagnostic test with 95% confidence intervals. Confidence intervals will be estimated using the Score method.[8] If the test under consideration has "no result" because the test was not run and no attempt was made to test the sample, the test result will be excluded from primary analysis. If the ASIS result is invalid, this result will be excluded from the primary analysis.

FDA guidance documents will be followed as part of the primary analysis to incorporate indeterminate ASIS or test results for the test under consideration that are equivocal.[9] It is recognized that there are pros and cons to the manner in which indeterminates are handled and how these impact the resulting estimates of PPA and NPA. The primary analysis approach is the most conservative and is biased downwards. If PPA is >90% under this scenario, then the conclusion of PPA >90% is clear.

Sensitivity analyses will be conducted as appropriate to evaluate the impact of diagnostic accuracy for a range of scenarios addressing indeterminate ASIS results. This will include counting indeterminate ASIS results against the results for the test under consideration with all combinations of Infected/Indeterminate/Not Infected with all outcomes from the test under consideration (Positive/Equivocal/Negative/No result or Invalid) evaluated. These analyses may not be conservative for calculations of PPA and NPA.

5.4.3. Primary Analyses

The following will be conducted for the three diagnostic tests.

Note: Analysis programs will require independent results verification per CBAR PROG.10033.

a. Table: For each diagnostic assay **(including the tiebreaker assay),** frequency of observed test outcomes by anatomical site and organism combination. [See **Table 8** described in Section 5.3.2; data source: LAB DATA]

[8] FDA. Establishing the performance characteristics of in vivo diagnostics devices for Chlamydia trachomatis and/or Neisseria gonorrhoeae: screening and diagnostic testing. (2011).
[9] FDA. Establishing the performance characteristics of in vivo diagnostics devices for Chlamydia trachomatis and/or Neisseria gonorrhoeae: screening and diagnostic testing. (2011).

b. **Table: Frequency (%) of re-tests by clinic and platform. This will be conducted for each test under consideration and be presented by anatomical site and organism combination. [Data source: LAB DATA; DEMOG]**
c. **Table: Listing and frequency (%) of re-tests results by platform. This will be conducted for each test under consideration and be presented by anatomical site and organism combination. [Data source: LAB DATA]**
d. Table/Figure: Frequency of comparator NAAT result and tiebreaker result combinations to define ASIS. This will be conducted for each test under consideration and be presented by anatomical site and organism combination. There will be a total of three tables to reflect the three tests under consideration. [See **Table 9** previously described in in Section 5.3.2; data source: LAB DATA]
e. Table/Figure: Number (%) of observed total test results, ASIS results, and prevalence (i.e., infection) rate. This will be conducted for each test under consideration and be presented by anatomical site and organism combination. [See **Table 10** previously described in Section 5.3.2; data source: LAB DATA]
f. **Table: Observed infection rate by clinic. This will be conducted for each test under consideration and be presented by anatomical site and organism combination**. **[Data source: LAB DATA; DEMOG]**
g. Tables:
   - Cross comparison of number (%) of results of test under consideration versus ASIS;
   - Estimates of PPA and NPA with 95% Score confidence intervals;
   - Due to differences in PPA and NPA calculations for NAAT platforms that do not have an "equivocal" test result when it is the test under consideration, analysis results will be estimated and presented separately based on this distinction as follows:
     - **Table 11** and **Table 12**: Aptima Combo 2® Assay (Hologic) and Abbott RealTime CT Assay (Abbott).
     - **Table 13** and **Table 14**: Xpert® CT/NG Assay (Cepheid) and Abbott RealTime NG Assay (Abbott).
h. Figure: Plot of PPA and 95% confidence interval band versus proportion of indeterminate ASIS results assumed to be positive (range of 0 to 1); vice-versa for NPA plot.
i. Table: Using Fischer's exact test, the association between test results and randomized swab order will be examined. Associations will be conducted by anatomical site and organism combination.

Table 11: Result of Test under Consideration versus ASIS: Aptima Combo 2® Assay (Hologic) and Abbott RealTime CT Assay (Abbott)

		ASIS		
		**Infected**	**Indeterminate**	**Not infected**
**Result of Test under Consideration**	**Positive**	A	D	G
	**Equivocal**	B	E	H
	**Negative**	C	F	I
	**No result**	Exclude from analysis[1]		

[1] *Note: If the test under consideration has "no result" because the test was not run and no attempt was made to test the sample, the test result will be excluded from primary analysis. If the ASIS result is invalid, this result will be excluded from the primary analysis.*

Table 12: Calculation of the positive and negative percent agreement: Aptima Combo 2® Assay (Hologic) and Abbott RealTime CT Assay (Abbott)

**Analysis Type**	**PPA**	**NPA**
**Primary Analysis**	A / (A+B+C**+F**)	I / (G+H+I **+ D**)
**Sensitivity Analysis Scenarios**		
Classify indeterminates[1] using symptom status reported from [SIGNS AND SYMPTOMS]	A / (A+B+C+F)	I / (G+H+I+D)
Include all indeterminate tests as infected.	(A+D) / (A+B+C+D+E+F)	I / (G+H+I)
Include all indeterminate tests as not infected.	A / (A+B+C)	(I+F) / (D+E+F+G+H+I)
Consider indeterminate and equivocal test results as "missing", with the assumption of missing at random, and model the missing results. [2]	A / (A+C)	I / (G+I)

[1] *Classify indeterminate tests on the basis of symptom status. Include indeterminate tests as Infected if the participant is symptomatic in that compartment; include indeterminate tests as not infected in the participant is asymptomatic in that compartment.*
[2] *Cells B, D, E, F and H will be assigned or weighted to cells A, C, G and I based on modeling of the missing results.*

Table 13: Result of Test under Consideration versus ASIS: Xpert® CT/NG Assay (Cepheid) and Abbott 21RealTime NG Assay (Abbott)

		ASIS		
		Infected	Indeterminate	Not infected
**Result of Test under Consideration**	**Positive/Detected**	A	C	E
	**Negative/Not detected**	B	D	F
	**No result**	Exclude from analysis[1]		

[1] *Note: If the test under consideration has "no result" because the test was not run and no attempt was made to test the sample, the test result will be excluded from primary analysis. If the ASIS result is invalid, this result will be excluded from the primary analysis.*

Table 14: Calculation of the positive and negative percent agreement: Xpert® CT/NG Assay (Cepheid) and for the Abbott RealTime NG Assay (Abbott)

**Analysis Type**	**PPA**	**NPA**
**Primary Analysis**	A / (A+B**+D**)	F / (C+E**+F**)
**Sensitivity Analysis Scenarios**		
Classify indeterminates[1] using symptom status reported from [SIGNS AND SYMPTOMS]	A / (A+B+D)	F / (C+E+F)
Include all indeterminate tests as infected.	(A+C) / (A+B+C+D)	F / (E+F)
Include all indeterminate tests as not infected.	A / (A+B)	(D+F) / (C+D+E+F)
Consider indeterminate and equivocal test results as "missing", with the assumption of missing at random, and model the missing results. [2]	A / (A+B)	F / (E+F)

[1] *Classify indeterminate tests on the basis of symptom status. Include indeterminate tests as Infected if the participant is symptomatic in that compartment; include indeterminate tests as not infected in the participant is asymptomatic in that compartment.*
[2] *Cells C and D will be assigned or weighted to cells A, B, E and F based on modeling of the missing results.*

5.4.4. Secondary (Global) Analyses

Note: Analysis programs may require independent results verification per CBAR PROG.10033.

The following will be conducted for the three diagnostic tests.

a. Table: Summary of estimated positive and negative predictive values (PPV and NPV).
b. Figure: Plot of predictive PPV and NPV estimates as a function of prevalence for each test (point estimates and 95% pointwise confidence bands)
c. Table/Figure: The 95% Score confidence interval estimates of positive and negative likelihood ratios with forest plot display (one plot per test).
d. Table/Figure: Sensitivity analyses estimating PPV, NPV, and positive and negative likelihood ratios where indeterminates are: a) counted as all infected, b) all not infected, and c) based on symptom status reported in [SIGNS and SYMPTOMS].

5.4.5. Subgroup Analyses

Note: Analysis programs may require independent results verification per CBAR PROG.10033.

For each of the three diagnostic tests, subgroup analyses will be conducted for males, females, symptomatic participants, and asymptomatic participants by pathogen and anatomic site. The analyses described in Sections 5.4.3 and 5.4.4 will be conducted for each group.

5.5. Exploratory Analyses

Application of methods for diagnostic benefit:risk analyses will be conducted, including BED-FRAME methodologies[10]. Analyses related to these objectives will be initiated upon completion of primary analyses.

[10] Evans, S.R., Pennello, G., Pantoja-Galicia, N., Jiang, H., Hujer, A.M., Hujer, K.M., Manca, C., Hill, C., Jacobs, M.R., Chen, L. and Patel, R., 2016. Benefit-risk evaluation for diagnostics: a framework (BED-FRAME). Clinical Infectious Diseases, p.ciw329.

H. APPENDIX 1: CBAR SOP PROG.10033

Center for Biostatistics in AIDS Research (CBAR)	
**STANDARD OPERATING PROCEDURE**	
Title: Application Validation	
Document ID: PROG.10033	Document Version: 4
	Page 23 of 27

1. Purpose

This document provides procedures for validation of programs and applications developed at CBAR as part of work on clinical studies.

2. Scope and Applicability

This document is applicable to programs and applications developed and used by CBAR workforce members in conjunction with work with data from clinical studies.

3. Introduction

Validation of programs and applications used at CBAR to create or analyze datasets in conjunction with clinical studies ensures that analysis results accurately reflect the original source data and conform to analysis specifications. Using a risk-based approach to validation, CBAR has developed standard operating procedures (SOPs) that define minimum programming standards for coding, testing and validation for the range of programming applications used at CBAR. This document provides a reference to the specific programming standards that apply to specific types of programs or applications and outlines procedures for documenting their validation.

4. Definitions

- **CBAR program:** Any of the various types of programs or applications that are created and maintained by the CBAR Programming Core for use across CBAR for the purpose of creating reports, files, or SAS datasets with minimal input from users. This includes SAS table and format programs and reporting macros as well as UNIX and R packages (e.g., MAKETOX2 and the PIPS library of functions).
- **CBAR derived dataset program:** A program, created and maintained by the CBAR Programming Core, that creates one or more standardized derived datasets for use across CBAR, which does not represent a single CRF or codebook at the DMC.
- **CBAR macro:** A generic SAS macro, created and maintained by the CBAR Programming Core, that consists of flexible SAS code that can be easily tailored to individual studies for reporting purposes (i.e., CBAR reporting macro) or perform simple operations similar to SAS functions (i.e., CBAR autocall macro).
- **CBAR R program:** An R program used by a CBAR R package created or maintained by members of the CBAR Programming Core.
- **CBAR SAS format program:** A program, created and maintained by the CBAR Programming Core, that creates permanent SAS (in)format(s) for the CBAR format catalog.
- **CBAR SAS template program:** A program, created and maintained by the CBAR Programming Core, that defines one or more SAS ODS templates (e.g., a style template, ExcelXP tagset, or graphic template).
- **CBAR UNIX package:** A collection of UNIX programs and other files, created and maintained by members of the CBAR Programming Core, for use across CBAR for the purpose of creating reports, files, or SAS datasets with minimal input from users. This includes all modules included by the main executable program file. For example, the MAKE_SMR UNIX package contains the MAKE_SMR file, smr.sas, and all of the macro program files included by smr.sas, and the MAKEDATA downloading scripts are part of a CBAR UNIX Package.
- **CBAR UNIX program:** A UNIX shell script, created and maintained by members of the CBAR Programming Core, comprised of all modules utilized by the executable program file for use across CBAR for the purpose of creating reports, files, or SAS datasets with minimal input from users. For example: Both MAKEDATA and MAKEDATA_NOSTUDY are both CBAR UNIX programs within the CBAR UNIX package MAKEDATA.

- **Clinical Study:** A clinical trial or observational study involving human subjects.
- **Miscellaneous CBAR Program:** A program or application, created and maintained by the CBAR Programming Core, that does not fall under the definition of any of the CBAR programs in Section 5.0 of PROG.10033 Application Validation.
- **SAS table program:** A SAS program, created and maintained by the CBAR Programming Core, which creates one or more SAS datasets from ASCII data to represent a single CRF or codebook from the DMC.
- **Workforce members:** Employees (both academic appointees and staff), and other persons whose conduct, in the performance of CBAR work is under the direct control of CBAR whether or not they are paid by Harvard University.

5. Procedure

The following table outlines the different types of programs or applications based on their intended purposes and programming language. The table provides the CBAR SOP that defines the relevant programming standards for the development, testing, and validation of the program or application as well as the appropriate validation form that documents the validation process.

Formal validation of programs and applications not covered by the scope of relevant programming standards SOP is not required, but adherence to the practices described therein (as appropriate) is recommended.

Development, testing, and validation standards of CBAR programs and applications of a type not covered in the table below (miscellaneous CBAR programs) are at the discretion of the Head of the CBAR Programming Core. Validation review of these programs consists of 5 areas of focus: Source Code Control, Supporting Documentation, Program Code Review, Program Logic Review, and Testing Program Review, including Input and Output. Specific criteria within each of the 5 areas are at the discretion of the validation reviewer and are approved by the Head of the CBAR Programming Core.

Type of Program/ Application	Programming Standards SOP	Validation Form	Personnel to be Notified upon Completion of Validation
Study-Specific Derived Dataset Creation Programs (including format and macro programs)	PROG.10066 Study-Specific Derived Dataset Creation Programming Standards	PROG.10066.f1 Study-Specific Derived Dataset Creation Programming Review	Programmer
Study-Specific Analysis programs (including macro, template, and R programs)	PROG.10067 Study-Specific Analysis Programming Standards	PROG.10067.f1 Study-Specific Analysis Programming Review	Programmer
Study-Specific Fix Files	PROG.10030 Study-Specific Fix File Programming Standards	PROG.10030.f1 Study-Specific Fix File Programming Standards	Programmer
Study-Specific User Options Files	PROG.10071 Study-Specific User Options File Standards	PROG.10071.f1 Study-Specific User Options File Review	Programmer
SAS Table Programs	PROG.10029 SAS Table Programming Standards	PROG.10029.f1 SAS Table Programming Review	None
CBAR Derived Dataset Programs	PROG.10035 SAS Derived Dataset Programming Standards	PROG.10035.f1 SAS Derived Dataset Programming Review	Programmer, cbar.cda
CBAR SAS Format Programs	PROG.10037 SAS Format Programming Standards	PROG.10037.f1 SAS Format Programming Review	None
CBAR Macros	PROG.10034 SAS Macro Programming Standards	PROG.10034.f1 SAS Macro Programming Review	Programmer, head of CBAR programming core

UNIX Programs	PROG.10031 UNIX Package Programming Standards	PROG.10031.f1 UNIX Package Programming Review	Programmer, head of CBAR programming core
CBAR R Programs	PROG.10036 R Programming Standards	PROG.10036.f1 R Programming Review	Programmer, head of CBAR programming core
CBAR SAS Template Programs	PROG.10038 SAS Template Programming Standards	PROG.10038.f1 SAS Template Programming Review	Programmer, head of CBAR programming core

5.1 Validation Form Submission

The completed and signed validation form is submitted to Document Management within 5 days of validation completion in one of the following ways:

- Place the form in the Document Management mailbox; or
- Mail the form to Document Management at CBAR; or
- Contact Document Management to arrange another method of submitting the signed form.

In the event that completion and signing of the validation form conflicts with the analysis reporting timeline, notification of validation completion may be documented via email to the required personnel. In this case, submission of the completed validation form to Document Management at CBAR occurs within 5 business days of distribution of the analysis report.

For the validation of miscellaneous CBAR programs, the validation criteria are submitted with the validation form.

5.2 Content of PROG.10033.f1 Miscellaneous CBAR Programming Review

- Program name and location
- Last date in change history
- Coding programmer
- Validation reviewer
- Head of the CBAR Programming Core
- Date of validation
- Version of PROG.10033 Application Validation used
- Miscellaneous CBAR Program Review
  - Review Criteria
  - Finding(s) during review
  - Resolution(s) prior to validation
- Signature of validation reviewer
- Date of signature
- Signature of Head of the CBAR Programming Core
- Date of signature

Referenced Documents

Document Title	Location
PROG.10029 SAS Table Programming Standards	Secure location on the CBAR network
PROG.10029.f1 SAS Table Programming Review	Secure location on the CBAR network
PROG.10030 Fix File Programming Standards	Secure location on the CBAR network
PROG.10030.f1 Fix File Programming	Secure location on the CBAR network

PROG.10031 UNIX Programming Standards	Secure location on the CBAR network
PROG.10031.f1 UNIX Programming Review	Secure location on the CBAR network
PROG.10033.f1 Miscellaneous CBAR Programming Review	Secure location on the CBAR network
PROG.10034 SAS Macro Programming Standards	Secure location on the CBAR network
PROG.10034.f1 SAS Macro Programming Review	Secure location on the CBAR network
PROG.10035 SAS Derived Dataset Programming Standards	Secure location on the CBAR network
PROG.10035.f1 SAS Derived Dataset Programming Review	Secure location on the CBAR network
PROG.10036 SAS Template Programming Standards	Secure location on the CBAR network
PROG.10036.f1 SAS Template Programming Review	Secure location on the CBAR network
PROG.10037 SAS Format Programming Standards	Secure location on the CBAR network
PROG.10037.f1 SAS Format Programming Review	Secure location on the CBAR network
PROG.10038 R Programming Standards	Secure location on the CBAR network
PROG.10038.f1 R Programming Review	Secure location on the CBAR network
PROG.10066 Study-Specific Derived Dataset Creation Programming Standards	Secure location on the CBAR network
PROG.10066.f1 Study-Specific Derived Dataset Creation Programming Review	Secure location on the CBAR network
PROG.10067 Study-Specific Analysis Programming Standards	Secure location on the CBAR network
PROG.10067.f1 Study-Specific Analysis Programming Review	Secure location on the CBAR network
PROG.10071 Study-Specific User Options File Standards	Secure location on the CBAR network
PROG.10071.f1 Study-Specific User Options File Review	Secure location on the CBAR network

Version History

Version	Changes Made	Effective Date
1	Original Version	12/1/2013
2	Rationale: This version redefines Section 7. Version History to provide more information about the reason for the new version and the major changes included. This also more clearly describes the scope, procedure, and other parts of the SOP.<br>Purpose, Scope, Introduction, Definitions, Section 5: Clarification of language to better describe the scope and procedure; updating of outdated information and removal of unused definitions<br>Section 7: Format of section was changed	12/1/2013

3	Purpose, Scope, and Introduction: Updated to clarify the rationale for validation and better define how the programming standards and validation procedure work together.<br>Definitions:<br>- The definition of CBAR program has been updated<br>- A new definition of a clinical study has been added.<br>Table: Minor changes to table headings<br>5.0 Procedure:<br>- Minor modifications to improve clarity<br>- Addition of standards to be defined by Head of Programming Core for CBAR programs not otherwise covered by current standards<br>- Notification of programmer and cbar.cda for SAS Table programs and SAS format programs have been removed<br>5.1 Validation Form Submission<br>- Order of the sequence of events changed to clarify that submission of the validation form occurs for the validation to be considered complete<br>- Time-frame for validation document submission removed since, per SOP, it is required for validation completion<br>- Flexibility is provided for validation form completion in the case of time-constraints	7/1/2014
4	Rationale: The updates made to this SOP for this version detail validation procedures for miscellaneous CBAR programs.<br>Definitions:<br>- Added definition of Miscellaneous CBAR program<br>5.0 Procedure:<br>- Added description of the validation review of miscellaneous CBAR programs<br>5.1 Validation Form Submission<br>- Described validation form submission for miscellaneous CBAR programs<br>- Further updates provided for validation form submission<br>5.2 Content of PROG.10033.f1 Miscellaneous CBAR Programming Review<br>- Added this section to describe the necessary fields for the creation of PROG.10033.f1.	12/1/2015
